# Supplementary material for: Association between gabapentinoid treatment, concurrent use with opioid or benzodiazepine and the risk of drug poisoning: A self-controlled case series study
Source: PLoS Med. 2026 Apr 16;23(4):e1005035. doi: 10.1371/journal.pmed.1005035 (PMC13086301; doi:10.1371/journal.pmed.1005035)
Supplement: S1 Appendix — (DOCX) [file pmed.1005035.s001.docx]

**1) Comparison study between gabapentin and pregabalin**: Only individuals who took both gabapentin and pregabalin were included this analysis. Nine levels of risk windows were created to account for all possible combinations of gabapentin and pregabalin usage, as each drug has three risk windows (90 day before treatment period, treatment period, and non-use reference period). The incident rate of all-cause drug poisoning during the gabapentin only treatment period (when pregabalin risk window at reference) was compared to the pregabalin only treatment period (when gabapentin risk window at reference) (Table 2).

This stratified analysis effectively compares the risk of all-cause drug poisoning between pregabalin and gabapentin because it uses person-time from the same individuals, thereby eliminating between-individual confounders. By focusing on periods when each patient was exposed to one drug while the other was at reference, the analysis controls for individual-specific confounding factors that could influence the risk of all-cause drug poisoning.

We cannot simply compare the incident rate ratios (aIRRs) between separate cohorts of gabapentin-only and pregabalin-only users because these groups may differ in many ways (e.g. sociodemographic factors and underlying health conditions). By using a within-individual comparison, we ensure that these potential confounders do not bias the comparison between the two drugs.

**2) Interaction study between gabapentinoid and opioid**: Only individuals who took both gabapentinoids and opioids were included in this analysis (S2 Fig). Patients who took methadone or buprenorphine were removed from the cohort as they may take these medications for treatment of substance misuse, which may elevate the baseline risk of all-cause drug poisoning. 18 levels of risk windows were created to account for all possible combinations of gabapentinoid and opioid treatment, as each gabapentinoid treatment has 6 risk windows (90 days before treatment, first 28 days of treatment period, 29-56 days of treatment period, 57-84 days of treatment period, the remaining time of treatment period and the non-use reference period) and opioid treatment was divided into three risk windows (90 days before treatment, treatment period, and non-use reference period). The incident rate of all-cause drug poisoning during the different risk windows were compared to the incident rate of all-cause drug poisoning when both gabapentinoid and opioid are at reference period (Table 2, Fig 3 and S2 Fig).

We have specifically investigated the aIRRs of different gabapentinoid risk windows when opioid was at reference and under opioid treatment period. This allowed us to examine the risk of incident all-cause drug poisoning when the patient was taking gabapentinoid alone and taking gabapentinoid together with opioid, examining the change in risk during concurrent use.

**3) Interaction study between gabapentinoid and benzodiazepine**: Only individuals who took both gabapentinoids and benzodiazepines were included in this analysis (S2 Fig). 18 levels of risk windows were created to account for all possible combinations of gabapentinoid and benzodiazepine treatment, as each gabapentinoid treatment has 6 risk windows (90 days before treatment, first 28 days of treatment period, 29-56 days of treatment period, 57-84 days of treatment period, the remaining time of treatment period and the non-use reference period) and benzodiazepine treatment was divided into three risk windows (90 days before treatment, treatment period, and non-use reference period). The incident rate of drug poisoning during the different risk windows were compared to the incident rate of all-cause drug poisoning when both gabapentinoid and benzodiazepine are at reference period (Table 2, Fig 3 and S2 Fig).

We have specifically investigated the aIRRs of different gabapentinoid risk windows when benzodiazepine was at reference and under benzodiazepine treatment period. This allowed us to examine the risk of incident all-cause drug poisoning when the patient was taking gabapentinoid alone and taking gabapentinoid together with benzodiazepine, examining the change in risk during concurrent use.

**4) Interaction study between gabapentinoid, opioid and benzodiazepine:** Only individuals who took both gabapentinoids, opioids and benzodiazepines were included in this analysis. Each of gabapentinoids, opioids and benzodiazepines were classified into 3 risk windows, which are 90-day before treatment, treatment periods and other non-treatment reference periods. Together, 27 mutually exclusive combinations are formed, covering all patient time of included individuals. The incident rate of all-cause drug poisoning during the different risk windows were compared to the incident rate of all-cause drug poisoning when gabapentinoids, opioids and benzodiazepines were at reference period.

We have specifically investigated the aIRRs of risk windows when under benzodiazepine and gabapentinoid treatment but opioid at reference; under opioid and gabapentinoid treatment but benzodiazepine at reference; under treatment of all gabapentinoid, opioid and benzodiazepine. This allowed us to examine the risk of incident all-cause drug poisoning when the patient was taking gabapentinoid together with opioid and benzodiazepine, examining the change in risk during concurrent use.
