# Supplementary material for: Association between gabapentinoid treatment, concurrent use with opioid or benzodiazepine and the risk of drug poisoning: A self-controlled case series study
Source: PLoS Med. 2026 Apr 16;23(4):e1005035. doi: 10.1371/journal.pmed.1005035 (PMC13086301; doi:10.1371/journal.pmed.1005035)
Supplement: S2 Appendix — (DOCX) [file pmed.1005035.s002.docx]

Subgroup analyses were conducted to investigate the risk of all-cause drug poisoning in specific groups of patients after exposure to gabapentinoids.

**1) Stratified by sex**: aIRRs of all-cause drug poisoning in different risk windows were stratified by sex. This analysis is to examine whether the aIRRs would differ in different sex (Table 2 and S5 Fig).

**2) Stratified by types of gabapentinoid**: All-cause drug poisoning aIRRs were stratified by gabapentin only and pregabalin only, mutually exclusive. This analysis is to examine the aIRRs in patients who took gabapentin only and pregabalin only (Table 2 and Fig 2).

**3) Stratified by age groups**: The cohort was stratified by age groups (18-24, 25-34, 35-44, 45-54, 55-64, 65 or above) (S14 Table and S6 Fig). Only the observational periods that lie within each age group will be included. Patients included in the analysis will need to have outcome within the extracted observational periods. This analysis is to examine whether the aIRRs would differ in different age groups.

**4) Stratified by ethnic groups**: The cohort was stratified by ethnicity – Black, South Asian and White (S15 Table). This analysis is to examine whether the aIRRs would differ in different ethnic groups.

**5) Stratified by daily dose levels**: Daily doses, expressed in Defined Daily Doses (DDD) per day, were calculated by multiplying the daily dose from the CPRD by the formulation strength and dividing by the defined daily dose for gabapentin or pregabalin. When the daily dose information was missing, it was estimated by dividing the total quantity prescribed by the duration of treatment. In cases of overlapping prescriptions with different daily dose levels, the dose from the most recent prescription was used. Daily dose levels were categorized a) <0.5 DDD per day, b) between 0.5 and 1 DDD per day, and c) >1 DDD per day (S16 Table). Individuals whose daily doses spanned multiple daily dose categories during the observation period were excluded from the analyses.

**6) Stratified by psychiatric comorbidities**: Limiting the analysis to individuals who had mental health condition diagnoses before observation end, including substance misuse, bipolar and mania, depression, anxiety disorder, schizophrenia, other psychosis and insomnia, in CPRD Aurum or HES (S17 Table). Patients who have the above mental health conditions may behave differently to those who do not. This analysis is to examine whether limiting the cohort to those who developed mental health conditions would have a different aIRR. Patients who were not diagnosed with any mental health condition were also evaluated.

**7) Limit outcome event to accidental poisoning (ICD-10, X40-X44):** Definition of the study outcome was limited to incident accidental poisoning event. This will limit the number of individuals included in the study cohort but allow us to focus on patients who had accidental poisoning (S18 Table). Please be aware that the intention of drug poisoning may have potential misclassification issues.

**8) Limit outcome event to intentional self-poisoning (ICD-10, X60-X64**): Definition of the study outcome was limited to incident intentional self-poisoning event. This will limit the number of individuals included in the study cohort but allow us to focus on patients who had intentional self-poisoning (S18 Table). Please be aware that the intention of drug poisoning may have potential misclassification issues.
