# Supplementary material for: Association between gabapentinoid treatment, concurrent use with opioid or benzodiazepine and the risk of drug poisoning: A self-controlled case series study
Source: PLoS Med. 2026 Apr 16;23(4):e1005035. doi: 10.1371/journal.pmed.1005035 (PMC13086301; doi:10.1371/journal.pmed.1005035)
Supplement: S3 Appendix — (DOCX) [file pmed.1005035.s003.docx]

Sensitivity analyses were conducted to test the validity and robustness of the initial study results. Details of each analysis are described below:

**1) Spline-based SCCS analysis**: Nonparametric, spline-based SCCS analysis of 180 days before and 180 days after treatment initiation was performed (S4 Fig) [1]. The results of the spline-based SCCS allow us to visualize the change in incident risk of all-cause drug poisoning before and after start of gabapentinoid treatment. Time-varying confounders were not adjusted in this model, but all time-invariant confounders were adjusted.

**2) Excluding patients who were dead 6 months after event**: Patients who died within 6 months of event date were excluded from the analysis examine whether the self-controlled case series assumption — that the event does not affect the observation period—was violated (S19 Table) [2].

**3) Starting observation period from diagnosis of neuropathic pain or chronic pain**: The observation start was further defined as the first observed date of neuropathic pain or chronic pain diagnosis to examine the risk of all-cause drug poisoning among patients who were likely been prescribed with gabapentinoids for treatment of these diagnoses (S19 Table and S2 Fig). Patients who did not have a neuropathic pain or chronic pain diagnosis were excluded in the analysis.

**4) Limiting the cohort to patients with at least 2 prescriptions of gabapentinoid**: Patients with only one prescription of gabapentinoid within the observation period were removed as they may have poorer adherence to the exposure of interest. This addresses the issue of potential exposure misclassification (S19 Table).

**5) Changing the duration of before treatment period**: The risk window before treatment was changed from 90 days to 120 days and 60 days (S19 Table). This allows us to examine whether lengthening the before treatment risk window will affect the aIRR of this risk window.

**6) Do not adjust for concomitant medications**: Only age and season were adjusted in this sensitivity analysis (S19 Table). This analysis allows us to examine the effect of not adjusting for potential time-varying confounders such as use of antiseizure medications, opioids and psychiatric medications.

**7) Removing death cases in interaction studies between gabapentinoid and opioid/ benzodiazepine:** Patients who died within 6 months of event date were excluded from the analysis examine whether the self-controlled case series assumption that the event does not affect the observation period was violated (S20 Table) [2].

**8) Extension of treatment period**: Analyses were conducted based on different drug non-adherence scenarios. Each treatment period was further extended by adding 28 to 84 days (1 to 3 median lengths of gabapentinoid prescription) after the end of treatment periods to assess this effect (S9 Fig).

**9) Not combining GABA prescriptions that were less than or equal to 90 days apart**: Gabapentinoids that were less than or equal to 90 days apart were not combined in this analysis. Each prescription was treated as a distinct exposure interval without concatenation, and any time beyond the recorded prescription duration was classified as non-treated, even if the next prescription started shortly afterwards.

**10) SCCS event-dependent observation extension [3]**: The event-dependent observation extension of the SCCS model addresses situations where the observation period is influenced by the occurrence of the event of interest, such as when an event affects the likelihood of future observations or survival time. Performing this analysis can validate the results in the main analysis and examine whether SCCS assumptions were violated. In this model, age was the only time-varying confounder that can be adjusted in this model. All time-invariant confounders were adjusted in this model. Only 90 days before treatment period, treatment period and reference period were defined in this model (S21 Table).

**11) SCCS event-dependent exposure extension [4]:** The event-dependent exposure extension accounts for scenarios where the occurrence of an event affects the probability of subsequent exposures. Performing this analysis can validate the results in the main analysis and examine whether SCCS assumptions were violated. In this model, age was the only time-varying confounder that can be adjusted in this model. All time-invariant confounders were adjusted in this model. Only the first 28 day of treatment period, 29-56 day of treatment period, the remaining time of treatment period and reference period were defined in this model (S21 Table).

**12) Negative control analysis:** We conducted a negative control analysis to validate the use of food poisoning as an alternative outcome (S22 Table). To our knowledge, there is no published evidence or pharmacologic/biological hypothesis to support gabapentinoid use is associated to food poisoning. If association was found in this analysis, it would raise the possibility that our study design is prone to bias. We used the same setting and methods as the main analysis in the negative control analysis.

**13) Case-case-time-control analysis**: An additional case-case-time-control analysis was performed (S23 Table 23 and S3 Figure). It included two self-controlled analyses—a case crossover analysis and a control crossover analysis consisting of future cases [5]. The case crossover design eliminated time invariant confounders through within-individual comparisons [6]. The control crossover analysis was performed to adjust for time trends in drug use and to address the likely protopathic bias by including future cases [5]. We matched current cases to future cases by age, sex and ethnicity. For the future cases, we selected patients whose event dates were within 180-360 days after the matched event dates of the current cases. We defined the index dates as the event dates of the current cases and assigned the same index dates for the future cases. We divided the 180 days preceding the index dates into several 30-day intervals, including a hazard period (days −1 to −30), a washout period (days −31 to −60), and a reference period randomly selected from four different 30-day reference periods (between days −61 and −180) (S3 Figure).

To evaluate the risk of incident all-cause drug poisoning associated with gabapentinoid treatment, we used conditional logistic regression to estimate the odds ratios with 95% confidence intervals for exposure to gabapentinoids, comparing the hazard period with the reference period. Concomitant medications such as antiseizure medications, opioids, psychiatric medications and non-steroidal anti-inflammatory drugs were adjusted in the analysis. We calculated case-case-time-control odds ratios as the odds ratios from the case crossover analysis divided by the odds ratios from the control crossover analysis.

This analysis allows us to examine whether a different study design would generate results that differ from the main SCCS study design.

**References**

1. Ghebremichael-Weldeselassie Y, Whitaker HJ, Farrington CP. Spline-based self-controlled case series method. Stat Med. 2017;36(19):3022-38. Epub 20170503. doi: 10.1002/sim.7311. PubMed PMID: 28470682.

2. Petersen I, Douglas I, Whitaker H. Self controlled case series methods: an alternative to standard epidemiological study designs. BMJ. 2016;354:i4515. Epub 20160912. doi: 10.1136/bmj.i4515. PubMed PMID: 27618829.

3. Farrington CP, Anaya-Izquierdo K, Whitaker HJ, Hocine MN, Douglas I, Smeeth L. Self-controlled case series analysis with event-dependent observation periods. Journal of the American Statistical Association. 2011;106(494):417-26.

4. Farrington CP, Whitaker HJ, Hocine MN. Case series analysis for censored, perturbed, or curtailed post-event exposures. Biostatistics. 2009;10(1):3-16. Epub 20080521. doi: 10.1093/biostatistics/kxn013. PubMed PMID: 18499654.

5. Wang S, Linkletter C, Maclure M, Dore D, Mor V, Buka S, et al. Future cases as present controls to adjust for exposure trend bias in case-only studies. Epidemiology. 2011;22(4):568-74. doi: 10.1097/EDE.0b013e31821d09cd. PubMed PMID: 21577117; PubMed Central PMCID: PMCPMC3110688.

6. Delaney JA, Suissa S. The case-crossover study design in pharmacoepidemiology. Stat Methods Med Res. 2009;18(1):53-65. Epub 20080902. doi: 10.1177/0962280208092346. PubMed PMID: 18765504.
