# Supplementary material for: Association between gabapentinoid treatment, concurrent use with opioid or benzodiazepine and the risk of drug poisoning: A self-controlled case series study
Source: PLoS Med. 2026 Apr 16;23(4):e1005035. doi: 10.1371/journal.pmed.1005035 (PMC13086301; doi:10.1371/journal.pmed.1005035)
Supplement: S2 Table — (DOCX) [file pmed.1005035.s005.docx]

| **ICD-10 codes** | **Description** |
| --- | --- |
| F11 | Mental and behavioural disorders due to use of opioids |
| F11.1 | Mental and behavioural disorders due to use of opioids Harmful use |
| F11.2 | Mental and behavioural disorders due to use of opioids Dependence syndrome |
| F11.3 | Mental and behavioural disorders due to use of opioids Withdrawal state |
| F11.4 | Mental and behavioural disorders due to use of opioids Withdrawal state with delirium |
| F11.5 | Mental and behavioural disorders due to use of opioids Psychotic disorder |
| F11.6 | Mental and behavioural disorders due to use of opioids Amnesic syndrome |
| F11.7 | Mental and behavioural disorders due to use of opioids Residual and late-onset psychotic disorder |
| F11.8 | Mental and behavioural disorders due to use of opioids Other mental and behavioural disorders |
| F11.9 | Mental and behavioural disorders due to use of opioids Unspecified mental and behavioural disorder |
| F12 | Mental and behavioural disorders due to use of cannabinoids |
| F12.1 | Mental and behavioural disorders due to use of cannabinoids Harmful use |
| F12.2 | Mental and behavioural disorders due to use of cannabinoids Dependence syndrome |
| F12.3 | Mental and behavioural disorders due to use of cannabinoids Withdrawal state |
| F12.4 | Mental and behavioural disorders due to use of cannabinoids Withdrawal state with delirium |
| F12.5 | Mental and behavioural disorders due to use of cannabinoids Psychotic disorder |
| F12.6 | Mental and behavioural disorders due to use of cannabinoids Amnesic syndrome |
| F12.7 | Mental and behavioural disorders due to use of cannabinoids Residual and late-onset psychotic disorder |
| F12.8 | Mental and behavioural disorders due to use of cannabinoids Other mental and behavioural disorders |
| F12.9 | Mental and behavioural disorders due to use of cannabinoids Unspecified mental and behavioural disorder |
| F13 | Mental and behavioural disorders due to use of sedatives or hypnotics |
| F13.1 | Mental and behavioural disorders due to use of sedatives or hypnotics Harmful use |
| F13.2 | Mental and behavioural disorders due to use of sedatives or hypnotics Dependence syndrome |
| F13.3 | Mental and behavioural disorders due to use of sedatives or hypnotics Withdrawal state |
| F13.4 | Mental and behavioural disorders due to use of sedatives or hypnotics Withdrawal state with delirium |
| F13.5 | Mental and behavioural disorders due to use of sedatives or hypnotics Psychotic disorder |
| F13.6 | Mental and behavioural disorders due to use of sedatives or hypnotics Amnesic syndrome |
| F13.7 | Mental and behavioural disorders due to use of sedatives or hypnotics Residual and late-onset psychotic disorder |
| F13.8 | Mental and behavioural disorders due to use of sedatives or hypnotics Other mental and behavioural disorders |
| F13.9 | Mental and behavioural disorders due to use of sedatives or hypnotics Unspecified mental and behavioural disorder |
| F14 | Mental and behavioural disorders due to use of cocaine |
| F14.1 | Mental and behavioural disorders due to use of cocaine Harmful use |
| F14.2 | Mental and behavioural disorders due to use of cocaine Dependence syndrome |
| F14.3 | Mental and behavioural disorders due to use of cocaine Withdrawal state |
| F14.4 | Mental and behavioural disorders due to use of cocaine Withdrawal state with delirium |
| F14.5 | Mental and behavioural disorders due to use of cocaine Psychotic disorder |
| F14.6 | Mental and behavioural disorders due to use of cocaine Amnesic syndrome |
| F14.7 | Mental and behavioural disorders due to use of cocaine Residual and late-onset psychotic disorder |
| F14.8 | Mental and behavioural disorders due to use of cocaine Other mental and behavioural disorders |
| F14.9 | Mental and behavioural disorders due to use of cocaine Unspecified mental and behavioural disorder |
| F15 | Mental and behavioural disorders due to use of other stimulants, including caffeine |
| F15.0 | Mental and behavioural disorders due to use of other stimulants, including caffeine Acute intoxication |
| F15.1 | Mental and behavioural disorders due to use of other stimulants, including caffeine Harmful use |
| F15.2 | Mental and behavioural disorders due to use of other stimulants, including caffeine Dependence syndrome |
| F15.3 | Mental and behavioural disorders due to use of other stimulants, including caffeine Withdrawal state |
| F15.4 | Mental and behavioural disorders due to use of other stimulants, including caffeine Withdrawal state with delirium |
| F15.5 | Mental and behavioural disorders due to use of other stimulants, including caffeine Psychotic disorder |
| F15.6 | Mental and behavioural disorders due to use of other stimulants, including caffeine Amnesic syndrome |
| F15.7 | Mental and behavioural disorders due to use of other stimulants, including caffeine Residual and late-onset psychotic disorder |
| F15.8 | Mental and behavioural disorders due to use of other stimulants, including caffeine Other mental and behavioural disorders |
| F15.9 | Mental and behavioural disorders due to use of other stimulants, including caffeine Unspecified mental and behavioural disorder |
| F16 | Mental and behavioural disorders due to use of hallucinogens |
| F16.0 | Mental and behavioural disorders due to use of hallucinogens Acute intoxication |
| F16.1 | Mental and behavioural disorders due to use of hallucinogens Harmful use |
| F16.2 | Mental and behavioural disorders due to use of hallucinogens Dependence syndrome |
| F16.3 | Mental and behavioural disorders due to use of hallucinogens Withdrawal state |
| F16.4 | Mental and behavioural disorders due to use of hallucinogens Withdrawal state with delirium |
| F16.5 | Mental and behavioural disorders due to use of hallucinogens Psychotic disorder |
| F16.6 | Mental and behavioural disorders due to use of hallucinogens Amnesic syndrome |
| F16.7 | Mental and behavioural disorders due to use of hallucinogens Residual and late-onset psychotic disorder |
| F16.8 | Mental and behavioural disorders due to use of hallucinogens Other mental and behavioural disorders |
| F16.9 | Mental and behavioural disorders due to use of hallucinogens Unspecified mental and behavioural disorder |
| F18 | Mental and behavioural disorders due to use of volatile solvents |
| F18.0 | Mental and behavioural disorders due to use of volatile solvents Acute intoxication |
| F18.1 | Mental and behavioural disorders due to use of volatile solvents Harmful use |
| F18.2 | Mental and behavioural disorders due to use of volatile solvents Dependence syndrome |
| F18.3 | Mental and behavioural disorders due to use of volatile solvents Withdrawal state |
| F18.4 | Mental and behavioural disorders due to use of volatile solvents Withdrawal state with delirium |
| F18.5 | Mental and behavioural disorders due to use of volatile solvents Psychotic disorder |
| F18.6 | Mental and behavioural disorders due to use of volatile solvents Amnesic syndrome |
| F18.7 | Mental and behavioural disorders due to use of volatile solvents Residual and late-onset psychotic disorder |
| F18.8 | Mental and behavioural disorders due to use of volatile solvents Other mental and behavioural disorders |
| F18.9 | Mental and behavioural disorders due to use of volatile solvents Unspecified mental and behavioural disorder |
| F19 | Mental and behavioural disorders due to multiple drug use and use of other psychoactive substances |
| F19.0 | Mental and behavioural disorders due to multiple drug use and use of other psychoactive substances Acute intoxication |
| F19.1 | Mental and behavioural disorders due to multiple drug use and use of other psychoactive substances Harmful use |
| F19.2 | Mental and behavioural disorders due to multiple drug use and use of other psychoactive substances Dependence syndrome |
| F19.3 | Mental and behavioural disorders due to multiple drug use and use of other psychoactive substances Withdrawal state |
| F19.4 | Mental and behavioural disorders due to multiple drug use and use of other psychoactive substances Withdrawal state with delirium |
| F19.5 | Mental and behavioural disorders due to multiple drug use and use of other psychoactive substances Psychotic disorder |
| F19.6 | Mental and behavioural disorders due to multiple drug use and use of other psychoactive substances Amnesic syndrome |
| F19.7 | Mental and behavioural disorders due to multiple drug use and use of other psychoactive substances Residual and late-onset psychotic disorder |
| F19.8 | Mental and behavioural disorders due to multiple drug use and use of other psychoactive substances Other mental and behavioural disorders |
| F19.9 | Mental and behavioural disorders due to multiple drug use and use of other psychoactive substances Unspecified mental and behavioural disorder |
| T36 | Poisoning by systemic antibiotics |
| T36.0 | Poisoning: Penicillins |
| T36.1 | Poisoning: Cefalosporins and other beta-lactam antibiotics |
| T36.2 | Poisoning: Chloramphenicol group |
| T36.3 | Poisoning: Macrolides |
| T36.4 | Poisoning: Tetracyclines |
| T36.5 | Poisoning: Aminoglycosides |
| T36.6 | Poisoning: Rifamycins |
| T36.7 | Poisoning: Antifungal antibiotics, systemically used |
| T36.8 | Poisoning: Other systemic antibiotics |
| T36.9 | Poisoning: Systemic antibiotic, unspecified |
| T37 | Poisoning by other systemic anti-infectives and antiparasitics |
| T37.0 | Poisoning: Sulfonamides |
| T37.1 | Poisoning: Antimycobacterial drugs |
| T37.2 | Poisoning: Antimalarials and drugs acting on other blood protozoa |
| T37.3 | Poisoning: Other antiprotozoal drugs |
| T37.4 | Poisoning: Anthelminthics |
| T37.5 | Poisoning: Antiviral drugs |
| T37.8 | Poisoning: Other specified systemic anti-infectives and antiparasitics |
| T37.9 | Poisoning: Systemic anti-infective and antiparasitic, unspecified |
| T38 | Poisoning by hormones and their synthetic substitutes and antagonists, not elsewhere classified |
| T38.0 | Poisoning: Glucocorticoids and synthetic analogues |
| T38.1 | Poisoning: Thyroid hormones and substitutes |
| T38.2 | Poisoning: Antithyroid drugs |
| T38.3 | Poisoning: Insulin and oral hypoglycaemic [antidiabetic] drugs |
| T38.4 | Poisoning: Oral contraceptives |
| T38.5 | Poisoning: Other estrogens and progestogens |
| T38.6 | Poisoning: Antigonadotrophins, antiestrogens, antiandrogens, not elsewhere classified |
| T38.7 | Poisoning: Androgens and anabolic congeners |
| T38.8 | Poisoning: Other and unspecified hormones and their synthetic substitutes |
| T38.9 | Poisoning: Other and unspecified hormone antagonists |
| T39 | Poisoning by nonopioid analgesics, antipyretics and antirheumatics |
| T39.0 | Poisoning: Salicylates |
| T39.1 | Poisoning: 4-Aminophenol derivatives |
| T39.2 | Poisoning: Pyrazolone derivatives |
| T39.3 | Poisoning: Other nonsteroidal anti-inflammatory drugs [NSAID] |
| T39.4 | Poisoning: Antirheumatics, not elsewhere classified |
| T39.8 | Poisoning: Other nonopioid analgesics and antipyretics, not elsewhere classified |
| T39.9 | Poisoning: Nonopioid analgesic, antipyretic and antirheumatic, unspecified |
| T40 | Poisoning by narcotics and psychodysleptics [hallucinogens] |
| T40.0 | Poisoning: Opium |
| T40.1 | Poisoning: Heroin |
| T40.2 | Poisoning: Other opioids |
| T40.3 | Poisoning: Methadone |
| T40.4 | Poisoning: Other synthetic narcotics |
| T40.5 | Poisoning: Cocaine |
| T40.6 | Poisoning: Other and unspecified narcotics |
| T40.7 | Poisoning: Cannabis (derivatives) |
| T40.8 | Poisoning: Lysergide [LSD] |
| T40.9 | Poisoning: Other and unspecified psychodysleptics [hallucinogens] |
| T41 | Poisoning by anaesthetics and therapeutic gases |
| T41.0 | Poisoning: Inhaled anaesthetics |
| T41.1 | Poisoning: Intravenous anaesthetics |
| T41.2 | Poisoning: Other and unspecified general anaesthetics |
| T41.3 | Poisoning: Local anaesthetics |
| T41.4 | Poisoning: Anaesthetic, unspecified |
| T41.5 | Poisoning: Therapeutic gases |
| T42 | Poisoning by antiepileptic, sedative-hypnotic and antiparkinsonism drugs |
| T42.0 | Poisoning: Hydantoin derivatives |
| T42.1 | Poisoning: Iminostilbenes |
| T42.2 | Poisoning: Succinimides and oxazolidinediones |
| T42.3 | Poisoning: Barbiturates |
| T42.4 | Poisoning: Benzodiazepines |
| T42.5 | Poisoning: Mixed antiepileptics, not elsewhere classified |
| T42.6 | Poisoning: Other antiepileptic and sedative-hypnotic drugs |
| T42.7 | Poisoning: Antiepileptic and sedative-hypnotic drugs, unspecified |
| T42.8 | Poisoning: Antiparkinsonism drugs and other central muscle-tone depressants |
| T43 | Poisoning by psychotropic drugs, not elsewhere classified |
| T43.0 | Poisoning: Tricyclic and tetracyclic antidepressants |
| T43.1 | Poisoning: Monoamine-oxidase-inhibitor antidepressants |
| T43.2 | Poisoning: Other and unspecified antidepressants |
| T43.3 | Poisoning: Phenothiazine antipsychotics and neuroleptics |
| T43.4 | Poisoning: Butyrophenone and thioxanthene neuroleptics |
| T43.5 | Poisoning: Other and unspecified antipsychotics and neuroleptics |
| T43.6 | Poisoning: Psychostimulants with abuse potential |
| T43.8 | Poisoning: Other psychotropic drugs, not elsewhere classified |
| T43.9 | Poisoning: Psychotropic drug, unspecified |
| T44 | Poisoning by drugs primarily affecting the autonomic nervous system |
| T44.0 | Poisoning: Anticholinesterase agents |
| T44.1 | Poisoning: Other parasympathomimetics [cholinergics] |
| T44.2 | Poisoning: Ganglionic blocking drugs, not elsewhere classified |
| T44.3 | Poisoning: Other parasympatholytics [anticholinergics and antimuscarinics] and spasmolytics, not elsewhere classified |
| T44.4 | Poisoning: Predominantly alpha-adrenoreceptor agonists, not elsewhere classified |
| T44.5 | Poisoning: Predominantly beta-adrenoreceptor agonists, not elsewhere classified |
| T44.6 | Poisoning: Alpha-adrenoreceptor antagonists, not elsewhere classified |
| T44.7 | Poisoning: Beta-adrenoreceptor antagonists, not elsewhere classified |
| T44.8 | Poisoning: Centrally acting and adrenergic-neuron-blocking agents, not elsewhere classified |
| T44.9 | Poisoning: Other and unspecified drugs primarily affecting the autonomic nervous system |
| T45 | Poisoning by primarily systemic and haematological agents, not elsewhere classified |
| T45.0 | Poisoning: Antiallergic and antiemetic drugs |
| T45.1 | Poisoning: Antineoplastic and immunosuppressive drugs |
| T45.2 | Poisoning: Vitamins, not elsewhere classified |
| T45.3 | Poisoning: Enzymes, not elsewhere classified |
| T45.4 | Poisoning: Iron and its compounds |
| T45.5 | Poisoning: Anticoagulants |
| T45.6 | Poisoning: Fibrinolysis-affecting drugs |
| T45.7 | Poisoning: Anticoagulant antagonists, vitamin K and other coagulants |
| T45.8 | Poisoning: Other primarily systemic and haematological agents |
| T45.9 | Poisoning: Primarily systemic and haematological agent, unspecified |
| T46 | Poisoning by agents primarily affecting the cardiovascular system |
| T46.0 | Poisoning: Cardiac-stimulant glycosides and drugs of similar action |
| T46.1 | Poisoning: Calcium-channel blockers |
| T46.2 | Poisoning: Other antidysrhythmic drugs, not elsewhere classified |
| T46.3 | Poisoning: Coronary vasodilators, not elsewhere classified |
| T46.4 | Poisoning: Angiotensin-converting-enzyme inhibitors |
| T46.5 | Poisoning: Other antihypertensive drugs, not elsewhere classified |
| T46.6 | Poisoning: Antihyperlipidaemic and antiarteriosclerotic drugs |
| T46.7 | Poisoning: Peripheral vasodilators |
| T46.8 | Poisoning: Antivaricose drugs, including sclerosing agents |
| T46.9 | Poisoning: Other and unspecified agents primarily affecting the cardiovascular system |
| T47 | Poisoning by agents primarily affecting the gastrointestinal system |
| T47.0 | -receptor antagonists |
| T47.1 | Poisoning: Other antacids and anti-gastric-secretion drugs |
| T47.2 | Poisoning: Stimulant laxatives |
| T47.3 | Poisoning: Saline and osmotic laxatives |
| T47.4 | Poisoning: Other laxatives |
| T47.5 | Poisoning: Digestants |
| T47.6 | Poisoning: Antidiarrhoeal drugs |
| T47.7 | Poisoning: Emetics |
| T47.8 | Poisoning: Other agents primarily affecting the gastrointestinal system |
| T47.9 | Poisoning: Agent primarily affecting the gastrointestinal system, unspecified |
| T48 | Poisoning by agents primarily acting on smooth and skeletal muscles and the respiratory system |
| T48.0 | Poisoning: Oxytocic drugs |
| T48.1 | Poisoning: Skeletal muscle relaxants [neuromuscular blocking agents] |
| T48.2 | Poisoning: Other and unspecified agents primarily acting on muscles |
| T48.3 | Poisoning: Antitussives |
| T48.4 | Poisoning: Expectorants |
| T48.5 | Poisoning: Anti-common-cold drugs |
| T48.6 | Poisoning: Antiasthmatics, not elsewhere classified |
| T48.7 | Poisoning: Other and unspecified agents primarily acting on the respiratory system |
| T49 | Poisoning by topical agents primarily affecting skin and mucous membrane and by ophthalmological, otorhinolaryngological and dental drugs |
| T49.0 | Poisoning: Local antifungal, anti-infective and anti-inflammatory drugs, not elsewhere classified |
| T49.1 | Poisoning: Antipruritics |
| T49.2 | Poisoning: Local astringents and local detergents |
| T49.3 | Poisoning: Emollients, demulcents and protectants |
| T49.4 | Poisoning: Keratolytics, keratoplastics and other hair treatment drugs and preparations |
| T49.5 | Poisoning: Ophthalmological drugs and preparations |
| T49.6 | Poisoning: Otorhinolaryngological drugs and preparations |
| T49.7 | Poisoning: Dental drugs, topically applied |
| T49.8 | Poisoning: Other topical agents |
| T49.9 | Poisoning: Topical agent, unspecified |
| T50 | Poisoning by diuretics and other and unspecified drugs, medicaments and biological substances |
| T50.0 | Poisoning: Mineralocorticoids and their antagonists |
| T50.1 | Poisoning: Loop [high-ceiling] diuretics |
| T50.2 | Poisoning: Carbonic-anhydrase inhibitors, benzothiadiazides and other diuretics |
| T50.3 | Poisoning: Electrolytic, caloric and water-balance agents |
| T50.4 | Poisoning: Drugs affecting uric acid metabolism |
| T50.5 | Poisoning: Appetite depressants |
| T50.6 | Poisoning: Antidotes and chelating agents, not elsewhere classified |
| T50.7 | Poisoning: Analeptics and opioid receptor antagonists |
| T50.8 | Poisoning: Diagnostic agents |
| T50.9 | Poisoning: Other and unspecified drugs, medicaments and biological substances |
| X40 | Accidental poisoning by and exposure to nonopioid analgesics, antipyretics and antirheumatics |
| X40.0 | Accidental poisoning by and exposure to nonopioid analgesics, antipyretics and antirheumatics Home |
| X40.1 | Accidental poisoning by and exposure to nonopioid analgesics, antipyretics and antirheumatics Residential institution |
| X40.2 | Accidental poisoning by and exposure to nonopioid analgesics, antipyretics and antirheumatics School, other institution and public administrative area |
| X40.3 | Accidental poisoning by and exposure to nonopioid analgesics, antipyretics and antirheumatics Sports and athletics area |
| X40.4 | Accidental poisoning by and exposure to nonopioid analgesics, antipyretics and antirheumatics Street and highway |
| X40.5 | Accidental poisoning by and exposure to nonopioid analgesics, antipyretics and antirheumatics Trade and service area |
| X40.6 | Accidental poisoning by and exposure to nonopioid analgesics, antipyretics and antirheumatics Industrial and construction area |
| X40.7 | Accidental poisoning by and exposure to nonopioid analgesics, antipyretics and antirheumatics Farm |
| X40.8 | Accidental poisoning by and exposure to nonopioid analgesics, antipyretics and antirheumatics Other specified places |
| X40.9 | Accidental poisoning by and exposure to nonopioid analgesics, antipyretics and antirheumatics Unspecified place |
| X41 | Accidental poisoning by and exposure to antiepileptic, sedative-hypnotic, antiparkinsonism and psychotropic drugs, not elsewhere classified |
| X41.0 | Accidental poisoning by and exposure to antiepileptic, sedative-hypnotic, antiparkinsonism and psychotropic drugs, not elsewhere classified Home |
| X41.1 | Accidental poisoning by and exposure to antiepileptic, sedative-hypnotic, antiparkinsonism and psychotropic drugs, not elsewhere classified Residential institution |
| X41.2 | Accidental poisoning by and exposure to antiepileptic, sedative-hypnotic, antiparkinsonism and psychotropic drugs, not elsewhere classified School, other institution and public administrative area |
| X41.3 | Accidental poisoning by and exposure to antiepileptic, sedative-hypnotic, antiparkinsonism and psychotropic drugs, not elsewhere classified Sports and athletics area |
| X41.4 | Accidental poisoning by and exposure to antiepileptic, sedative-hypnotic, antiparkinsonism and psychotropic drugs, not elsewhere classified Street and highway |
| X41.5 | Accidental poisoning by and exposure to antiepileptic, sedative-hypnotic, antiparkinsonism and psychotropic drugs, not elsewhere classified Trade and service area |
| X41.6 | Accidental poisoning by and exposure to antiepileptic, sedative-hypnotic, antiparkinsonism and psychotropic drugs, not elsewhere classified Industrial and construction area |
| X41.7 | Accidental poisoning by and exposure to antiepileptic, sedative-hypnotic, antiparkinsonism and psychotropic drugs, not elsewhere classified Farm |
| X41.8 | Accidental poisoning by and exposure to antiepileptic, sedative-hypnotic, antiparkinsonism and psychotropic drugs, not elsewhere classified Other specified places |
| X41.9 | Accidental poisoning by and exposure to antiepileptic, sedative-hypnotic, antiparkinsonism and psychotropic drugs, not elsewhere classified Unspecified place |
| X42 | Accidental poisoning by and exposure to narcotics and psychodysleptics [hallucinogens], not elsewhere classified |
| X42.0 | Accidental poisoning by and exposure to narcotics and psychodysleptics [hallucinogens], not elsewhere classified Home |
| X42.1 | Accidental poisoning by and exposure to narcotics and psychodysleptics [hallucinogens], not elsewhere classified Residential institution |
| X42.2 | Accidental poisoning by and exposure to narcotics and psychodysleptics [hallucinogens], not elsewhere classified School, other institution and public administrative area |
| X42.3 | Accidental poisoning by and exposure to narcotics and psychodysleptics [hallucinogens], not elsewhere classified Sports and athletics area |
| X42.4 | Accidental poisoning by and exposure to narcotics and psychodysleptics [hallucinogens], not elsewhere classified Street and highway |
| X42.5 | Accidental poisoning by and exposure to narcotics and psychodysleptics [hallucinogens], not elsewhere classified Trade and service area |
| X42.6 | Accidental poisoning by and exposure to narcotics and psychodysleptics [hallucinogens], not elsewhere classified Industrial and construction area |
| X42.7 | Accidental poisoning by and exposure to narcotics and psychodysleptics [hallucinogens], not elsewhere classified Farm |
| X42.8 | Accidental poisoning by and exposure to narcotics and psychodysleptics [hallucinogens], not elsewhere classified Other specified places |
| X42.9 | Accidental poisoning by and exposure to narcotics and psychodysleptics [hallucinogens], not elsewhere classified Unspecified place |
| X43 | Accidental poisoning by and exposure to other drugs acting on the autonomic nervous system |
| X43.0 | Accidental poisoning by and exposure to other drugs acting on the autonomic nervous system Home |
| X43.1 | Accidental poisoning by and exposure to other drugs acting on the autonomic nervous system Residential institution |
| X43.2 | Accidental poisoning by and exposure to other drugs acting on the autonomic nervous system School, other institution and public administrative area |
| X43.3 | Accidental poisoning by and exposure to other drugs acting on the autonomic nervous system Sports and athletics area |
| X43.4 | Accidental poisoning by and exposure to other drugs acting on the autonomic nervous system Street and highway |
| X43.5 | Accidental poisoning by and exposure to other drugs acting on the autonomic nervous system Trade and service area |
| X43.6 | Accidental poisoning by and exposure to other drugs acting on the autonomic nervous system Industrial and construction area |
| X43.7 | Accidental poisoning by and exposure to other drugs acting on the autonomic nervous system Farm |
| X43.8 | Accidental poisoning by and exposure to other drugs acting on the autonomic nervous system Other specified places |
| X43.9 | Accidental poisoning by and exposure to other drugs acting on the autonomic nervous system Unspecified place |
| X44 | Accidental poisoning by and exposure to other and unspecified drugs, medicaments and biological substances |
| X44.0 | Accidental poisoning by and exposure to other and unspecified drugs, medicaments and biological substances Home |
| X44.1 | Accidental poisoning by and exposure to other and unspecified drugs, medicaments and biological substances Residential institution |
| X44.2 | Accidental poisoning by and exposure to other and unspecified drugs, medicaments and biological substances School, other institution and public administrative area |
| X44.3 | Accidental poisoning by and exposure to other and unspecified drugs, medicaments and biological substances Sports and athletics area |
| X44.4 | Accidental poisoning by and exposure to other and unspecified drugs, medicaments and biological substances Street and highway |
| X44.5 | Accidental poisoning by and exposure to other and unspecified drugs, medicaments and biological substances Trade and service area |
| X44.6 | Accidental poisoning by and exposure to other and unspecified drugs, medicaments and biological substances Industrial and construction area |
| X44.7 | Accidental poisoning by and exposure to other and unspecified drugs, medicaments and biological substances Farm |
| X44.8 | Accidental poisoning by and exposure to other and unspecified drugs, medicaments and biological substances Other specified places |
| X44.9 | Accidental poisoning by and exposure to other and unspecified drugs, medicaments and biological substances Unspecified place |
| X60 | Intentional self-poisoning by and exposure to nonopioid analgesics, antipyretics and antirheumatics |
| X61 | Intentional self-poisoning by and exposure to antiepileptic, sedative-hypnotic, antiparkinsonism and psychotropic drugs, not elsewhere classified |
| X62 | Intentional self-poisoning by and exposure to narcotics and psychodysleptics [hallucinogens], not elsewhere classified |
| X63 | Intentional self-poisoning by and exposure to other drugs acting on the autonomic nervous system |
| X64 | Intentional self-poisoning by and exposure to other and unspecified drugs, medicaments and biological substances |
| X85 | Assault by drugs, medicaments and biological substances |
| Y10 | Poisoning by and exposure to nonopioid analgesics, antipyretics and antirheumatics, undetermined intent |
| Y11 | Poisoning by and exposure to antiepileptic, sedative-hypnotic, antiparkinsonism and psychotropic drugs, not elsewhere classified, undetermined intent |
| Y12 | Poisoning by and exposure to narcotics and psychodysleptics [hallucinogens], not elsewhere classified, undetermined intent |
| Y13 | Poisoning by and exposure to other drugs acting on the autonomic nervous system, undetermined intent |
| Y14 | Poisoning by and exposure to other and unspecified drugs, medicaments and biological substances, undetermined intent |

ICD-10 = International Statistical Classification of Diseases and Related Health Problems 10^th^ Revision
