# Supplementary material for: Association between gabapentinoid treatment, concurrent use with opioid or benzodiazepine and the risk of drug poisoning: A self-controlled case series study
Source: PLoS Med. 2026 Apr 16;23(4):e1005035. doi: 10.1371/journal.pmed.1005035 (PMC13086301; doi:10.1371/journal.pmed.1005035)
Supplement: S3 Table — (DOCX) [file pmed.1005035.s006.docx]

| **ATC code** | **Name of Drug** |
| --- | --- |
| N03AX23 | Brivaracetam |
| N03AX24 | Cannabidiol |
| N03AF01 | Carbamazepine |
| N03AX25 | Cenobamate |
| N03AF04 | Eslicarbazepine acetate |
| N03AD01 | Ethosuximide |
| N03AX10 | Felbamate |
| N03AX18 | Lacosamide |
| N03AX09 | Lamotrigine |
| N03AX14 | Levetiracetam |
| N03AD03 | Mesuximide |
| N03AF02 | Oxcarbazepine |
| N03AX22 | Perampanel |
| N03AA02 | Phenobarbital |
| N03AB02 | Phenytoin |
| N03AA03 | Primidone |
| N03AX21 | Retigabine |
| N03AF03 | Rufinamide |
| N03AG01 | Sodium Valproate |
| N03AX17 | Stiripentol |
| N03AX03 | Sultiame |
| N03AG06 | Tiagabine |
| N03AX11 | Topiramate |
| N03AG04 | Vigabatrin |
| N03AX15 | Zonisamide |
| N03AE01 | Clonazepam |

ATC = Anatomical Therapeutic Chemical
