# Supplementary material for: Association between gabapentinoid treatment, concurrent use with opioid or benzodiazepine and the risk of drug poisoning: A self-controlled case series study
Source: PLoS Med. 2026 Apr 16;23(4):e1005035. doi: 10.1371/journal.pmed.1005035 (PMC13086301; doi:10.1371/journal.pmed.1005035)
Supplement: S4 Table — (DOCX) [file pmed.1005035.s007.docx]

| **ATC code** | **Name of Drug** |
| --- | --- |
| N02AA02 | Opium |
| N02AJ01 | Co-dydramol |
| N02AJ06 | Co-codamol |
| N02AJ07 | Co-codaprin |
| N02AE01 | Buprenorphine |
| R05DA04 | Codeine |
| N02AC01 | Dextromoramide |
| N02AC04 | Dextropropoxyphene |
| N07BC06 | Diamorphine |
| N02AA08 | Dihydrocodeine |
| N02AB03 | Fentanyl |
| N02AA03 | Hydromorphone |
| N02AX05 | Meptazinol |
| N07BC02 | Methadone |
| N02AA01 | Morphine |
| N02AF02 | Nalbuphine |
| N02AA05 | Oxycodone |
| N02AA55 | Oxycodone and Naloxone |
| N02AA10 | Papaveretum |
| N02AD01 | Pentazocine |
| N02AB02 | Pethidine |
| N02AX06 | Tapentadol |
| N02AX02 | Tramadol |
| N02AJ14 | Tramadol and Dexketoprofen |

ATC = Anatomical Therapeutic Chemical
