# Supplementary material for: Association between gabapentinoid treatment, concurrent use with opioid or benzodiazepine and the risk of drug poisoning: A self-controlled case series study
Source: PLoS Med. 2026 Apr 16;23(4):e1005035. doi: 10.1371/journal.pmed.1005035 (PMC13086301; doi:10.1371/journal.pmed.1005035)
Supplement: S5 Table — (DOCX) [file pmed.1005035.s008.docx]

| **ATC code** | **Name of Drug** |
| --- | --- |
| N05CC01 | Chloral Hydrate |
| N05CM02 | Clomethiazole |
| N05CC01 | Cloral Betaine |
| N05CH01 | Melatonin |
| N05CM07 | Triclofos |
| N05CM18 | Dexmedetomidine |
| N05CF03 | Zaleplon |
| N05CF02 | Zolpidem |
| N05CF01 | Zopiclone |
| N05BE01 | Buspirone |
| N05BC01 | Meprobamate |
| N05CA02 | Amobarbital |
| N05CA03 | Butobarbital |
| N05CA06 | Secobarbital |
| N05CA19 | Thiopental |

ATC = Anatomical Therapeutic Chemical
