# Supplementary material for: Association between gabapentinoid treatment, concurrent use with opioid or benzodiazepine and the risk of drug poisoning: A self-controlled case series study
Source: PLoS Med. 2026 Apr 16;23(4):e1005035. doi: 10.1371/journal.pmed.1005035 (PMC13086301; doi:10.1371/journal.pmed.1005035)
Supplement: S6 Table — (DOCX) [file pmed.1005035.s009.docx]

| **ATC code** | **Name of Drug** |
| --- | --- |
| N05CD01 | Flurazepam |
| N05CD11 | Loprazolam |
| N05CD06 | Lormetazepam |
| N05CD08 | Midazolam |
| N05CD02 | Nitrazepam |
| N05CD07 | Temazepam |
| N05BA08 | Bromazepam |
| N05BA02 | Chlordiazepoxide |
| N05BA01 | Diazepam |
| N05BA06 | Lorazepam |
| N05BA04 | Oxazepam |
| N05BA09 | Clobazam |

ATC = Anatomical Therapeutic Chemical
