# Supplementary material for: Association between gabapentinoid treatment, concurrent use with opioid or benzodiazepine and the risk of drug poisoning: A self-controlled case series study
Source: PLoS Med. 2026 Apr 16;23(4):e1005035. doi: 10.1371/journal.pmed.1005035 (PMC13086301; doi:10.1371/journal.pmed.1005035)
Supplement: S7 Table — (DOCX) [file pmed.1005035.s010.docx]

| **ATC code** | **Name of Drug** |
| --- | --- |
| N06AA09 | Amitriptyline |
| N06AA17 | Amoxapine |
| N06AA04 | Clomipramine |
| N06AA16 | Dosulepin |
| D04AX01 | Doxepin |
| N06AA02 | Imipramine |
| N06AA07 | Lofepramine |
| N06AA21 | Maprotiline |
| N06AX03 | Mianserin |
| N06AA10 | Nortriptyline |
| N06AX05 | Trazodone |
| N06AA06 | Trimipramine |
| N06AF01 | Isocarboxazid |
| N06AG02 | Moclobemide |
| N06AF03 | Phenelzine |
| N06AF04 | Tranylcypromine |
| N06AB04 | Citalopram |
| N06AX21 | Duloxetine |
| N06AB10 | Escitalopram |
| N06AB03 | Fluoxetine |
| N06AB08 | Fluvoxamine |
| N06AB05 | Paroxetine |
| N06AB06 | Sertraline |
| N06AX22 | Agomelatine |
| N06AX11 | Mirtazapine |
| N06AX06 | Nefazodone |
| N06AX01 | Oxitriptan |
| N06AX18 | Reboxetine |
| N06AX02 | Tryptophan |
| N06AX16 | Venlafaxine |
| N06AX26 | Vortioxetine |
| N06AX12 | Bupropion |
| N06AX27 | Esketamine |

ATC = Anatomical Therapeutic Chemical
