# Supplementary material for: Association between gabapentinoid treatment, concurrent use with opioid or benzodiazepine and the risk of drug poisoning: A self-controlled case series study
Source: PLoS Med. 2026 Apr 16;23(4):e1005035. doi: 10.1371/journal.pmed.1005035 (PMC13086301; doi:10.1371/journal.pmed.1005035)
Supplement: S8 Table — (DOCX) [file pmed.1005035.s011.docx]

| **ATC code** | **Name of Drug** |
| --- | --- |
| N05AL05 | Amisulpride |
| N05AX12 | Aripiprazole |
| N05AD07 | Benperidol |
| N05AX15 | Cariprazine |
| N05AA01 | Chlorpromazine |
| N05AF03 | Chlorprothixene |
| N05AH02 | Clozapine |
| N05AF01 | Flupentixol |
| N05AB02 | Fluphenazine |
| N05AD01 | Haloperidol |
| N05AA02 | Levomepromazine |
| N05AH01 | Loxapine |
| N05AE05 | Lurasidone |
| N05AD03 | Melperone |
| N05AH03 | Olanzapine |
| N05AX13 | Paliperidone |
| N05AC01 | Pericyazine |
| N05AB03 | Perphenazine |
| N05AG02 | Pimozide |
| N05AA03 | Promazine |
| N05AH04 | Quetiapine |
| N05AX08 | Risperidone |
| N05AL01 | Sulpiride |
| N05AC02 | Thioridazine |
| N05AB06 | Trifluoperazine |
| N05AE04 | Ziprasidone |
| N05AX11 | Zotepine |
| N05AF05 | Zuclopenthixol |
| N05AD08 | Droperidol |
| N05AC04 | Pipotiazine |
| N05AH05 | Asenapine |
| N05AB04 | Prochlorperazine |

ATC = Anatomical Therapeutic Chemical
