# Supplementary material for: Association between gabapentinoid treatment, concurrent use with opioid or benzodiazepine and the risk of drug poisoning: A self-controlled case series study
Source: PLoS Med. 2026 Apr 16;23(4):e1005035. doi: 10.1371/journal.pmed.1005035 (PMC13086301; doi:10.1371/journal.pmed.1005035)
Supplement: S9 Table — (DOCX) [file pmed.1005035.s012.docx]

| **ATC code** | **Name of Drug** |
| --- | --- |
| N05AN01 | Lithium |

ATC = Anatomical Therapeutic Chemical
