# Supplementary material for: Association between gabapentinoid treatment, concurrent use with opioid or benzodiazepine and the risk of drug poisoning: A self-controlled case series study
Source: PLoS Med. 2026 Apr 16;23(4):e1005035. doi: 10.1371/journal.pmed.1005035 (PMC13086301; doi:10.1371/journal.pmed.1005035)
Supplement: S10 Table — (DOCX) [file pmed.1005035.s013.docx]

| **ATC code** | **Name of Drug** |
| --- | --- |
| M01AB16 | Aceclofenac |
| M01AB11 | Acemetacin |
| M01AX04 | Azapropazone |
| M01AH01 | Celecoxib |
| M01AE14 | Dexibuprofen |
| M01AE17 | Dexketoprofen |
| M01AB05 | Diclofenac sodium/ potassium |
| M01AB08 | Etodolac |
| M01AH05 | Etoricoxib |
| M01AE05 | Fenbufen |
| M01AE04 | Fenoprofen |
| M01AE09 | Flurbiprofen |
| M01AE01 | Ibuprofen |
| M01AE51 | Ibuprofen lysine |
| M01AB01 | Indometacin |
| M01AE03 | Ketoprofen |
| M01AC05 | Lornoxicam |
| M01AH06 | Lumiracoxib |
| M01AG01 | Mefenamic acid |
| M01AC06 | Meloxicam |
| M01AX01 | Nabumetone |
| M01AE02 | Naproxen |
| M01AA01 | Phenylbutazone |
| M01AC01 | Piroxicam |
| M01AH02 | Rofecoxib |
| M01AB02 | Sulindac |
| M01AC02 | Tenoxicam |
| M01AE11 | Tiaprofenic acid |
| N02BA11 | Diflunisal |

ATC = Anatomical Therapeutic Chemical
