# Supplementary material for: Association between gabapentinoid treatment, concurrent use with opioid or benzodiazepine and the risk of drug poisoning: A self-controlled case series study
Source: PLoS Med. 2026 Apr 16;23(4):e1005035. doi: 10.1371/journal.pmed.1005035 (PMC13086301; doi:10.1371/journal.pmed.1005035)
Supplement: S11 Table — (DOCX) [file pmed.1005035.s014.docx]

| **ICD-10 codes** | **Description** |
| --- | --- |
| A05 | Other bacterial foodborne intoxications, not elsewhere classified |
| A05.0 | Foodborne staphylococcal intoxication |
| A05.1 | Botulism |
| A05.2 | Foodborne Clostridium perfringens [Clostridium welchii] intoxication |
| A05.3 | Foodborne Vibrio parahaemolyticus intoxication |
| A05.4 | Foodborne Bacillus cereus intoxication |
| A05.8 | Other specified bacterial foodborne intoxications |
| A05.9 | Bacterial foodborne intoxication, unspecified |
| T61 | Toxic effect of noxious substances eaten as seafood |
| T61.0 | Toxic effect: Ciguatera fish poisoning |
| T61.1 | Toxic effect: Scombroid fish poisoning |
| T61.2 | Toxic effect: Other fish and shellfish poisoning |
| T61.8 | Toxic effect: Toxic effect of other seafoods |
| T61.9 | Toxic effect: Toxic effect of unspecified seafood |
| T62 | Toxic effect of other noxious substances eaten as food |
| T62.0 | Toxic effect: Ingested mushrooms |
| T62.1 | Toxic effect: Ingested berries |
| T62.2 | Toxic effect: Other ingested (parts of) plant(s) |
| T62.8 | Toxic effect: Other specified noxious substances eaten as food |
| T62.9 | Toxic effect: Noxious substance eaten as food, unspecified |

ICD-10 = International Statistical Classification of Diseases and Related Health Problems 10^th^ Revision
