# Supplementary material for: Association between gabapentinoid treatment, concurrent use with opioid or benzodiazepine and the risk of drug poisoning: A self-controlled case series study
Source: PLoS Med. 2026 Apr 16;23(4):e1005035. doi: 10.1371/journal.pmed.1005035 (PMC13086301; doi:10.1371/journal.pmed.1005035)
Supplement: S12 Table — (DOCX) [file pmed.1005035.s015.docx]

|  | **No. of Individuals (%)** | **Mean age (SD) at observation start (years)** | **Median length of prescriptions (****IQR) (days)** | **Treatment periods** | | **Non-treatment periods** | |
| --- | --- | --- | --- | --- | --- | --- | --- |
|  |  |  |  | **No. of events** | **Total follow-up time (patient-years)** | **No. of events** | **Total follow-up time (patient-years)** |
| **All** | 16,827 (100%) | 43.01 (SD: 16.33) | 28 (7-28) | 4,459 | 106,921.31 | 12,368 | 30,791.44 |
| **Female** | 9,007 (53.5%) | 43.96 (SD: 17.36) | 28 (7-28) | 2,391 | 16,873.15 | 6,616 | 57,372.69 |
| **Male** | 7,820 (46.5%) | 41.91 (SD: 14.97) | 28 (7-28) | 2,068 | 13,918.31 | 5,752 | 49,548.60 |
| **Gabapentin only** | 7,635 (45.4%) | 44.27 (SD: 16.50) | 28 (7-28) | 1,647 | 10,737.86 | 5,988 | 52,306.14 |
| **Pregabalin only** | 5,842 (34.7%) | 41.20 (SD: 16.41) | 28 (7-28) | 1,571 | 11,154.38 | 4,271 | 34,062.82 |
| **Black** | 408 (2.4%) | 44.04 (SD: 15.94) | 28 (7-28) | 91 | 641.00 | 317 | 2,759.72 |
| **South Asian** | 385 (2.3%) | 41.66 (SD: 15.45) | 28 (7-28) | 86 | 601.94 | 299 | 2,693.37 |
| **White** | 15,462 (91.9%) | 43.13 (SD: 16.38) | 28 (7-28) | 4,154 | 28,719.09 | 11,308 | 97,730.86 |
| **Other ethnic groups** | 527 (3.1%) | 39.87 (SD: 15.30) | 28 (7-28) | 113 | 772.75 | 414 | 3,440.60 |
| **Missing ethnicity** | 45 (0.3%) | 38.13 (SD: 16.80) | 28 (28-28) | 15 | 56.67 | 15 | 296.73 |

SD = Standard deviation; IQR = Interquartile range.
