# Supplementary material for: Association between gabapentinoid treatment, concurrent use with opioid or benzodiazepine and the risk of drug poisoning: A self-controlled case series study
Source: PLoS Med. 2026 Apr 16;23(4):e1005035. doi: 10.1371/journal.pmed.1005035 (PMC13086301; doi:10.1371/journal.pmed.1005035)
Supplement: S13 Table — (DOCX) [file pmed.1005035.s016.docx]

| **Risk window** | **Number of events** | **Patient-years** | **Crude incidence (per 100 patient-years) (95% CI)** | **aIRR (95% CI)** | ***P* value** |
| --- | --- | --- | --- | --- | --- |
| **Concomitant use with gabapentinoids, opioids and benzodiazepines (n=3,789)** |  |  |  |  |  |
| Under benzodiazepine treatment, opioid at reference and 90-day before gabapentinoid treatment | 31 | 70.15 | 44.19 (28.63, 59.74) | 5.91 (4.04, 8.64) | <0.001 |
| Under benzodiazepine treatment, opioid at reference and gabapentinoid treatment | 71 | 484.68 | 14.65 (11.24, 18.06) | 2.10 (1.58, 2.81) | <0.001 |
| Under opioid treatment, benzodiazepine at reference and 90-day before gabapentinoid treatment | 65 | 307.27 | 21.15 (16.01, 26.30) | 2.39 (1.83, 3.11) | <0.001 |
| Under opioid treatment, benzodiazepine at reference and gabapentinoid treatment | 306 | 2,328.38 | 13.14 (11.67, 14.61) | 1.59 (1.35, 1.89) | <0.001 |
| Under opioid treatment, benzodiazepine treatment and 90-day before gabapentinoid treatment | 37 | 113.62 | 32.57 (22.07, 43.06) | 3.96 (2.77, 5.68) | <0.001 |
| Under opioid treatment, benzodiazepine treatment and gabapentinoid treatment | 225 | 1,010.40 | 22.27 (19.36, 25.18) | 3.46 (2.80, 4.28) | <0.001 |
| Opioid at reference, benzodiazepine at reference and 90-day before gabapentinoid treatment | 85 | 434.72 | 19.55 (15.4, 23.71) | 2.25 (1.79, 2.82) | <0.001 |
| Opioid at reference, benzodiazepine at reference and under gabapentinoid treatment | 232 | 2,252.96 | 10.3 (8.97, 11.62) | 1.21 (1.02, 1.42) | 0.03 |
| Under opioid and benzodiazepine treatment, gabapentinoid at reference | 122 | 777.74 | 15.69 (12.90, 18.47) | 2.42 (1.92, 3.05) | <0.001 |
| Opioid, benzodiazepine and gabapentinoid at reference (reference) | 1,281 | 15,736.25 | 8.14 (7.69, 8.59) | 1.00 (1.00, 1.00) | NA |

n = Number of individuals included in the analysis; aIRR = Adjusted incidence rate ratio; CI = Confidence Interval; NA = Not Applicable

*All estimates are adjusted for age in 1-year age-band, seasonal effect, antiseizure medications, opioids, psychiatric medications and non-steroidal anti-inflammatory drug. *P* values were obtained from two-sided Wald tests.
