# Supplementary material for: Association between gabapentinoid treatment, concurrent use with opioid or benzodiazepine and the risk of drug poisoning: A self-controlled case series study
Source: PLoS Med. 2026 Apr 16;23(4):e1005035. doi: 10.1371/journal.pmed.1005035 (PMC13086301; doi:10.1371/journal.pmed.1005035)
Supplement: S14 Table — (DOCX) [file pmed.1005035.s017.docx]

| **Risk window** | **Number of events** | **Patient-years** | **Crude incidence (per 100 patient-years) (95% CI)** | **aIRR (95% CI)** | ***P* value** |
| --- | --- | --- | --- | --- | --- |
| **Aged 18-24 (n=1,281)** |  |  |  |  |  |
| 90 days before treatment | 136 | 274.70 | 49.51 (41.19, 57.83) | 2.00 (1.64, 2.44) | <0.001 |
| First 28 days of treatment period | 41 | 86.66 | 47.31 (32.83, 61.80) | 1.58 (1.13, 2.21) | 0.007 |
| 29-56 days of treatment period | 20 | 55.81 | 35.84 (20.13, 51.54) | 1.28 (0.81, 2.03) | 0.30 |
| 57-84 days of treatment period | 13 | 46.87 | 27.74 (12.66, 42.81) | 1.00 (0.57, 1.76) | 0.99 |
| Remaining time of treatment period | 104 | 450.23 | 23.10 (18.66, 27.54) | 1.00 (0.76, 1.31) | 0.99 |
| Reference period | 967 | 4,680.82 | 20.66 (19.36, 21.96) | 1.00 (1.00, 1.00) | NA |
| **Aged 25-34 (n=3,000)** |  |  |  |  |  |
| 90 days before treatment | 285 | 778.04 | 36.63 (32.38, 40.88) | 1.93 (1.69, 2.20) | <0.001 |
| First 28 days of treatment period | 77 | 253.37 | 30.39 (23.60, 37.18) | 1.34 (1.06, 1.71) | 0.02 |
| 29-56 days of treatment period | 56 | 170.70 | 32.81 (24.21, 41.40) | 1.51 (1.15, 2.00) | 0.003 |
| 57-84 days of treatment period | 32 | 148.53 | 21.54 (14.08, 29.01) | 1.02 (0.71, 1.46) | 0.90 |
| Remaining time of treatment period | 450 | 2,254.00 | 19.96 (18.12, 21.81) | 1.22 (1.05, 1.41) | 0.01 |
| Reference period | 2,100 | 12,961.13 | 16.20 (15.51, 16.90) | 1.00 (1.00, 1.00) | NA |
| **Aged 35-44 (n=3,890)** |  |  |  |  |  |
| 90 days before treatment | 357 | 1,010.49 | 35.33 (31.66, 38.99) | 2.06 (1.83, 2.32) | <0.001 |
| First 28 days of treatment period | 106 | 333.28 | 31.80 (25.75, 37.86) | 1.61 (1.31, 1.97) | <0.001 |
| 29-56 days of treatment period | 51 | 236.14 | 21.60 (15.67, 27.52) | 1.15 (0.86, 1.53) | 0.34 |
| 57-84 days of treatment period | 47 | 210.67 | 22.31 (15.93, 28.69) | 1.20 (0.89, 1.62) | 0.22 |
| Remaining time of treatment period | 797 | 4,252.88 | 18.74 (17.44, 20.04) | 1.21 (1.07, 1.37) | 0.003 |
| Reference period | 2,532 | 16,215.92 | 15.61 (15.01, 16.22) | 1.00 (1.00, 1.00) | NA |
| **Aged 45-54 (n=3,891)** |  |  |  |  |  |
| 90 days before treatment | 337 | 1,077.45 | 31.28 (27.94, 34.62) | 1.89 (1.68, 2.14) | <0.001 |
| First 28 days of treatment period | 102 | 354.20 | 28.80 (23.21, 34.39) | 1.53 (1.25, 1.89) | <0.001 |
| 29-56 days of treatment period | 57 | 243.24 | 23.43 (17.35, 29.52) | 1.30 (0.99, 1.70) | 0.06 |
| 57-84 days of treatment period | 46 | 215.98 | 21.30 (15.14, 27.45) | 1.18 (0.88, 1.59) | 0.28 |
| Remaining time of treatment period | 913 | 5,356.79 | 17.04 (15.94, 18.15) | 1.08 (0.95, 1.22) | 0.24 |
| Reference period | 2,436 | 16,274.20 | 14.97 (14.37, 15.56) | 1.00 (1.00, 1.00) | NA |
| **Aged 55-64 (n=2,135)** |  |  |  |  |  |
| 90 days before treatment | 223 | 554.46 | 40.22 (34.94, 45.50) | 2.44 (2.09, 2.85) | <0.001 |
| First 28 days of treatment period | 55 | 183.46 | 29.98 (22.06, 37.90) | 1.61 (1.22, 2.14) | 0.001 |
| 29-56 days of treatment period | 36 | 128.90 | 27.93 (18.80, 37.05) | 1.57 (1.11, 2.21) | 0.01 |
| 57-84 days of treatment period | 29 | 115.06 | 25.20 (16.03, 34.38) | 1.43 (0.98, 2.10) | 0.06 |
| Remaining time of treatment period | 551 | 3,079.64 | 17.89 (16.40, 19.39) | 1.16 (0.98, 1.36) | 0.08 |
| Reference period | 1,241 | 8,244.01 | 15.05 (14.22, 15.89) | 1.00 (1.00, 1.00) | NA |
| **Aged 65 or above (n=2,630)** |  |  |  |  |  |
| 90 days before treatment | 250 | 832.70 | 30.02 (26.30, 33.74) | 2.13 (1.85, 2.45) | <0.001 |
| First 28 days of treatment period | 139 | 272.25 | 51.06 (42.57, 59.54) | 3.13 (2.61, 3.76) | <0.001 |
| 29-56 days of treatment period | 53 | 183.09 | 28.95 (21.15, 36.74) | 1.86 (1.40, 2.46) | <0.001 |
| 57-84 days of treatment period | 40 | 158.50 | 25.24 (17.42, 33.06) | 1.64 (1.19, 2.27) | 0.003 |
| Remaining time of treatment period | 644 | 3,957.97 | 16.27 (15.01, 17.53) | 1.10 (0.96, 1.27) | 0.18 |
| Reference period | 1,504 | 13,690.87 | 10.99 (10.43, 11.54) | 1.00 (1.00, 1.00) | NA |

n = Number of individuals included in the analysis; aIRR = Adjusted incidence rate ratio; CI = Confidence Interval; NA = Not Applicable

*All estimates are adjusted for age in 1-year age-band, seasonal effect, antiseizure medications, opioids, psychiatric medications and non-steroidal anti-inflammatory drugs. *P* values were obtained from two-sided Wald tests.
