# Supplementary material for: Association between gabapentinoid treatment, concurrent use with opioid or benzodiazepine and the risk of drug poisoning: A self-controlled case series study
Source: PLoS Med. 2026 Apr 16;23(4):e1005035. doi: 10.1371/journal.pmed.1005035 (PMC13086301; doi:10.1371/journal.pmed.1005035)
Supplement: S15 Table — (DOCX) [file pmed.1005035.s018.docx]

| **Risk window** | **Number of events** | **Patient-years** | **Crude incidence (per 100 patient-years) (95% CI)** | **aIRR (95% CI)** | ***P* value** |
| --- | --- | --- | --- | --- | --- |
| **Black (n=408)** |  |  |  |  |  |
| 90 days before treatment | 37 | 158.08 | 23.41 (15.86, 30.95) | 1.79 (1.25, 2.58) | 0.002 |
| First 28 days of treatment period | 19 | 50.54 | 37.59 (20.69, 54.50) | 2.40 (1.46, 3.94) | 0.001 |
| 29-56 days of treatment period | 7 | 32.50 | 21.54 (5.58, 37.49) | 1.41 (0.65, 3.06) | 0.39 |
| 57-84 days of treatment period | 4 | 28.40 | 14.08 (0.28, 27.88) | 0.91 (0.33, 2.51) | 0.86 |
| Remaining time of treatment period | 61 | 529.56 | 11.52 (8.63, 14.41) | 0.77 (0.52, 1.16) | 0.21 |
| Reference period | 280 | 2,601.64 | 10.76 (9.50, 12.02) | 1.00 (1.00, 1.00) | NA |
| **South Asian (n=385)** |  |  |  |  |  |
| 90 days before treatment | 36 | 151.74 | 23.73 (15.97, 31.48) | 1.94 (1.35, 2.80) | <0.001 |
| First 28 days of treatment period | 12 | 49.00 | 24.49 (10.63, 38.34) | 1.74 (0.96, 3.16) | 0.07 |
| 29-56 days of treatment period | 8 | 29.57 | 27.06 (8.31, 45.80) | 2.00 (0.97, 4.13) | 0.06 |
| 57-84 days of treatment period | 4 | 24.56 | 16.29 (0.33, 32.25) | 1.21 (0.44, 3.31) | 0.71 |
| Remaining time of treatment period | 62 | 498.81 | 12.43 (9.34, 15.52) | 1.02 (0.67, 1.53) | 0.93 |
| Reference period | 263 | 2,541.63 | 10.35 (9.10, 11.60) | 1.00 (1.00, 1.00) | NA |
| **White (n=15,462)** |  |  |  |  |  |
| 90 days before treatment | 1,447 | 5,352.55 | 27.03 (25.64, 28.43) | 2.09 (1.97, 2.21) | <0.001 |
| First 28 days of treatment period | 476 | 1,750.77 | 27.19 (24.75, 29.63) | 1.81 (1.65, 2.00) | <0.001 |
| 29-56 days of treatment period | 244 | 1,203.63 | 20.27 (17.73, 22.82) | 1.41 (1.24, 1.61) | <0.001 |
| 57-84 days of treatment period | 198 | 1,061.19 | 18.66 (16.06, 21.26) | 1.32 (1.14, 1.52) | <0.001 |
| Remaining time of treatment period | 3,236 | 24,703.50 | 13.10 (12.65, 13.55) | 1.12 (1.05, 1.18) | <0.001 |
| Reference period | 9,861 | 92,378.31 | 10.67 (10.46, 10.89) | 1.00 (1.00, 1.00) | NA |

n = Number of individuals included in the analysis; aIRR = Adjusted incidence rate ratio; CI = Confidence Interval; NA = Not Applicable

*All estimates are adjusted for age in 1-year age-band, seasonal effect, antiseizure medications, opioids, psychiatric medications and non-steroidal anti-inflammatory drugs. P values were obtained from two-sided Wald tests.
