# Supplementary material for: Association between gabapentinoid treatment, concurrent use with opioid or benzodiazepine and the risk of drug poisoning: A self-controlled case series study
Source: PLoS Med. 2026 Apr 16;23(4):e1005035. doi: 10.1371/journal.pmed.1005035 (PMC13086301; doi:10.1371/journal.pmed.1005035)
Supplement: S16 Table — (DOCX) [file pmed.1005035.s019.docx]

| **Risk window** | **Number of events** | **Patient-years** | **Crude incidence (per 100 patient-years) (95% CI)** | **aIRR (95% CI)** | ***P* value** |
| --- | --- | --- | --- | --- | --- |
| **Under 0.5 DDD throughout observation period (n=5,572)** |  |  |  |  |  |
| 90 days before treatment | 472 | 1,652.55 | 28.56 (25.99, 31.14) | 1.98 (1.79, 2.19) | <0.001 |
| First 28 days of treatment period | 164 | 530.12 | 30.94 (26.20, 35.67) | 1.90 (1.61, 2.23) | <0.001 |
| 29-56 days of treatment period | 59 | 305.00 | 19.34 (14.41, 24.28) | 1.24 (0.96, 1.62) | 0.10 |
| 57-84 days of treatment period | 54 | 248.60 | 21.72 (15.93, 27.52) | 1.40 (1.07, 1.85) | 0.02 |
| Remaining time of treatment period | 618 | 4,490.29 | 13.76 (12.68, 14.85) | 1.03 (0.91, 1.16) | 0.63 |
| Reference period | 4,205 | 38,280.16 | 10.98 (10.65, 11.32) | 1.00 (1.00, 1.00) | NA |
| **Between 0.5 to 1.0 DDD throughout observation period (n=6,367)** |  |  |  |  |  |
| 90 days before treatment | 655 | 1,914.89 | 34.21 (31.59, 36.83) | 2.43 (2.23, 2.65) | <0.001 |
| First 28 days of treatment period | 182 | 630.32 | 28.87 (24.68, 33.07) | 1.72 (1.47, 2.00) | <0.001 |
| 29-56 days of treatment period | 108 | 449.02 | 24.05 (19.52, 28.59) | 1.50 (1.23, 1.83) | <0.001 |
| 57-84 days of treatment period | 70 | 402.64 | 17.39 (13.31, 21.46) | 1.11 (0.88, 1.42) | 0.38 |
| Remaining time of treatment period | 1,229 | 9,456.34 | 13.00 (12.27, 13.72) | 1.02 (0.93, 1.12) | 0.64 |
| Reference period | 4,123 | 37,834.57 | 10.90 (10.56, 11.23) | 1.00 (1.00, 1.00) | NA |
| **Over 1.0 DDD throughout observation period (n=2,298)** |  |  |  |  |  |
| 90 days before treatment | 173 | 597.36 | 28.96 (24.65, 33.28) | 2.13 (1.79, 2.53) | 0 |
| First 28 days of treatment period | 58 | 209.62 | 27.67 (20.55, 34.79) | 1.63 (1.23, 2.15) | 0.001 |
| 29-56 days of treatment period | 48 | 191.01 | 25.13 (18.02, 32.24) | 1.57 (1.16, 2.12) | 0.003 |
| 57-84 days of treatment period | 38 | 184.94 | 20.55 (14.01, 27.08) | 1.29 (0.92, 1.80) | 0.14 |
| Remaining time of treatment period | 960 | 7,330.44 | 13.10 (12.27, 13.92) | 1.14 (0.99, 1.31) | 0.06 |
| Reference period | 1,021 | 9,414.72 | 10.84 (10.18, 11.51) | 1.00 (1.00, 1.00) | NA |

DDD = Defined daily dose; n = Number of individuals included in the analysis; aIRR = Adjusted incidence rate ratio; CI = Confidence Interval; NA = Not Applicable

*All estimates are adjusted for age in 1-year age-band, seasonal effect, antiseizure medications, opioids, psychiatric medications and non-steroidal anti-inflammatory drugs. *P* values were obtained from two-sided Wald tests.
