# Supplementary material for: Association between gabapentinoid treatment, concurrent use with opioid or benzodiazepine and the risk of drug poisoning: A self-controlled case series study
Source: PLoS Med. 2026 Apr 16;23(4):e1005035. doi: 10.1371/journal.pmed.1005035 (PMC13086301; doi:10.1371/journal.pmed.1005035)
Supplement: S17 Table — (DOCX) [file pmed.1005035.s020.docx]

| **Risk window** | **Number of events** | **Patient-years** | **Crude incidence (per 100 patient-years) (95% CI)** | **aIRR (95% CI)** | ***P* value** |
| --- | --- | --- | --- | --- | --- |
| **Individuals who had mental health conditions before the end of observation period (n=15,976)** |  |  |  |  |  |
| 90 days before treatment | 1,499 | 5,594.61 | 26.79 (25.44, 28.15) | 2.06 (1.95, 2.19) | <0.001 |
| First 28 days of treatment period | 459 | 1,827.85 | 25.11 (22.81, 27.41) | 1.67 (1.52, 1.84) | <0.001 |
| 29-56 days of treatment period | 255 | 1,252.75 | 20.36 (17.86, 22.85) | 1.42 (1.25, 1.61) | <0.001 |
| 57-84 days of treatment period | 199 | 1,102.93 | 18.04 (15.54, 20.55) | 1.27 (1.10, 1.46) | 0.001 |
| Remaining time of treatment period | 3,276 | 25,276.15 | 12.96 (12.52, 13.40) | 1.09 (1.03, 1.15) | 0.004 |
| Reference period | 10,288 | 95,844.27 | 10.73 (10.53, 10.94) | 1.00 (1.00, 1.00) | NA |
| **Individuals who had substance misuse before the end of observation period (n=11,809)** |  |  |  |  |  |
| 90 days before treatment | 1,075 | 4,124.31 | 26.06 (24.51, 27.62) | 2.00 (1.87, 2.14) | <0.001 |
| First 28 days of treatment period | 308 | 1,347.93 | 22.85 (20.30, 25.40) | 1.52 (1.35, 1.71) | <0.001 |
| 29-56 days of treatment period | 187 | 933.66 | 20.03 (17.16, 22.90) | 1.39 (1.20, 1.61) | <0.001 |
| 57-84 days of treatment period | 144 | 823.86 | 17.48 (14.62, 20.33) | 1.23 (1.04, 1.45) | 0.02 |
| Remaining time of treatment period | 2,464 | 18,850.09 | 13.07 (12.56, 13.59) | 1.11 (1.04, 1.19) | 0.002 |
| Reference period | 7,631 | 70,654.31 | 10.80 (10.56, 11.04) | 1.00 (1.00, 1.00) | NA |
| **Individuals who had bipolar and mania before the end of observation period (n=** **1,207)** |  |  |  |  |  |
| 90 days before treatment | 95 | 398.95 | 23.81 (19.02, 28.60) | 1.73 (1.39, 2.17) | <0.001 |
| First 28 days of treatment period | 31 | 131.21 | 23.63 (15.31, 31.94) | 1.58 (1.09, 2.29) | 0.01 |
| 29-56 days of treatment period | 15 | 96.63 | 15.52 (7.67, 23.38) | 1.06 (0.63, 1.79) | 0.82 |
| 57-84 days of treatment period | 17 | 86.82 | 19.58 (10.27, 28.89) | 1.36 (0.83, 2.22) | 0.22 |
| Remaining time of treatment period | 230 | 2,106.17 | 10.92 (9.51, 12.33) | 0.93 (0.76, 1.15) | 0.52 |
| Reference period | 819 | 6,980.05 | 11.73 (10.93, 12.54) | 1.00 (1.00, 1.00) | NA |
| **Individuals who had depression before the end of observation period (n=12,918)** |  |  |  |  |  |
| 90 days before treatment | 1,180 | 4,559.50 | 25.88 (24.40, 27.36) | 2.01 (1.88, 2.14) | <0.001 |
| First 28 days of treatment period | 377 | 1,487.68 | 25.34 (22.78, 27.90) | 1.70 (1.53, 1.89) | <0.001 |
| 29-56 days of treatment period | 201 | 1,034.62 | 19.43 (16.74, 22.11) | 1.37 (1.19, 1.58) | <0.001 |
| 57-84 days of treatment period | 167 | 915.68 | 18.24 (15.47, 21.00) | 1.29 (1.11, 1.51) | 0.001 |
| Remaining time of treatment period | 2,686 | 21,290.24 | 12.62 (12.14, 13.09) | 1.08 (1.01, 1.15) | 0.02 |
| Reference period | 8,307 | 76,986.84 | 10.79 (10.56, 11.02) | 1.00 (1.00, 1.00) | NA |
| **Individuals who had anxiety before the end of observation period (n=10,870)** |  |  |  |  |  |
| 90 days before treatment | 988 | 3,829.71 | 25.80 (24.19, 27.41) | 1.99 (1.86, 2.14) | <0.001 |
| First 28 days of treatment period | 287 | 1,246.29 | 23.03 (20.36, 25.69) | 1.56 (1.38, 1.77) | <0.001 |
| 29-56 days of treatment period | 160 | 870.08 | 18.39 (15.54, 21.24) | 1.29 (1.10, 1.52) | 0.002 |
| 57-84 days of treatment period | 146 | 772.53 | 18.90 (15.83, 21.96) | 1.34 (1.13, 1.58) | 0.001 |
| Remaining time of treatment period | 2,269 | 18,035.28 | 12.58 (12.06, 13.10) | 1.06 (0.99, 1.13) | 0.11 |
| Reference period | 7,020 | 65,141.96 | 10.78 (10.52, 11.03) | 1.00 (1.00, 1.00) | NA |
| **Individuals who had schizophrenia before end of observation period (n=602)** |  |  |  |  |  |
| 90 days before treatment | 41 | 189.40 | 21.65 (15.02, 28.27) | 1.47 (1.05, 2.06) | 0.03 |
| First 28 days of treatment period | 16 | 62.52 | 25.59 (13.05, 38.13) | 1.53 (0.91, 2.57) | 0.11 |
| 29-56 days of treatment period | 10 | 46.46 | 21.52 (8.18, 34.87) | 1.30 (0.68, 2.48) | 0.42 |
| 57-84 days of treatment period | 9 | 42.10 | 21.38 (7.41, 35.35) | 1.36 (0.69, 2.67) | 0.37 |
| Remaining time of treatment period | 124 | 1,088.56 | 11.39 (9.39, 13.40) | 0.87 (0.65, 1.16) | 0.35 |
| Reference period | 402 | 3,399.58 | 11.82 (10.67, 12.98) | 1.00 (1.00, 1.00) | NA |
| **Individuals who had other psychosis before end of observation period (n=1,171)** |  |  |  |  |  |
| 90 days before treatment | 80 | 391.96 | 20.41 (15.94, 24.88) | 1.40 (1.10, 1.78) | 0.007 |
| First 28 days of treatment period | 27 | 128.12 | 21.07 (13.12, 29.02) | 1.30 (0.88, 1.93) | 0.19 |
| 29-56 days of treatment period | 15 | 95.20 | 15.76 (7.78, 23.73) | 1.01 (0.60, 1.69) | 0.98 |
| 57-84 days of treatment period | 14 | 86.87 | 16.12 (7.67, 24.56) | 1.03 (0.60, 1.76) | 0.92 |
| Remaining time of treatment period | 249 | 2,096.82 | 11.88 (10.40, 13.35) | 0.91 (0.75, 1.12) | 0.39 |
| Reference period | 786 | 6,705.51 | 11.72 (10.90, 12.54) | 1.00 (1.00, 1.00) | NA |
| **Individuals who had insomnia before end of observation period (n=4,473)** |  |  |  |  |  |
| 90 days before treatment | 382 | 1,649.66 | 23.16 (20.83, 25.48) | 1.91 (1.71, 2.14) | <0.001 |
| First 28 days of treatment period | 119 | 535.43 | 22.22 (18.23, 26.22) | 1.69 (1.40, 2.04) | <0.001 |
| 29-56 days of treatment period | 70 | 370.83 | 18.88 (14.45, 23.30) | 1.49 (1.17, 1.90) | 0.001 |
| 57-84 days of treatment period | 60 | 327.26 | 18.33 (13.69, 22.97) | 1.47 (1.14, 1.91) | 0.004 |
| Remaining time of treatment period | 974 | 7,983.89 | 12.20 (11.43, 12.97) | 1.12 (1.01, 1.25) | 0.03 |
| Reference period | 2,868 | 27,442.92 | 10.45 (10.07, 10.83) | 1.00 (1.00, 1.00) | NA |
| **Individuals who were not diagnosed with mental health conditions before end of observation period (n=851)** |  |  |  |  |  |
| 90 days before treatment | 89 | 274.20 | 32.46 (25.71, 39.20) | 2.71 (2.14, 3.45) | <0.001 |
| First 28 days of treatment period | 61 | 89.53 | 68.13 (51.04, 85.23) | 4.91 (3.69, 6.52) | <0.001 |
| 29-56 days of treatment period | 18 | 56.96 | 31.60 (17.00, 46.20) | 2.40 (1.47, 3.92) | <0.001 |
| 57-84 days of treatment period | 8 | 48.51 | 16.49 (5.06, 27.92) | 1.30 (0.64, 2.66) | 0.47 |
| Remaining time of treatment period | 183 | 1,136.76 | 16.10 (13.77, 18.43) | 1.63 (1.26, 2.13) | <0.001 |
| Reference period | 492 | 5,208.21 | 9.45 (8.61, 10.28) | 1.00 (1.00, 1.00) | NA |

n = Number of individuals included in the analysis; aIRR = Adjusted incidence rate ratio; CI = Confidence Interval; NA = Not Applicable

*All estimates are adjusted for age in 1-year age-band, seasonal effect, antiseizure medications, opioids, psychiatric medications and non-steroidal anti-inflammatory drugs. *P* values were obtained from two-sided Wald tests.
