# Supplementary material for: Association between gabapentinoid treatment, concurrent use with opioid or benzodiazepine and the risk of drug poisoning: A self-controlled case series study
Source: PLoS Med. 2026 Apr 16;23(4):e1005035. doi: 10.1371/journal.pmed.1005035 (PMC13086301; doi:10.1371/journal.pmed.1005035)
Supplement: S18 Table — (DOCX) [file pmed.1005035.s021.docx]

| **Risk window** | **Number of events** | **Patient-years** | **Crude incidence (per 100 patient-years) (95% CI)** | **aIRR (95% CI)** | ***P* value** |
| --- | --- | --- | --- | --- | --- |
| **Accidental poisoning only (n=4,258)** |  |  |  |  |  |
| 90 days before treatment | 417 | 1,546.67 | 26.96 (24.37, 29.55) | 2.44 (2.19, 2.73) | <0.001 |
| First 28 days of treatment period | 166 | 492.13 | 33.73 (28.60, 38.86) | 2.73 (2.32, 3.21) | <0.001 |
| 29-56 days of treatment period | 86 | 342.64 | 25.10 (19.79, 30.40) | 2.17 (1.74, 2.70) | <0.001 |
| 57-84 days of treatment period | 50 | 300.90 | 16.62 (12.01, 21.22) | 1.46 (1.10, 1.94) | 0.01 |
| Remaining time of treatment period | 1,153 | 7,440.43 | 15.50 (14.60, 16.39) | 1.58 (1.42, 1.76) | <0.001 |
| Reference period | 2,386 | 25,640.23 | 9.31 (8.93, 9.68) | 1.00 (1.00, 1.00) | NA |
| **Intentional self-poisoning only (n=7,740)** |  |  |  |  |  |
| 90 days before treatment | 617 | 2,784.12 | 22.16 (20.41, 23.91) | 1.76 (1.61, 1.92) | <0.001 |
| First 28 days of treatment period | 184 | 885.62 | 20.78 (17.77, 23.78) | 1.49 (1.28, 1.73) | <0.001 |
| 29-56 days of treatment period | 115 | 604.44 | 19.03 (15.55, 22.5) | 1.44 (1.19, 1.74) | <0.001 |
| 57-84 days of treatment period | 95 | 532.81 | 17.83 (14.24, 21.42) | 1.35 (1.10, 1.66) | 0.004 |
| Remaining time of treatment period | 1,319 | 12,038.29 | 10.96 (10.37, 11.55) | 1.00 (0.92, 1.09) | 0.97 |
| Reference period | 5,410 | 49,515.91 | 10.93 (10.63, 11.22) | 1.00 (1.00, 1.00) | NA |

ICD10 = International Statistical Classification of Diseases and Related Health Problems 10th Revision; n = Number of individuals included in the analysis; aIRR = Adjusted incidence rate ratio; CI = Confidence Interval; NA = Not Applicable

*All estimates are adjusted for age in 1-year age-band, seasonal effect, antiseizure medications, opioids, psychiatric medications and non-steroidal anti-inflammatory drugs. *P* values were obtained from two-sided Wald tests.
