# Supplementary material for: Association between gabapentinoid treatment, concurrent use with opioid or benzodiazepine and the risk of drug poisoning: A self-controlled case series study
Source: PLoS Med. 2026 Apr 16;23(4):e1005035. doi: 10.1371/journal.pmed.1005035 (PMC13086301; doi:10.1371/journal.pmed.1005035)
Supplement: S19 Table — (DOCX) [file pmed.1005035.s022.docx]

| **Risk window** | **Number of events** | **Patient-years** | **Crude incidence (per 100 patient-years) (95% CI)** | **aIRR (95% CI)** | ***P* value** |
| --- | --- | --- | --- | --- | --- |
| **Exclusion of patients who recorded dead within 6 months after event (n=16,379)** |  |  |  |  |  |
| 90 days before treatment | 1,554 | 5,728.02 | 27.13 (25.78, 28.48) | 2.12 (2.01, 2.25) | <0.001 |
| First 28 days of treatment period | 486 | 1,870.93 | 25.98 (23.67, 28.29) | 1.77 (1.61, 1.94) | <0.001 |
| 29-56 days of treatment period | 258 | 1,278.40 | 20.18 (17.72, 22.64) | 1.43 (1.26, 1.62) | <0.001 |
| 57-84 days of treatment period | 198 | 1,124.35 | 17.61 (15.16, 20.06) | 1.26 (1.09, 1.45) | 0.002 |
| Remaining time of treatment period | 3,312 | 25,927.10 | 12.77 (12.34, 13.21) | 1.09 (1.03, 1.16) | 0.002 |
| Reference period | 10,571 | 99,128.24 | 10.66 (10.46, 10.87) | 1.00 (1.00, 1.00) | NA |
| **Observation starts from neuropathic pain or chronic pain diagnosis (n=10,062)** |  |  |  |  |  |
| 90 days before treatment | 1,034 | 3,465.02 | 29.84 (28.02, 31.66) | 1.93 (1.80, 2.07) | <0.001 |
| First 28 days of treatment period | 373 | 1,166.31 | 31.98 (28.74, 35.23) | 1.76 (1.58, 1.97) | <0.001 |
| 29-56 days of treatment period | 179 | 791.97 | 22.60 (19.29, 25.91) | 1.31 (1.12, 1.53) | 0.001 |
| 57-84 days of treatment period | 126 | 694.12 | 18.15 (14.98, 21.32) | 1.08 (0.90, 1.29) | 0.43 |
| Remaining time of treatment period | 2,256 | 15,703.73 | 14.37 (13.77, 14.96) | 1.09 (1.02, 1.17) | 0.01 |
| Reference period | 6,094 | 47,359.20 | 12.87 (12.54, 13.19) | 1.00 (1.00, 1.00) | NA |
| **Limiting individuals with at least 2 prescriptions of gabapentinoids (n=13,682)** |  |  |  |  |  |
| 90 days before treatment | 1,345 | 5,112.33 | 26.31 (24.90, 27.72) | 2.12 (2.00, 2.26) | <0.001 |
| First 28 days of treatment period | 420 | 1,687.82 | 24.88 (22.50, 27.26) | 1.74 (1.57, 1.92) | <0.001 |
| 29-56 days of treatment period | 269 | 1,295.07 | 20.77 (18.29, 23.25) | 1.47 (1.30, 1.66) | <0.001 |
| 57-84 days of treatment period | 207 | 1,150.86 | 17.99 (15.54, 20.44) | 1.28 (1.11, 1.48) | 0.001 |
| Remaining time of treatment period | 3,459 | 26,412.91 | 13.10 (12.66, 13.53) | 1.12 (1.06, 1.18) | <0.001 |
| Reference period | 7,982 | 76,754.06 | 10.40 (10.17, 10.63) | 1.00 (1.00, 1.00) | NA |
| **Changing before treatment period to 120 days (n=16,827)** |  |  |  |  |  |
| 180 days before treatment | 1,928 | 7,704.18 | 25.03 (23.91, 26.14) | 1.98 (1.88, 2.09) | <0.001 |
| First 28 days of treatment period | 520 | 1,917.38 | 27.12 (24.79, 29.45) | 1.85 (1.68, 2.02) | <0.001 |
| 29-56 days of treatment period | 273 | 1,309.71 | 20.84 (18.37, 23.32) | 1.48 (1.31, 1.68) | <0.001 |
| 57-84 days of treatment period | 207 | 1,151.44 | 17.98 (15.53, 20.43) | 1.29 (1.12, 1.49) | <0.001 |
| Remaining time of treatment period | 3,459 | 26,412.91 | 13.10 (12.66, 13.53) | 1.13 (1.07, 1.19) | <0.001 |
| Reference period | 10,440 | 99,217.10 | 10.52 (10.32, 10.72) | 1.00 (1.00, 1.00) | NA |
| **Changing before treatment period to 60 days (n=16,827)** |  |  |  |  |  |
| 30 days before treatment | 1,199 | 3,939.39 | 30.44 (28.71, 32.16) | 2.27 (2.13, 2.41) | <0.001 |
| First 28 days of treatment period | 520 | 1,917.38 | 27.12 (24.79, 29.45) | 1.77 (1.62, 1.94) | <0.001 |
| 29-56 days of treatment period | 273 | 1,309.71 | 20.84 (18.37, 23.32) | 1.42 (1.26, 1.61) | <0.001 |
| 57-84 days of treatment period | 207 | 1,151.44 | 17.98 (15.53, 20.43) | 1.24 (1.08, 1.43) | 0.003 |
| Remaining time of treatment period | 3,459 | 26,412.91 | 13.10 (12.66, 13.53) | 1.08 (1.02, 1.14) | 0.01 |
| Reference period | 11,169 | 102,981.90 | 10.85 (10.64, 11.05) | 1.00 (1.00, 1.00) | NA |
| **Only adjust for age and season (n=16,827)** |  |  |  |  |  |
| 90 days before treatment | 1,588 | 5,868.81 | 27.06 (25.73, 28.39) | 2.17 (2.05, 2.29) | <0.001 |
| First 28 days of treatment period | 520 | 1,917.38 | 27.12 (24.79, 29.45) | 2.04 (1.86, 2.24) | <0.001 |
| 29-56 days of treatment period | 273 | 1,309.71 | 20.84 (18.37, 23.32) | 1.59 (1.41, 1.80) | <0.001 |
| 57-84 days of treatment period | 207 | 1,151.44 | 17.98 (15.53, 20.43) | 1.39 (1.21, 1.60) | <0.001 |
| Remaining time of treatment period | 3,459 | 26,412.91 | 13.10 (12.66, 13.53) | 1.22 (1.16, 1.29) | <0.001 |
| Reference period | 10,780 | 101,052.50 | 10.67 (10.47, 10.87) | 1.00 (1.00, 1.00) | NA |
| **Not combining GABA prescriptions that were less than or equal to 90 days apart (n=16,791)** |  |  |  |  |  |
| 90 days before treatment | 2,189 | 10,511.39 | 20.83 (19.95, 21.70) | 1.83 (1.73, 1.92) | <0.001 |
| First 28 days of treatment period | 1,659 | 10,550.84 | 15.72 (14.97, 16.48) | 1.32 (1.24, 1.40) | <0.001 |
| 29-56 days of treatment period | 700 | 4,728.04 | 14.81 (13.71, 15.90) | 1.26 (1.16, 1.37) | <0.001 |
| 57-84 days of treatment period | 370 | 2,671.98 | 13.85 (12.44, 15.26) | 1.18 (1.06, 1.32) | 0.003 |
| Remaining time of treatment period | 1,093 | 8,045.21 | 13.59 (12.78, 14.39) | 1.20 (1.11, 1.30) | <0.001 |
| Reference period | 10,780 | 100,931.10 | 10.68 (10.48, 10.88) | 1.00 (1.00, 1.00) | NA |

n = Number of individuals included in the analysis; aIRR = Adjusted incidence rate ratio; CI = Confidence Interval; NA = Not Applicable; GABA = Gabapentinoid

*All estimates are adjusted for age in 1-year age-band, seasonal effect, antiseizure medications, opioids, psychiatric medications and non-steroidal anti-inflammatory drug. *P* values were obtained from two-sided Wald tests.
