# Supplementary material for: Association between gabapentinoid treatment, concurrent use with opioid or benzodiazepine and the risk of drug poisoning: A self-controlled case series study
Source: PLoS Med. 2026 Apr 16;23(4):e1005035. doi: 10.1371/journal.pmed.1005035 (PMC13086301; doi:10.1371/journal.pmed.1005035)
Supplement: S20 Table — (DOCX) [file pmed.1005035.s023.docx]

| **Risk window** | **Number of events** | **Patient-years** | **Crude incidence (per 100 patient-years) (95% CI)** | **aIRR (95% CI)** | ***P* value** |
| --- | --- | --- | --- | --- | --- |
| **Used both gabapentinoid and opioid during observation period and remove patients who had recorded death 6 months after event (n=8,063)** |  |  |  |  |  |
| Opioid at reference and 90 days before gabapentinoid treatment | 281 | 1,211.83 | 23.19 (20.48, 25.90) | 2.04 (1.80, 2.32) | <0.001 |
| Opioid at reference and first 28 days of gabapentinoid treatment | 92 | 419.30 | 21.94 (17.46, 26.43) | 1.75 (1.41, 2.16) | <0.001 |
| Opioid at reference and 29-56 days of gabapentinoid treatment | 43 | 274.50 | 15.66 (10.98, 20.35) | 1.27 (0.93, 1.72) | 0.13 |
| Opioid at reference and 57-84 days of gabapentinoid treatment | 32 | 236.48 | 13.53 (8.84, 18.22) | 1.10 (0.77, 1.56) | 0.59 |
| Opioid at reference and remaining time of gabapentinoid treatment | 522 | 4,569.75 | 11.42 (10.44, 12.40) | 1.01 (0.89, 1.13) | 0.91 |
| Opioid treatment period and 90 days before gabapentinoid treatment | 262 | 911.88 | 28.73 (25.25, 32.21) | 2.39 (2.08, 2.74) | <0.001 |
| Opioid treatment period and first 28 days of gabapentinoid treatment | 114 | 403.36 | 28.26 (23.07, 33.45) | 2.01 (1.66, 2.45) | <0.001 |
| Opioid treatment period and 29-56 days of gabapentinoid treatment | 63 | 277.19 | 22.73 (17.12, 28.34) | 1.67 (1.29, 2.17) | <0.001 |
| Opioid treatment period and 57-84 days of gabapentinoid treatment | 50 | 245.91 | 20.33 (14.70, 25.97) | 1.52 (1.14, 2.03) | 0.004 |
| Opioid treatment period and remaining time of gabapentinoid treatment | 839 | 5,729.79 | 14.64 (13.65, 15.63) | 1.34 (1.20, 1.50) | <0.001 |
| Opioid treatment period and gabapentinoid at reference | 1,021 | 8,150.11 | 12.53 (11.76, 13.30) | 1.25 (1.14, 1.37) | <0.001 |
| Both gabapentinoid and opioid were at reference (reference) | 3,783 | 38,480.54 | 9.83 (9.52, 10.14) | 1.00 (1.00, 1.00) | NA |
| **Used both gabapentinoid and benzodiazepine during observation period and remove patients who had recorded death 6 months after event (n=7,113)** |  |  |  |  |  |
| Benzodiazepine at reference and 90 days before gabapentinoid treatment | 352 | 1,811.62 | 19.43 (17.40, 21.46) | 2.00 (1.78, 2.24) | <0.001 |
| Benzodiazepine at reference and first 28 days of gabapentinoid treatment | 112 | 605.55 | 18.50 (15.07, 21.92) | 1.77 (1.46, 2.14) | <0.001 |
| Benzodiazepine at reference and 29-56 days of gabapentinoid treatment | 67 | 423.50 | 15.82 (12.03, 19.61) | 1.54 (1.20, 1.97) | 0.001 |
| Benzodiazepine at reference and 57-84 days of gabapentinoid treatment | 53 | 371.29 | 14.27 (10.43, 18.12) | 1.38 (1.05, 1.82) | 0.02 |
| Benzodiazepine at reference and remaining time of gabapentinoid treatment | 923 | 8,790.40 | 10.50 (9.82, 11.18) | 1.12 (1.02, 1.24) | 0.02 |
| Benzodiazepine treatment period and 90 days before gabapentinoid treatment | 146 | 393.14 | 37.14 (31.11, 43.16) | 4.33 (3.61, 5.19) | <0.001 |
| Benzodiazepine treatment period and first 28 days of gabapentinoid treatment | 66 | 166.79 | 39.57 (30.02, 49.12) | 3.92 (3.03, 5.07) | <0.001 |
| Benzodiazepine treatment period and 29-56 days of gabapentinoid treatment | 34 | 116.59 | 29.16 (19.36, 38.96) | 3.04 (2.15, 4.31) | <0.001 |
| Benzodiazepine treatment period and 57-84 days of gabapentinoid treatment | 19 | 104.12 | 18.25 (10.04, 26.45) | 1.90 (1.20, 3.00) | 0.006 |
| Benzodiazepine treatment period and remaining time of gabapentinoid treatment | 422 | 2,653.07 | 15.91 (14.39, 17.42) | 2.20 (1.91, 2.54) | <0.001 |
| Benzodiazepine treatment period and gabapentinoid at reference | 739 | 3,859.87 | 19.15 (17.77, 20.53) | 2.81 (2.54, 3.10) | <0.001 |
| Both gabapentinoid and benzodiazepine were at reference (reference) | 3,385 | 37,922.30 | 8.93 (8.63, 9.23) | 1.00 (1.00, 1.00) | NA |

n = Number of individuals included in the analysis; aIRR = Adjusted incidence rate ratio; CI = Confidence Interval

*All estimates are adjusted for age in 1-year age-band, seasonal effect, antiseizure medications, opioids, psychiatric medications and non-steroidal anti-inflammatory drugs. *P* values were obtained from two-sided Wald tests.
