# Supplementary material for: Association between gabapentinoid treatment, concurrent use with opioid or benzodiazepine and the risk of drug poisoning: A self-controlled case series study
Source: PLoS Med. 2026 Apr 16;23(4):e1005035. doi: 10.1371/journal.pmed.1005035 (PMC13086301; doi:10.1371/journal.pmed.1005035)
Supplement: S21 Table — (DOCX) [file pmed.1005035.s024.docx]

| **Risk Period** | **aIRR (95% CI)** | ***P* value** |
| --- | --- | --- |
| **Event-dependent observation** |  |  |
| 90 days before treatment | 2.11 (2.00, 2.23) | <0.001 |
| Gabapentinoid treatment period | 1.28 (1.22, 1.34) | <0.001 |
| **Event-dependent exposure** |  |  |
| First 28 days of GABA treatment | 1.78 (1.60, 1.99) | <0.001 |
| 29-56 days of GABA treatment | 1.44 (1.26, 1.65) | <0.001 |
| Remaining time of GABA treatment period | 1.40 (1.28, 1.53) | <0.001 |

n = Number of individuals included in the analysis; aIRR = Adjusted incidence rate ratio; CI = Confidence interval

* *P* values were obtained from two-sided Wald tests.
