# Supplementary material for: Association between gabapentinoid treatment, concurrent use with opioid or benzodiazepine and the risk of drug poisoning: A self-controlled case series study
Source: PLoS Med. 2026 Apr 16;23(4):e1005035. doi: 10.1371/journal.pmed.1005035 (PMC13086301; doi:10.1371/journal.pmed.1005035)
Supplement: S22 Table — (DOCX) [file pmed.1005035.s025.docx]

| **Risk window** | **Number of events** | **Patient-years** | **Crude incidence (per 100 patient-years) (95% CI)** | **aIRR (95% CI)** | ***P* value** |
| --- | --- | --- | --- | --- | --- |
| **Negative control: Food Poisoning (n=1,328)** |  |  |  |  |  |
| 90 days before treatment | 71 | 489.03 | 14.52 (11.14, 17.90) | 1.25 (0.98, 1.61) | 0.08 |
| First 28 days of treatment period | 19 | 155.27 | 12.24 (6.73, 17.74) | 1.01 (0.64, 1.60) | 0.96 |
| 29-56 days of treatment period | 11 | 87.95 | 12.51 (5.12, 19.90) | 1.07 (0.59, 1.96) | 0.82 |
| 57-84 days of treatment period | 6 | 71.38 | 8.41 (1.68, 15.13) | 0.73 (0.33, 1.64) | 0.45 |
| Remaining time of treatment period | 151 | 1,312.01 | 11.51 (9.67, 13.34) | 1.10 (0.87, 1.40) | 0.42 |
| Reference period | 1,070 | 10,273.50 | 10.42 (9.79, 11.04) | 1.00 (1.00, 1.00) | NA |

n = Number of individuals included in the analysis; aIRR = Adjusted incidence rate ratio; CI = Confidence interval

*All estimates are adjusted for age in 1-year age-band, seasonal effect, antiseizure medications, opioids, psychiatric medications and non-steroidal anti-inflammatory drugs. *P* values were obtained from two-sided Wald tests.
