# Supplementary material for: Association between gabapentinoid treatment, concurrent use with opioid or benzodiazepine and the risk of drug poisoning: A self-controlled case series study
Source: PLoS Med. 2026 Apr 16;23(4):e1005035. doi: 10.1371/journal.pmed.1005035 (PMC13086301; doi:10.1371/journal.pmed.1005035)
Supplement: S23 Table — (DOCX) [file pmed.1005035.s026.docx]

| **Analyses** | **Total number of patients** | **Odd ratios (95% CI)** | ***P* value** |
| --- | --- | --- | --- |
| Case-crossover alone | 13,333 | 1.79 (1.58, 2.02) | <0.001 |
| Control crossover | 11,324 | 1.33 (1.12, 1.53) | <0.001 |
| Case-case-time-control | 11,324 | 1.36 (1.12, 1.65) | 0.002 |

CI = Confidence interval

*All estimates are adjusted for age, antiseizure medications, opioids, psychiatric medications and non-steroidal anti-inflammatory drugs. *P* values were obtained from two-sided Wald tests.
