# Supplementary material for: Association between gabapentinoid treatment, concurrent use with opioid or benzodiazepine and the risk of drug poisoning: A self-controlled case series study
Source: PLoS Med. 2026 Apr 16;23(4):e1005035. doi: 10.1371/journal.pmed.1005035 (PMC13086301; doi:10.1371/journal.pmed.1005035)
Supplement: S1 Protocol — (DOCX) [file pmed.1005035.s039.docx]

**The risk of adverse psychiatric and somatic outcomes with gabapentinoid use: protocol of a UK population-based study using electronic health records**

Andrew S.C. Yuen, School of Pharmacy, University College London

**Background and rationale**

**a. Gabapentinoids**

Gabapentinoids are a class of medications which was first introduced in the United States (US) and the United Kingdom (UK) in 1993.^1^ The two major agents, gabapentin and pregabalin, were originally developed as antiseizure agents.^2^ They were developed as analogues of gamma-amino-butyric acid (GABA).^3^ However, they do not exhibit any effect on the GABA receptors, nor convert into GABA nor GABA receptor agonists.^4,5^ They also do not appear to control the transport of GABA or its metabolism.^6^

*Mechanism of action and distribution of gabapentinoids*

The mechanism of action of gabapentinoids are not fully understood. Their extensive range of clinical actions is believed to be primarily the result of their inhibitory properties on neuronal voltage-gated calcium channel currents, via impairing the trafficking function of the α2δ subunits, reducing the signal leading to the release of neurotransmitters between synapses in the brain tissue.^7,8^ The α2δ subunits of voltage gated calcium channels are linked to several biological functions, such as enhancing the presence of functional calcium channels on the cell surface, increasing the calcium current within cells, modifying the voltage-dependent activation and inactivation of calcium channels, and accelerating the rates at which these channels activate and deactivate.^4^

Voltage-gated calcium channels are involved in multiple physiological roles in our body. For instance, in the central nervous system, voltage-gated calcium channels also play a role in synaptic transmission.^9^ Activation of voltage-gated calcium channels in cardiac or vascular smooth muscle cells can lead to smooth muscle contraction, which regulates our cardiac rhythm and blood pressure, respectively.^10,11^ In endocrine cells, entry of calcium ions through the voltage-gated calcium channels initiates the secretion of hormones ,such as insulin.^12^ With such wide range physiological functions that voltage-gated calcium channels are involved, despite a lower level found in parts of our body other than the brain, the consumption of gabapentinoids may still have an effect in multiple body systems other than the central nervous system.

With the different pharmacokinetic profiles between gabapentin and pregabalin, the clinical effects exhibited by the two agents may differ from one another and it will be worth investigating how their differences in pharmacokinetic profiles affect their interactions with other conditions or medications.

**b. On and off-label uses of Gabapentinoids**

Licensed indications of gabapentinoids from across the world have since expanded to conditions including neuropathic pain, fibromyalgia, postherpetic neuralgia, restless legs syndrome, generalised anxiety disorder, menopausal symptoms and complications of multiple sclerosis.^13-16^ In addition to their licensed indications, off-label prescribing of gabapentinoids also accounted for a considerable proportion of their use.^17,18^ Given their effects on intracellular calcium levels, a considerable proportion of gabapentinoid prescriptions are for the treatment of mental health symptoms and diagnoses, such as insomnia and bipolar disorder, which, however, have limited evidence of efficacy to support their use.^2,19^

**c. Psychiatric effects of gabapentinoids**

A previous population based cohort study conducted in Sweden suggested that the use of gabapentinoid was associated with an increase hazards of suicidal behaviour and deaths from suicide, unintentional overdoses head/ body injuries and road traffic incidents and offences.^20^ However, such increase is only conclusive for patients at the age of 15-24 years but not for the other age groups.^20^ A recent analysis of studies examining the use of different anticonvulsants, such as pregabalin, has identified a higher likelihood of suicidal thoughts or behaviours when compared to a placebo.^21^ However, The existing studies on this subject have demonstrated a lack of agreement and are hindered by various methodological constraints.^21^

The available evidence on the correlation between gabapentinoids and various psychosocial adverse outcomes is limited. There have been only a few individual case reports describing an increased aggression in children with psychiatric disorders who were taking gabapentin,^22-24^ which are not sufficient to support causality. The lack of evidence in supporting the use of gabapentinoids with other psychiatric medications or in patients with psychiatric co-morbidities should be promptly addressed to ensure patients’ quality of life will not be jeopardised.

**Aims**

To investigate the association between the use of gabapentinoids and risks of psychiatric and somatic outcomes.

**Hypothesis**

The exposure to gabapentinoids will increase the risk of psychiatric and somatic outcomes when compared to unexposed periods.

**Methods**

**Study design**

We will apply the self-controlled case series (SCCS) study design in the proposed study.^25^ The analysis will be conducted on a population of individuals who had experienced both the outcome(s) and exposure of interest, which will be prescription of gabapentinoids. Incidence rate ratios (IRRs) will be calculated by comparing the event rates during periods of gabapentinoid exposure to the rates during all other observed time periods, in other words without gabapentinoid exposure. This will be accomplished using conditional Poisson regression.

This design has a significant advantage over the classical cohort design as included participants will act as their own control and it consequently addresses all potential time-invariant confounding effects that vary between individuals, such as genetic factors and socioeconomic factors.^26^ Additionally, we will account for time-varying factors, such as age and season, which can influence the prescription of gabapentinoids. We will also adjust for the concurrent use of other medications as time-varying factors. Sub-group analysis will also be performed for patients who suffer from mental illnesses, type 2 diabetes or cardiovascular diseases.

This method can investigate associations between acute outcomes and transient exposures^26^ and has been used extensively in literatures studying the safety of psychotropic medications and outcomes such as suicide attempts, self-harm and risk of seizure.^20,27-29^

**Setting**

UK primary and secondary care.

**Data source**

Clinical Practice Research Datalink (CPRD) Aurum database, linked to Hospital Episode Statistics (HES) and Office for National Statistics (ONS) Death Registrations.

**Study population**

All Individuals aged 18 or above who received at least one prescription for gabapentinoids (BNF chapter 4.8.1) and received at least one diagnosis of any of the outcomes of interest listed above between 1^st^ January, 2010 and 31^st^ December, 2020 in the CPRD, HES, and ONS Death Registration Data will be eligible for inclusion.

*Observation period*

The observation period will begin on 1^st^ January, 2010 or 18^th^ birthday of the individual or when the patient first join the GP practice (whichever was later) and end on 31^st^ December, 2020 or date of registered death or when the GP practice stops recording healthcare data for that patient (whichever was earlier).

*Exclusion criteria*

Individuals meeting any of the following criteria will be censored:

- Individuals who experienced any of the study outcomes before the observation period will be excluded.
- Patients who have a diagnosis of epilepsy.
- Patients with diagnosed cancer.
- Patients with missing information on date of birth and sex.

**Exposures**

Prescriptions of gabapentin (Anatomical Therapeutic Chemical code N03AX12) or pregabalin (N03AX16) will be identified in CPRD. The exposed period is defined as the time receiving the medication, calculated with the prescription information in CPRD (daily dosages and quantity prescribed).

The index date is defined as the first date of prescription of gabapentinoid medication. We will calculate the length of exposure using information on prescription date and prescribed days in the database. Patients will contribute to consecutive exposure risk periods while they were continuously exposed to the drug.

**Outcomes**

All-cause drug poisoning, stratified in accidental poisoning and intentional self-poisoning

**Covariates**

Time-varying confounders will include age, seasons, and use of concurrent medications.

Confounding by indication and time invariant factors such as genetic factors, individual vulnerability or psychiatric history are accounted by SCCS as it enables control for both known and unknown confounders that are specific to individual participants and do not vary over time. Each study participant will be used as their own control in this study design.

**Selection of comparison group**

Included patients will serve as their own control in this study design.^26^ Eligibility will be assessed based on the inclusion and exclusion criteria outlined in the section “Definition of the Study population”. The major advantage of SCCS over the classic design is that it controls for potential effects of measured and unmeasured time-invariant confounders that vary between individuals (i.e. genetic factors, disease severity, and socioeconomic factors).

A 90 days pre-exposure period will be included into the study. Calculation of the IRR during this period of time will be performed to evaluate whether outcomes of interest were caused by the conditions that gabapentinoids prescribed for instead of the use of gabapentinoids, which in turn may introduce bias into the risk estimate during treatment.

**Sample size consideration**

With an estimated IRR=1.5, n=484 is required with 5% level of significance and 80% power.^30^ The above sample sizes will be enough to study the outcomes with an incidence as low as 0.1%.

**Feasibility count**

The preliminary count in the CPRD contains records of 778,080 and 419,387 individuals with prescriptions of gabapentin and pregabalin, respectively.^31^ Together, more than 1,197,000 patients within CPRD will be eligible for the proposed study. As patients who have taken gabapentinoids will be subjects of interest, almost 1.2 million patients included in the database will be eligible for the overall cohort.

Previous study shows that 1 in 15 has attempted suicide in the UK.^32^ That will be equivalent to 80,000 patients within the cohort having this outcome of interest.

Another previous study shows that 8.9%, 6.3% and 36.7% of patients had unintentional overdose, road traffic incident and head/ body injuries, if they were on gabapentinoids, which will be equivalent to 106,000, 75,600 and 440,400 patients, respectively.^20^

**Statistical Analysis**

Gabapentinoid usage is diverse with multiple indications. It can be prescribed for neuropathic pain, fibromyalgia, postherpetic neuralgia, restless legs syndrome, generalised anxiety disorder, menopausal symptoms, complications of multiple sclerosis, insomnia and bipolar disorder.^2,13-16,19^ There is no other way to estimate their effects for a particular group of patient without using a stratified analysis. SCCS is a method of estimation that focuses on individuals and does not necessitate separate control groups.^33^ As a result, it is considered self-controlled, effectively eliminating the influence of time-invariant factors and we should not include time-invariant factors into the SCCS analysis.

In our study, we will determine the duration of exposure during the study period and compute the incidence rates for the specific outcomes of interest within each exposure risk period. To assess the association between gabapentinoid use and the risk of these outcomes, we will employ conditional Poisson regression. Conditional Poisson regression analysis will enable us to calculate incidence rate ratios (IRRs) by comparing the periods of exposure to gabapentinoids with unexposed periods, adjusted for age in 1-year bands, seasonal effect, and other concurrent medications. Adjusted IRRs, along with their corresponding 95% confidence intervals (CIs), will be estimated for the overall exposure as well as for each predefined exposure risk period. All statistical analyses will be conducted with a significance level of 5%. The calculated IRRs will be stratified by age, sex, ethnicity, types of gabapentinoids, dosage levels and pre-existing comorbidities.

In additional analyses, the interaction between gabapentinoids and opioids or benzodiazepines by comparing the incidence of all-cause drug poisoning across combined exposure windows with periods unexposed to either medication will be examined. Gabapentin-only compared with pregabalin-only treatment periods among individuals who received both drugs during follow-up will also be evaluated.

A series of sensitivity analyses will be performed, including a spline-based self-controlled case series analysis, exclusion of patients who died within 6 months of the event, starting follow-up from the first neuropathic or chronic pain diagnosis, restricting to individuals with at least two gabapentinoid prescriptions, varying the length of the pre-treatment period, adjusting only for age and season, extending treatment periods. Self-controlled case series extensions analyses will also be conducted.^34^ A negative control analysis will be performed to explore residual confounding. An additional case-case-time-control analysis^35^ is to validate the main findings, in which the 30 days before the event served as the hazard period and were compared with four randomly selected 30-day reference periods 61 to 180 days earlier, future cases occurring 180 to 360 days later were matched by age, sex, and ethnicity, and adjusted odds ratios (aORs) were estimated using conditional logistic regression. A two-sided significance level of 5% was used for all analyses.

For data analysis, we will utilise either the Statistical Analysis System (SAS) or R programming software.

**Missing data**

In cases where the prescription end date is not available, we will utilise daily dosages and the quantity prescribed to calculate the duration of treatment. If this information is missing, we will impute median values for exposure duration. It is important to note that missing data on baseline characteristics will not impact the analysis because the SCCS design uses each patient as their own control. Therefore, any potential differences in baseline characteristics are inherently accounted for within the study design.

**References**

1. Goodman CW, Brett AS. Gabapentin and Pregabalin for Pain - Is Increased Prescribing a Cause for Concern? *N Engl J Med* 2017; **377**(5): 411-4.

2. Goodman CW, Brett AS. A Clinical Overview of Off-label Use of Gabapentinoid Drugs. *JAMA Intern Med* 2019; **179**(5): 695-701.

3. Calandre EP, Rico-Villademoros F, Slim M. Alpha(2)delta ligands, gabapentin, pregabalin and mirogabalin: a review of their clinical pharmacology and therapeutic use. *Expert Rev Neurother* 2016; **16**(11): 1263-77.

4. Taylor CP, Angelotti T, Fauman E. Pharmacology and mechanism of action of pregabalin: the calcium channel alpha2-delta (alpha2-delta) subunit as a target for antiepileptic drug discovery. *Epilepsy Res* 2007; **73**(2): 137-50.

5. Sills GJ. The mechanisms of action of gabapentin and pregabalin. *Curr Opin Pharmacol* 2006; **6**(1): 108-13.

6. Uchitel OD, Di Guilmi MN, Urbano FJ, Gonzalez-Inchauspe C. Acute modulation of calcium currents and synaptic transmission by gabapentinoids. *Channels (Austin)* 2010; **4**(6): 490-6.

7. Hendrich J, Van Minh AT, Heblich F, et al. Pharmacological disruption of calcium channel trafficking by the alpha2delta ligand gabapentin. *Proc Natl Acad Sci U S A* 2008; **105**(9): 3628-33.

8. Fink K, Dooley DJ, Meder WP, et al. Inhibition of neuronal Ca(2+) influx by gabapentin and pregabalin in the human neocortex. *Neuropharmacology* 2002; **42**(2): 229-36.

9. Catterall WA, Few AP. Calcium channel regulation and presynaptic plasticity. *Neuron* 2008; **59**(6): 882-901.

10. Bers DM, Pogwizd SM, Schlotthauer K. Upregulated Na/Ca exchange is involved in both contractile dysfunction and arrhythmogenesis in heart failure. *Basic Res Cardiol* 2002; **97 Suppl 1**: I36-42.

11. Amberg GC, Navedo MF. Calcium dynamics in vascular smooth muscle. *Microcirculation* 2013; **20**(4): 281-9.

12. Yang SN, Berggren PO. The role of voltage-gated calcium channels in pancreatic beta-cell physiology and pathophysiology. *Endocr Rev* 2006; **27**(6): 621-76.

13. Joint Formulary Committee. Gabapentin. British National Formulary: Gabapentin London: BMJ Group and Pharmaceutical Press; 2022.

14. Joint Formulary Committee. Pregabalin. British National Formulary: Pregabalin London: BMJ Group and Pharmaceutical Press; 2022.

15. Royal Pharmaceutical Society of Great Britian. Gabapentin. Martindale: the complete drug reference - Gabapentin. London: Pharmaceutical Press; 2022.

16. Royal Pharmaceutical Society of Great Britian. Pregabalin. Martindale: the complete drug reference - Pregabalin. London: Pharmaceutical Press; 2022.

17. Montastruc F, Loo SY, Renoux C. Trends in First Gabapentin and Pregabalin Prescriptions in Primary Care in the United Kingdom, 1993-2017. *JAMA* 2018; **320**(20): 2149-51.

18. Kesselheim AS, Darby D, Studdert DM, Glynn R, Levin R, Avorn J. False Claims Act prosecution did not deter off-label drug use in the case of neurontin. *Health Aff (Millwood)* 2011; **30**(12): 2318-27.

19. Hong JSW, Atkinson LZ, Al-Juffali N, et al. Gabapentin and pregabalin in bipolar disorder, anxiety states, and insomnia: Systematic review, meta-analysis, and rationale. *Mol Psychiatry* 2022; **27**(3): 1339-49.

20. Molero Y, Larsson H, D'Onofrio BM, Sharp DJ, Fazel S. Associations between gabapentinoids and suicidal behaviour, unintentional overdoses, injuries, road traffic incidents, and violent crime: population based cohort study in Sweden. *BMJ* 2019; **365**: l2147.

21. Mula M, Hesdorffer DC. Suicidal behavior and antiepileptic drugs in epilepsy: analysis of the emerging evidence. *Drug Healthc Patient Saf* 2011; **3**: 15-20.

22. Mula M, Sander JW. Negative effects of antiepileptic drugs on mood in patients with epilepsy. *Drug Saf* 2007; **30**(7): 555-67.

23. Tallian KB, Nahata MC, Lo W, Tsao CY. Gabapentin associated with aggressive behavior in pediatric patients with seizures. *Epilepsia* 1996; **37**(5): 501-2.

24. Pinninti NR, Mahajan DS. Gabapentin-associated aggression. *J Neuropsychiatry Clin Neurosci* 2001; **13**(3): 424.

25. Lao KS, Chui CS, Man KK, Lau WC, Chan EW, Wong IC. Medication safety research by observational study design. *Int J Clin Pharm* 2016; **38**(3): 676-84.

26. Whitaker HJ, Farrington CP, Spiessens B, Musonda P. Tutorial in biostatistics: the self-controlled case series method. *Stat Med* 2006; **25**(10): 1768-97.

27. Man KKC, Coghill D, Chan EW, et al. Association of Risk of Suicide Attempts With Methylphenidate Treatment. *JAMA Psychiatry Journal Translated Name JAMA Psychiatry* 2017; **74**(10): 1048-55.

28. Chai Y, Luo H, Man KKC, et al. Antidepressant use and risk of self-harm among people aged 40 years or older: A population-based cohort and self-controlled case series study. *Lancet Reg Health West Pac* 2022; **27**: 100557.

29. Man KKC, Lau WCY, Coghill D, et al. Association between methylphenidate treatment and risk of seizure: a population-based, self-controlled case-series study. *Lancet Child Adolesc Health* 2020; **4**(6): 435-43.

30. Musonda P, Farrington CP, Whitaker HJ. Sample sizes for self-controlled case series studies. *Stat Med* 2006; **25**(15): 2618-31.

31. Julie Ashworth RB, Sara Muller, James Bailey, Toby Helliwell, Sarah A. Harrisson, Rebecca Whittle, Christian D. Mallen Trends in gabapentinoid prescribing in UK primary care using the Clinical Practice Research Datalink: an observational study. *The Lancet Regional Health - Europe* 2023.

32. McManus S BP, Jenkins R, Brugha T. . Mental health and wellbeing in England: Adult psychiatric morbidity survey 2014. England: NHS DIgital; 2016.

33. Petersen I, Douglas I, Whitaker H. Self controlled case series methods: an alternative to standard epidemiological study designs. *BMJ* 2016; **354**: i4515.

34. Farrington CP, Whitaker HJ, Hocine MN. Case series analysis for censored, perturbed, or curtailed post-event exposures. *Biostatistics* 2009; **10**(1): 3-16.

35. Wang S, Linkletter C, Maclure M, et al. Future cases as present controls to adjust for exposure trend bias in case-only studies. *Epidemiology* 2011; **22**(4): 568-74.
