# Supplementary material for: Association between gabapentinoid treatment, concurrent use with opioid or benzodiazepine and the risk of drug poisoning: A self-controlled case series study
Source: PLoS Med. 2026 Apr 16;23(4):e1005035. doi: 10.1371/journal.pmed.1005035 (PMC13086301; doi:10.1371/journal.pmed.1005035)
Supplement: Attachment — Submitted filename: Response to Editors and reviewers.docx [file pmed.1005035.s042.docx]

**Response to comments from Editors and Reviewers**

Requests from Editors:

GENERAL

* Please confirm that your title complies with PLOS Medicine's style. Your title must be nondeclarative and not a question. It should begin with main concept if possible. "Effect of" should be used only if causality can be inferred, i.e., for an RCT. Please place the study design ("A randomized controlled trial," "A retrospective study," "A modelling study," etc.) in the subtitle (ie, after a colon).

* Title: we suggest to change the title to: “Association between gabapentinoid treatment, concurrent use with opioid or benzodiazepine and the risk of drug poisoning – a self-controlled case series study”.

Response: We would like to thank the editor for the suggestion. We have now revised the study title as suggested.

“*Association between gabapentinoid treatment, concurrent use with opioid or benzodiazepine and the risk of drug poisoning – a self-controlled case series study*” (P.1, Line 2-3)

* Please ensure that all abbreviations are defined at first use throughout the text.
Response: We would like to thank the editor for the reminder. We have now carefully checked the manuscript to ensure that all abbreviations are defined at first mention throughout the text.

* Please confirm that all numbers presented in the abstract are present and identical to numbers presented in the main manuscript text.

Response: We would like to thank the editor for the reminder. We have double-checked the manuscript and confirm that all numbers reported in the Abstract are presented consistently and identically in the main text.

* Please review your text for claims of novelty or primacy (e.g. 'for the first time') and remove this language. In addition, please check that any use of statistical terms (such as trend or significant) are supported by the data, and if not please remove them.
Response: We would like to thank the editor for the reminder. We have now removed wording indicating that this is the first within-individual study to examine the association of interest, and we have also removed the term “trend” from the manuscript. The use of the term “significant” has been retained where it is supported by the data.

“The spline-based SCCS analysis demonstrates a consistent risk pattern with an increasing risk before the start of treatment, followed by a further decline after treatment initiation (S6 Fig).” (P.17, Line 349-351)

“This study employed a within-individual design investigating the risk of all-cause drug poisoning of gabapentinoids with the adjustment of age, season and concomitant medications.” (P.20, Line 423-424)

* Please remove the 'conclusions' subheading from the discussion. Please also remove any other subheadings from the discussion.
Response: We have now removed the “Conclusion” subheading from the Discussion and ensured that there are no remaining subheadings within that section.

* Statistical reporting: Thank you for formatting your manuscript to fit with PLOS Medicine guidelines. However, the tables have not been adjusted. Please revise the tables, and check the whole manuscript.
- Please report statistical information as follows to improve clarity for the reader ""22% (95% CI [13,28]; p</=)"".
- Please separate upper and lower bounds with commas instead of hyphens as the latter can be confused with reporting of negative values.
- Please repeat statistical definitions (HR, CI etc.) for each set of parentheses.
Response: We would like to thank the editor for the reminder. We have now revised the presentation of confidence intervals in all tables, including those in the Supplementary Materials, to align with PLOS Medicine style by using commas to separate the lower and upper bounds. We have also ensured that statistical definitions are repeated for each set of parentheses.

DATA AVAILABILITY STATEMENT
* DAS: Thank you for providing the code. However, I can’t find the page, could you please confirm the URL? Furthermore, We strongly recommend that all code be deposited in a permanent, public repository that issues citable digital object identifiers (DOI) or other persistent identifiers, such as Zenodo.
Response: We confirm that the URL included in the Data Availability Statement is correct. It currently appears as “Not Found” because the repository has not yet been made public. Once the paper is published, the link will be made publicly accessible. We also confirm that the GitHub page provided is intended to serve as a permanent public repository and has been linked to Zenodo.

*Can you verify that the HES and ONS data are also obtained from CPRD? If not, please add how these datasets can be accessed in the data availability statement.
Response: Yes, HES and ONS data are also obtained from CPRD.

ABSTRACT

* In the last sentence of the Abstract Methods and Findings section, please describe the main limitation(s) of the study's methodology.
Response: We would like to thank the editor for the suggestion. We have now included the major limitation at the end of the Methods and Findings section.

“*The SCCS approach cannot completely exclude the effect of unmeasured time-varying confounders, such as transient changes in socioeconomic status, major life events, or illicit drug use, although the negative control analysis did not suggest meaningful residual confounding*.” (P.4, Line 71-74)

* In the abstract, please include the important dependent variables that are adjusted for in the analyses.
Response: We would like to thank the editor for the suggestion. We have now included a sentence stating the variables adjusted for in the analyses.

“*All analyses have adjusted for key time-varying confounders, including age, season, and concomitant use of opioids, antiseizure medications, psychotropic medications, and non-steroidal anti-inflammatory drugs (NSAIDs)*.” (P.3, Line 59-61)

* First sentence abstract-background: please check whether this statement is correct.
Response: We would like to thank the editor for the query. Yes, this is correct, as shown in our previous study using worldwide drug consumption data [1]. Global consumption of gabapentinoids has increased by 4-fold in the span of 11 years.

*line 71: typo in Gabapentinoid, please correct.

Response: This has been corrected.


AUTHOR SUMMARY

* In the author summary, please revise formatting and ensure you use bullet points.
Response: We have now changed the formatting into bullet points.

* "why was this study done": consider adding a few words what gabapentinoids are used for.
Response: We would like to thank the editor for this suggestion. We have revised the first sentence of “why was this study done” to include the indications of gabapentinoids.

“•	*Use of gabapentinoids, which are indicated for neuropathic pain, epilepsy, and generalised anxiety disorder, and are also used off label for fibromyalgia, sleep disorders, and other chronic pain conditions, has increased substantially over the past decade, and there are growing concerns that they may contribute to drug poisoning and overdose, particularly when combined with opioids or benzodiazepines*.” (P.5, Line 83-87)

*“What did the researchers do and find: first point”: please rewrite this in laymen's terms. Consider adding a description of the data used, rather than (or in addition to) the names of the databases. Also spell out the SCCS acronym, and consider adding a sentence of explanation on this study-type.
Response: We would like to thank the editor for the recommendation. We have now revised the first point under “What did the researchers do and find” to describe the data sources in lay terms, spell out the SCCS acronym, and add a sentence explaining the main feature of this study design.

“•	*The authors used linked, anonymised health records from general practices, hospitals, and death registrations in England to study 16,827 adults prescribed gabapentinoids who had experienced a first drug poisoning event. The study applied a self-controlled case series design, which compares the risk during different time periods within the same person so that each person acts as their own control.*” (P.5, Line 98-102)

OBSERVATIONAL, COHORT, CROSS-SECTIONAL, AND CASE CONTROL STUDIES

* Did your study have a prospective protocol or analysis plan? Please state this (either way) early in the Methods section.
a) If a prospective analysis plan (from your funding proposal, IRB or other ethics committee submission, study protocol, or other planning document written before analyzing the data) was used in designing the study, please include the relevant prospectively written document with your revised manuscript as a Supporting Information file to be published alongside your study, and cite it in the Methods section. A legend for this file should be included at the end of your manuscript.
b) If no such document exists, please make sure that the Methods section transparently describes when analyses were planned, and when/why any data-driven changes to analyses took place.
c) In either case, changes in the analysis-- including those made in response to peer review comments-- should be identified as such in the Methods section of the paper, with rationale.
Response: We thank the Editor for the recommendation. We have now included a statement describing the pre-approved, prospectively registered protocol with CPRD. We have also included an additional supplementary materials to include the protocol.

“*Analyses were performed in accordance with the pre-registered study protocol approved by the Independent Scientific Advisory Committee of CPRD (S1 Protocol). A post hoc analysis in which gabapentinoid prescriptions issued 90 days or less apart were not combined was additionally conducted in response to reviewer comments.*” (P.15, Line 305-308)

FIGURES AND TABLES:

* When a p value is given, please specify the statistical test used to determine it in the legend.

Response: We thank the Editor for this suggestion. We have now added wording to the related table legends, including the ones in the supplementary materials, to specify the statistical test used to derive the p values:

“*P* values were obtained from two-sided Wald tests.”

* Please consider if moving some of the figures shared in the supplementary information into the main text would aid the reader. Specifically, we would suggest moving figure S1 to the main text.

Response: Thank you for the suggestion. We have now moved Figure S1 into the main manuscript, where it is now presented as Figure 1, and have rearranged the numbering of the remaining figures accordingly.

Comments from Academic Editor:

The authors have responded well to the comments. Reviewer 2 is concerns about possible biological mechanisms, which is not an aim of the study. They are also concerns about time-varying confounders, which is an inherent limitation to the design used, and the authors have used reasonable adjustments to address this. the use of negative controls is a further strength of the paper.

Response: Thank you very much for the positive and thoughtful assessment of our revised manuscript. We appreciate the recognition that we have addressed the reviewer comments appropriately. We agree that detailed biological mechanisms are beyond the scope of the present study, and we are grateful for the acknowledgement that the inclusion of a negative control analysis further strengthen the paper.

Comments from Reviewers:

Reviewer #1:
Thanks for the revised manuscript and responses to my original review. The revised manuscript and responses to my questions have resolved my original questions.

I wasn't clear in my original review about the finding for the negative control - I absolutely agree with the authors that the results for the sensitivity analysis strengthens the manuscript.

I am comfortable with the authors choice for a 90-day gap - this is time-period is commonly selected in pharmacoepidemiology studies of other medicines, and the justification provided in response (supported by the results of the sensitivity analysis) is sound.

Response: We would like to thank the supportive comments from the reviewer. We are grateful for the recognition that the sensitivity analyses strengthen the manuscript. We also thank you for noting that the 90-day gap is a reasonable, and that our justification was adequately supported.


Reviewer #2: Review for PMEDICINE-D-25-03057R2

While the authors have attempted to address the questions, major concerns remain as follows:

*       Mechanisms between gabapentinoid treatment and all-cause drug poisoning remain unclear, which is also pointed out by the authors' response-- "the actual biological mechanism is yet to be understood". The authors provided new data that among the identified 16,827 patients with all-cause drug poisonings in the study, most of the patients had multiple poisoning diagnoses on the same day.  This raises another big question: how likely is it that the use of gabapentin treatment could be so detrimental, leading to multiple types of all-cause drug poisoning being diagnosed for the same person at the same time? The underlying biological or clinical mechanisms are unlikely to be plausible. The rationale for studying all-cause drug poisoning remains not justified.
Response: We thank the reviewer for raising this important point regarding the biological mechanisms underlying the observed association. We agree that the precise biological mechanisms linking gabapentinoid treatment and all-cause drug poisoning cannot be established from the present study, and we did not intend to make mechanistic inferences, as also acknowledged by the Academic Editor. The aim of this study was to examine the association between gabapentinoid treatment and the risk of all-cause drug poisoning in routine clinical practice, rather than to determine the exact biological pathway.

With regards to the observation that many patients had more than one poisoning diagnosis code recorded on the same day, we would like to clarify that this does not imply that gabapentinoids caused multiple distinct poisoning mechanisms or that patients were necessarily poisoned by multiple agents simultaneously. Rather, as shown in the table provided in our previous response, a single poisoning episode may receive more than one ICD-10 code to capture the clinical presentation, suspected substance involvement, and the recorded intent of the event, hence, we adopted the definition of all-cause drug poisoning in this study as explained in the previous round of response.

In addition, we are not suggesting that gabapentinoids are inherently “detrimental” or that the observed associations are necessarily driven by a direct toxic effect of gabapentinoids alone. Gabapentinoids are known to have central nervous system depressant effects, and regulatory agencies have raised concerns regarding misuse, overdose, and respiratory depression, particularly when these medicines are used concomitantly with other central nervous system depressants such as opioids or benzodiazepines [1-5]. At the same time, we would also draw the reviewer’s attention to the elevated risk of drug poisoning observed during the period before treatment initiation, which has not been examined in most previous studies. The highest risk identified during this pre-treatment period suggests that the underlying clinical conditions for which gabapentinoids are prescribed may themselves be an important contributor to the increased risk of all-cause drug poisoning observed in this study. However, although the risk was attenuated after treatment initiation, it did not return to the reference baseline level observed during periods when individuals were unaffected by these underlying conditions. This pattern suggests that, in addition to the contribution of the underlying clinical condition, gabapentinoid treatment itself may also be associated with an increased risk of drug poisoning. We therefore do not imply that gabapentinoids alone causally increase the risk of poisoning, and we have been careful throughout the manuscript to interpret our findings as associations rather than proof of causation.

*       The conclusion—"The results suggest that gabapentinod is associated with an increased risk of drug poisoning" — remains problematic and overstated. The limitations of data and design cannot tease out whether the occurrence of all-cause drug poisoning is mainly because of gabapentinoid treatment or is due to the underlying serious conditions (e.g., depression), which pain condition (potentially treated by gabapentin) is also prevalently co-occurred with. In other words, gabapentinoid may be a "good" drug and used to treat co-morbidities or symptoms of major conditions that cause all-cause drug poisoning. The points are also mentioned in the response by authors, "The prescribing of gabapentinoids is likely to reflect a clinical response to worsening underlying conditions or an attempt to minimise the risk of drug poisoning by switching from alternative treatments. However, our data do not allow us to determine the precise clinical reasons for the heightened risk observed in
the 90 days preceding gabapentinoid initiation, and further research is warranted." (P.18, Line 383 to 388).

Response: We thank the reviewer for this comment. We agree that the present study cannot disentangle whether the increased risk of all-cause drug poisoning is causally attributable to gabapentinoid treatment itself, the underlying clinical conditions for which gabapentinoids are prescribed, or both. The wording used in this manuscript is not intended to imply causality, but rather to describe the temporal association observed in routine clinical practice using a self-controlled case series design.

In addition, we respectfully note that the conclusion reflects the pattern observed in the results. Although the highest risk was seen in the pre-treatment period, suggesting that the underlying clinical conditions may themselves contribute to the increased risk of poisoning, the risk during gabapentinoid treatment, particularly at treatment initiation, remained elevated relative to the baseline reference period rather than returning fully to null as explained in the response to the previous point. This pattern suggests that, in addition to the contribution of the underlying condition, gabapentinoid treatment periods were also associated with an increased risk of drug poisoning in this study. We have therefore used cautious, non-causal wording, namely that gabapentinoid treatment was “associated” with an increased risk, rather than stating that gabapentinoids alone caused the increased risk of poisoning.

We also agree that worsening underlying symptoms or comorbidities may partly explain the elevated risk before treatment initiation. This is one reason why we highlighted the markedly increased risk during the 90 days preceding treatment start and explicitly noted in the Discussion section that our data do not allow us to determine the precise clinical reasons for this pattern. We believe this finding is important because it indicates that the increased risk observed cannot be attributed solely to gabapentinoid exposure itself.

*       Study participants remain concerning because CPRD data are not linked to diagnostic codes (P.20 to 21, lines 433 to 435)." The current selected study participants with gabapentinoid consist of a mixed group of patients who received the drug for treating various clinical conditions, complicating the study findings and interpretations.

Response: We thank the reviewer for this important comment. The primary aim of this study was to examine whether gabapentinoid treatment is associated with an increased risk of drug poisoning, given the substantial rise in gabapentinoid use and their broad range of clinical indications [6]. This question is of particular public health relevance in light of recent regulatory concerns, including the MHRA Drug Safety Update highlighting the risks of dependence and misuse associated with gabapentinoids [7]. Our findings add to this emerging safety literature by showing an increased risk of drug poisoning during gabapentinoid treatment, and the SCCS design provides a robust framework to investigate this association while controlling for time-invariant between individual confounders.

At the same time, it is acknowledged that this study cannot address every aspect of the issue within a single analysis, including the underlying biological mechanisms or indication-specific pathways that may contribute to the observed association. Further research is therefore warranted, as noted in the manuscript.

We agree that patients prescribed gabapentinoids represent a clinically heterogeneous population, and that treatment indications are not always fully captured in CPRD primary care records, as is common in many studies using routine healthcare data. This is an inherent limitation of database research, where formal prescribing indications are not consistently recorded, and this limitation has been acknowledged in the manuscript.

To further explore whether the association between gabapentinoid treatment and drug poisoning differed across clinically relevant patient groups, we conducted a series of prespecified subgroup analyses among individuals with mental health-related comorbidities that may themselves be associated with an elevated risk of drug poisoning [8, 9]. Specifically, we stratified the analyses by bipolar disorder or mania, depression, anxiety, schizophrenia, other psychosis, insomnia, and substance misuse (Table S17). These analyses were undertaken to assess whether the observed association differed materially across clinically relevant subgroups with varying baseline vulnerability to drug poisoning.

To better emphasise the subgroup analyses that we have performed, we have expanded the limitation statement added in the previous round as follow:

“*Fifth, as prescribing records in CPRD are not linked to diagnostic codes, we were unable to identify the specific clinical indications for which gabapentinoids were prescribed at each treatment episode. Nevertheless, subgroup analyses according to underlying mental health comorbidities (Table S17) yielded results that were broadly consistent with the main analysis.*” (P.22, Line 452-455)

*       The limitation that CPRD data are not linked to diagnostic codes likely creates potential disease indication for treatment or disease severity for the findings, a major concern that has been brought up.  In this response, the authors argued that "In the statistical analysis model, we explicitly adjusted for time-varying proxies of disease severity and clinical instability, especially concomitant prescriptions for opioids, antiseizure medications, hypnotics and anxiolytics, benzodiazepines, antidepressants, antipsychotics, lithium and NSAIDs."  The statement is not supported by any references. What does clinical instability mean? In clinical settings, the use of these medications is rarely considered to be a valid indicator of clinical severity and clinical instability of the underlying treated conditions. The assertion is overstated.

Response: We thank the reviewer for this comment and appreciate the opportunity to clarify our rationale for adjusting for time-varying co-medications in this study. First, previous studies have used treatment patterns as proxies for disease severity, particularly in settings where the severity of the wide range of conditions, for which gabapentinoids may be prescribed, is not consistently captured in healthcare databases [10-13], In addition, Methodological literature [14] has also recognised that medications can serve as proxies for diagnoses, risk factors, and disease severity when these are not fully captured in structured clinical records.

Our intention in including these time-varying co-medications was therefore to use them as pragmatic markers of changes in treatment complexity, comorbidity management, and clinical status over time, all of which could confound the association between gabapentinoid treatment and drug poisoning. In the context of this study, increased prescribing of the included co-medications may indicate worsening pain, sleep disturbance, anxiety, psychiatric morbidity, substance-related problems, or the need for more intensive symptom management, even if the direct severity of the underlying condition is not fully captured in the database.

Reviewer #3: Thank you for thoroughly addressing all the reviewer comments and offering additional analyses and insights to strengthen confidence in your work.

Response: Thank you for the reviewer’s positive feedback. We appreciate the careful review and are pleased that our revisions and additional analyses have addressed the comments and strengthened the manuscript.

**References**

1.	Administration UFaD. FDA warns about serious breathing problems with seizure and nerve pain medicines gabapentin (Neurontin, Gralise, Horizant) and pregabalin (Lyrica, Lyrica CR) 2019 [cited 2023 19/05/2023]. Available from: <https://www.fda.gov/drugs/drug-safety-and-availability/fda-warns-about-serious-breathing-problems-seizure-and-nerve-pain-medicines-gabapentin-neurontin>.

2.	Limited UU. Lyrica 100 mg hard capsules SmPC 2022 [cited 2023 01/09/2023]. Available from: <https://www.medicines.org.uk/emc/product/10303/smpc>.

3.	Limited UU. Neurontin 100mg Hard Capsules SmPC 2023 [cited 2023 01/09/2023]. Available from: <https://www.medicines.org.uk/emc/product/158/smpc>.

4.	Medicines and Healthcare products Regulatory Agency. Pregabalin (Lyrica), gabapentin (Neurontin) and risk of abuse and dependence: new scheduling requirements from 1 April, 2019 [updated 16 April 2019]. Available from: <https://www.gov.uk/drug-safety-update/pregabalin-lyrica-gabapentin-neurontin-and-risk-of-abuse-and-dependence-new-scheduling-requirements-from-1-april>.

5.	Therapeutic Goods Administration. Pregabalin and gabapentin, Australia: Australia Government - Department of Health and Aged Care; 2021 [updated 01 February]. Available from: <https://www.tga.gov.au/news/safety-alerts/pregabalin-and-gabapentin>.

6.	Chan AYL, Yuen ASC, Tsai DHT, Lau WCY, Jani YH, Hsia Y, et al. Gabapentinoid consumption in 65 countries and regions from 2008 to 2018: a longitudinal trend study. Nat Commun. 2023;14(1):5005. Epub 20230817. doi: 10.1038/s41467-023-40637-8. PubMed PMID: 37591833; PubMed Central PMCID: PMCPMC10435503.

7.	Agency MaHpR. Improving Information Supplied with Gabapentinoids (Pregabalin/Gabapentin), Benzodiazepines and Z-Drugs 2026 [cited 2026 11 March 2026]. Available from: <https://www.gov.uk/drug-safety-update/improving-information-supplied-with-gabapentinoids-pregabalin-slash-gabapentin-benzodiazepines-and-z-drugs>.

8.	van Draanen J, Tsang C, Mitra S, Phuong V, Murakami A, Karamouzian M, et al. Mental disorder and opioid overdose: a systematic review. Soc Psychiatry Psychiatr Epidemiol. 2022;57(4):647-71. Epub 20211118. doi: 10.1007/s00127-021-02199-2. PubMed PMID: 34796369; PubMed Central PMCID: PMCPMC8601097.

9.	Walter F, Carr MJ, Mok PLH, Antonsen S, Pedersen CB, Shaw J, et al. Suicide Methods and Specific Types of Accidental Death and Fatal Poisoning Among Discharged Psychiatric Patients: A National Cohort Study. J Clin Psychiatry. 2018;79(6). Epub 20181002. doi: 10.4088/JCP.17m11809. PubMed PMID: 30289629; PubMed Central PMCID: PMCPMC6193537.

10.	Yuen ASC, Chen B, Chan AYL, Hayes JF, Osborn DPJ, Besag FMC, et al. Use of gabapentinoid treatment and the risk of self-harm: population based self-controlled case series study. BMJ. 2025;389:e081627. Epub 20250430. doi: 10.1136/bmj-2024-081627. PubMed PMID: 40306941.

11.	Brauchli YB, Jick SS, Meier CR. Psoriasis and the risk of incident diabetes mellitus: a population-based study. Br J Dermatol. 2008;159(6):1331-7. Epub 20080906. doi: 10.1111/j.1365-2133.2008.08814.x. PubMed PMID: 18782318.

12.	Emeny RT, Chang CH, Skinner J, O'Malley AJ, Smith J, Chakraborti G, et al. Association of Receiving Multiple, Concurrent Fracture-Associated Drugs With Hip Fracture Risk. JAMA Netw Open. 2019;2(11):e1915348. Epub 20191101. doi: 10.1001/jamanetworkopen.2019.15348. PubMed PMID: 31722031; PubMed Central PMCID: PMCPMC6902800.

13.	Man KKC, Lau WCY, Coghill D, Besag FMC, Cross JH, Ip P, et al. Association between methylphenidate treatment and risk of seizure: a population-based, self-controlled case-series study. Lancet Child Adolesc Health. 2020;4(6):435-43. doi: 10.1016/S2352-4642(20)30100-0. PubMed PMID: 32450123.

14.	Norgaard M, Ehrenstein V, Vandenbroucke JP. Confounding in observational studies based on large health care databases: problems and potential solutions - a primer for the clinician. Clin Epidemiol. 2017;9:185-93. Epub 20170328. doi: 10.2147/CLEP.S129879. PubMed PMID: 28405173; PubMed Central PMCID: PMCPMC5378455.
