# Supplementary material for: Association between gabapentinoid treatment, concurrent use with opioid or benzodiazepine and the risk of drug poisoning: A self-controlled case series study
Source: PLoS Med. 2026 Apr 16;23(4):e1005035. doi: 10.1371/journal.pmed.1005035 (PMC13086301; doi:10.1371/journal.pmed.1005035)
Supplement: Attachment — Submitted filename: Response to Editors comments.docx [file pmed.1005035.s043.docx]

1) Thank you for stating that your GitHub page has been linked to Zenodo. Please also include the Zenodo URL in the Data Availability statement.

Response: We would like to thank the Editor for the reminder. We have now included the Zenodo URL/DOI for the corresponding GitHub repository in the Data Availability Statement.

“*The Zenodo URL is https://doi.org/10.5281/zenodo.19105047*.” (P.26-27, Line 552-553)

2) Thank you for making the requested changes to the author summary. However, the sentence for the first bullet point is now quite long. Please split this into 2 sentences for readability. We suggest:

“Use of gabapentinoids, which are indicated for neuropathic pain, epilepsy, and generalised anxiety disorder, and are also used off label for fibromyalgia, sleep disorders, and other chronic pain conditions, has increased substantially over the past decade. However, there are growing concerns that they may contribute to drug poisoning and overdose, particularly when combined with opioids or benzodiazepines.”

Response: We would like to thank the editor for the suggestion. We have now revised the sentence structure of the first point in the Author Summary, as shown below:

“•	*Use of gabapentinoids, which are indicated for neuropathic pain, epilepsy, and generalised anxiety disorder, and are also used off label for fibromyalgia, sleep disorders, and other chronic pain conditions, has increased substantially over the past decade. However, there are growing concerns that they may contribute to drug poisoning and overdose, particularly when combined with opioids or benzodiazepines.*” (P.5, Line 83-87)

Thank you for providing a thoughtful response to the remaining reviewer comments. However, whereas the rebuttal was extensive, we noted that little of this made it into the MS. Please add something of this rebuttal to the paper

3) in relating to the first point, maybe clarify further that the study is not aimed at outlining biological mechanisms.

Response: We would like to thank the editor for the recognition and suggestion. At the end of the Introduction, we have now explicitly clarified that the aim of this study was to examine the association between gabapentinoids and drug poisoning, rather than to investigate the underlying biological mechanisms of this association. In addition, we have included further text in the Exposures and Outcomes section to explain the rationale for adopting “all-cause drug poisoning” as the outcome definition, to better address the reviewer’s concern. We have also incorporated these additional revisions into the updated rebuttal letter.

“*The aim of this study is not to examine the underlying biological mechanisms linking gabapentinoid treatment to drug poisoning, but rather to evaluate their association in routine clinical practice.”* (P.9, Line 169-171)

*“To avoid differential misclassification across drug poisoning intent categories, we adopted a composite definition using combinations of ICD-10 drug-poisoning diagnosis codes. This approach is consistent with previous epidemiological studies investigating risk factors associated with drug poisoning [34-37].”* (P.12-13, Line 240-244)

4) in regarding to the second point, could you add some additional text to the limitation section?

Response: We would like to thank the editor for the suggestion. We have added further text to the final part of the limitations section to better address the reviewer’s concern and to highlight the need for future studies investigating the relationship between drug poisoning risk and the underlying clinical conditions associated with gabapentinoid prescribing. We have also included this revised text in the rebuttal letter.

“*In addition, although the SCCS design controls for all time-invariant confounders and we adjusted for a range of concomitant medications as time-varying covariates, our study cannot fully account for time-varying changes in the severity of the underlying clinical conditions that prompted gabapentinoid initiation. Worsening pain, psychiatric symptoms, or other changes in health status may have contributed to the observed risk of drug poisoning around treatment initiation, and further studies are warranted to investigate the underlying clinical factors of this pattern.*” (P.23, Line 469-475)

The third and fourth point do not need any further revision to the text.

Response: We have kept the third and fourth point unchanged.
