# Supplementary material for: Association between gabapentinoid treatment, concurrent use with opioid or benzodiazepine and the risk of drug poisoning: A self-controlled case series study
Source: PLoS Med. 2026 Apr 16;23(4):e1005035. doi: 10.1371/journal.pmed.1005035 (PMC13086301; doi:10.1371/journal.pmed.1005035)
Supplement: Attachment — Submitted filename: Reviewer comments response.docx [file pmed.1005035.s041.docx]

Reviewer #1: Thanks for the opportunity to read your manuscript. My role is statistical reviewer, so I have focused on the design, data, and analysis that are presented. I have put general comments first, followed by questions relevant to a specific section of the manuscript (with a page/line reference).

Response: We would like to thank the reviewer for the comments.

This manuscript estimates the association of treatment with gabapentinoids with drug poisoning, and whether there is an interaction between gabapentinoids and other drug classes. Data is from the UK CPRD, which links general practice data with hospital, prescription and mortality data. The self-controlled case series design was used to estimate the association between exposure and drug poisoning. Participants were selected who had a hospital record of drug poisoning between 2010 and 2020 and excluded if they had epilepsy or cancer, or prescribed gabapentinoid in the year before the start of the temporal window or had an event on the first day of the exposure or missing age or sex data. Exposure to gabapentinoid was derived from prescription duration, based on daily dose and quantity supplied. Gaps of coverage less than 90 days were considered contiguous. The period 90 days before gabapentinoid treatment was used as the 'control' portion of the case series, and several temporal windows up to 84 days after initiating treatment. Exposure to opioids and benzos was included as an exposure - to estimate the interaction between gabapentinoid an 18 level variable that combines the 3 opioid/benzo exposure periods and the 6 gabapentinoid periods was created. Poisson regression was used for the main analysis, to estimate incidence rates in the 'treatment' period compared to the control periods. This included an adjustment for age, and concomitant medications, and results presented stratified by key personal variables and drug information. Several sensitivity analyses were considered, including a spline-based approach to differences across the study temporal window, and a negative control analysis with food poisoning as an outcome. The rationale for all of these is explained well. I agree with the authors that the results of the sensitivity analyses are in line with the main results, except for the negative control which does not seem to be associated with gabapentinoid exposure. The limitations of the study are articulated well (e.g. unmeasured time-varying confounders).
Response: We thank the reviewer for the thorough summary of the method adopted in this study. We wish to clarify that it is expected not to have any association observed in the negative control analysis. A negative control outcome should be measured through similar mechanisms as the primary outcome but should not be causally affected by the exposure [1]. We therefore selected food poisoning because it is an acute poisoning-type event which shares similar pathways of health-care utilisation with drug poisoning. At the same time, there is no biologically plausible mechanism in which gabapentinoids would increase the risk of food poisoning, which is predominantly driven by exposure to contaminated food rather than medication use.

The absence of an association in our negative control analysis is therefore reassuring and suggests that our main findings are unlikely to be entirely explained by residual confounding.

There was extensive supplementary material included - this was a great help in reviewing the paper, thank you. I enjoyed reading this manuscript - there were only a few small clarifications I think the manuscript needs.

Response: Thank you for the positive comments. We are glad that the supplementary materials were helpful in your review of the paper.

As a non-expert in gabapentinoid treatment, I was unclear about the potential for non-intentional poisoning, there is some useful context about misuse, but an additional sentence or two that summarises non-intentional and intentional poisoning with gabapentinoid would be helpful.

Response: We thank the reviewer for highlighting the need to clarify the potential for non-intentional (accidental) poisoning related to gabapentinoid use. Previous studies have suggested potential association between gabapentinoid use and both intentional and unintentional poisoning separately [2, 3]. Although the actual mechanism is yet to be confirmed, it is clinically important to investigate risks related to both types of drug poisoning.

To better explain this distinction and place our study in context, we have added the following sentences to the Background section:

*“Gabapentinoid-related poisoning can include both intentional self-poisoning, typically occurring in the context of self-harm, and non-intentional events such as accidental ingestions and misuse [3, 4]. Some population-based and poison-centre studies report increased risks of suicidal behaviour and unintentional overdoses during gabapentinoid treatment in recent years [2, 3].” (P.7 to 8, Line 137 to 141)*

P7, L137. Were there any drug poisoning episodes identified from mortality data not recorded in the HES?

Response: We thank the reviewer for this insightful comment. In this study, all-cause drug poisoning was identified solely from diagnoses recorded in the HES database. Linked ONS mortality data were used only to ascertain vital status and to censor follow-up at death, not to define incident poisoning outcomes. Consequently, individuals whose drug poisoning event was recorded only in the ONS database as the cause of death, without any corresponding HES drug poisoning diagnosis before death, were not included in the analysis. By design, all individuals who died during follow-up had at least one HES-recorded drug-poisoning diagnosis prior to death, as such a diagnosis was required for cohort entry.

P7, L141. Were participants included if the drug poisoning occurred before they were 18?

Response: We thank the reviewer for this question. Individuals whose drug poisoning occurred exclusively before the age of 18 were not included in the cohort. Our study cohort was restricted to individuals who were aged ≥18 years and had at least one drug poisoning event during follow-up. Participants were therefore only included if their drug poisoning event occurred at age 18 or older.

P8, L155. What is the typical prescription period for gabapentinoid in the UK?

Response: Thank you for raising this question regarding the typical prescription period for gabapentinoids in the UK. In our study cohort, the median duration of individual gabapentinoid prescriptions was 28 days (interquartile range 7 to 28 days).

P10, L196. Stratifying by different types of outcomes technically means you have defined additional outcomes, rather than doing a sub-group analysis. This might be better explained these terms.

Response: We thank the reviewer for this helpful clarification. We agree that analysing accidental and intentional drug poisoning separately corresponds to using additional outcome definitions rather than conducting subgroup analyses. In the revised manuscript, we now describe these as separate analyses of additional outcome definitions, rather than as stratified or subgroup analyses. We have updated the Methods, Results and relevant table/figure labels accordingly to reflect this.

We have made the following changes in the manuscript to clarify this:

Under the Method section,

*“Results were stratified by sex, age groups, ethnic groups, types of gabapentinoid, daily dose levels and different underlying comorbidities status (S2 Appendix).” (P.12, Line 248 to 249)*

*“In secondary analyses, we used alternative outcome definitions to capture different types of drug poisoning, specifically accidental poisoning and intentional self-poisoning (S2 Appendix) [5]. Each outcome definition was analysed in a separate SCCS model using the same exposure definitions and adjustment strategy as in the primary analysis.” (P.12 to 13, Line 251 to 255)*

Under Results section,

*“Subgroups, Secondary and Sensitivity analyses*

*Most of the subgroup and secondary analyses showed a similar risk pattern (S14 to S18 Tables and S8 to S11 Fig).” (P.17, Line 357 to 359)*

Under Discussion section,

*“Fourth, in the two secondary analyses of accidental or intentional drug poisoning, the intention of drug poisoning may potentially be misclassified in clinical setting.” (P.20, Line 430 to 431)*

P26, Table 1. A very minor point, but 1 decimal place for the percentages is probably sufficient.

Response: We would like to thank the reviewer for the comment. We have now changed all percentages to 1 decimal place.

Reviewer #2: Review for PMEDICINE-D-25-03057R1

This study attempts to examine the association between gabapentinoid treatment, concurrent use with opioids or benzodiazepines, and the risk of drug poisoning. The study used the UK Clinical Practice Research Datalink database between January 1, 2010, and December 31, 2020, and identified 16,827 patients who had a diagnosis of all-cause drug poisoning. The authors used a self-controlled case series (SCCS), a within-individual study design, to test the associations.

Response: We would like to thank the reviewer for summarising the design of this study.

Major concerns

*       Introduction: The rationale for studying all-cause drug poisoning is not well justified. The study included patients with drug poisonings, "defined as mental and behavioral disorders due to psychoactive substances (ICD-10, F11-F16, F18-F19), poisoning by drugs, medicaments and biological substances (T36-T50), accidental poisoning (X40-X44), intentional self poisoning (X60-X64), assault by drugs (X85) and poisoning by drugs, medicaments and biological substances undetermined intent (Y10-Y14) (Supplementary Table 2).[30-32]"  The rationale for studying this wide range of causes for drug poisoning is not supported by potential mechanisms. Nor does it have evidence to support the study of combining all causes of drug poisoning, each of which likely differs in underlying biological and clinical mechanisms.

Response: We thank the reviewer for this thoughtful comment and agree that drug poisoning is a heterogeneous outcome, with different clinical and behavioural mechanisms underlying accidental, intentional and undetermined events.

In our study we chose a broad drug-poisoning definition for two main reasons. First, there are plausible mechanisms by which gabapentinoids could contribute to multiple types of poisoning. Gabapentin and pregabalin can cause central nervous system and respiratory depression, dizziness, and impaired judgement, and are frequently co-prescribed or co-used with opioids, benzodiazepines and other sedatives. These pharmacological effects may increase the risk of both intentional self-poisoning and accidental overdoses or mixed-intent events. Previous studies also show that gabapentinoids are involved in both intentional and unintentional poisonings [2, 6]. The actual biological mechanism is yet to be understood, but these are clinically relevant and important outcomes that we have to investigate in view of the increasing use of gabapentinoids.

Second, the coding of poisoning “intent” in healthcare records is not perfect, with poisoning with self-harm intent often classified as accidental or undetermined poisonings, and vice versa [7, 8]. To avoid differential misclassification across intent categories, several epidemiological studies, including one from the UK Office for National Statistics, have adopted composite definitions of all-cause drug poisoning [5, 9-11]. Together, they used combinations of different ICD-10 drug-poisoning codes to define a single outcome, similar to the approach adopted in our study. The primary outcome in this study was designed to be consistent with this practice after consulting the opinions from clinicians and psychiatrists.

Nonetheless, we agree that it is important to explore whether our findings are driven by specific subtypes of poisoning. Hence, we included two additional secondary analyses by changing the definition of the outcome to accidental poisoning or intentional self-poisoning and investigate whether adopting a specific intent of poisoning would affect the results in the main analysis. The results of these analyses were presented in S18 Table.

*       While the SCCS design controls for potential confounders that did not change over time, it does not adjust for confounders that change over time, such as the presence of a disease indication for treatment or disease severity. The study findings are subject to confounding by indication and confounding by disease severity because the reference is a no-treatment period, a duration during which an individual has no disease diagnosis or has the diagnosis but the disease condition is not clinically severe enough to require treatment. The use of a no-treatment period likely makes the reference group artificially superior (low risk of outcomes) to any studied treatment periods, leading to a higher risk of all-cause drug poisoning in the treatment periods vs the non-treatment period.

Response: We thank the reviewer for this important comment and agree that time-varying confounders and disease severity can be a concern in pharmacoepidemiological studies. Hence, we attempted to mitigate these issues with the study design and statistical analysis.

In the statistical analysis model, we explicitly adjusted for time-varying proxies of disease severity and clinical instability, especially concomitant prescriptions for opioids, antiseizure medications, hypnotics and anxiolytics, benzodiazepines, antidepressants, antipsychotics, lithium and NSAIDs. The use of these medications was initiated, discontinued or intensified in response to changes in pain and psychiatric conditions, and therefore, provide information on the evolution of underlying conditions (time-varying) throughout the observation period, including both treatment and non-treatment time of gabapentinoids.

In addition, we specifically modelled a 90-day pre-treatment risk window to separate the period in which worsening symptoms and emerging risk factors are likely to prompt gabapentinoid initiation from the remaining non-treatment time [13]. As shown in the results, the risk of drug poisoning was highest in this pre-treatment window and then gradually declined after gabapentinoid initiation, although it remained above the non-treatment reference level. It is also noted that such an approach was also adopted in previous studies looking into the risk of drug poisoning or other outcomes with other medications [9, 14, 15].

Directly addressing the reviewer’s concern about “artificially superior reference period”, we repeated the analysis starting observation from the first recorded neuropathic/chronic pain diagnosis thereby reducing dilution of the reference by long pre-indication time in one of our sensitivity analyses (S19 Table), and findings were consistent with the primary analysis. We further assessed key SCCS assumptions using prespecified SCCS extensions for event-dependent observation and event-dependent exposure, again with consistent results to the main analysis.

Last but not least, we would also like to draw the reviewer’s attention to our negative control analysis, in which we observed no association between the negative control outcome and gabapentinoid treatment. If the non-treatment period were truly an artificially low-risk period, we would expect to see a higher risk of the negative control outcome during treatment compared with non-treatment. However, no such association was observed. This finding supports the interpretation that our main results are unlikely to be driven by an artefact of lower baseline risk during non-treatment periods or by residual confounding.

Nevertheless, we agree that residual confounding by unmeasured time-varying factors cannot be completely excluded. As noted in the limitations section, the findings should therefore be interpreted with caution, taking into account the potential impact of these unmeasured time-varying confounders.

*“Finally, similar to other observational studies, we cannot rule out the effect of unmeasured time-varying confounders such as transient socioeconomic status, life events and use of illicit drugs,* *although the negative control analysis result does not suggest issues with residual confounders.” (P.21, Line 439 to 441)*

*       Conclusion (Page 14, lines 299-301): "…the incidence was highest in the 90-day period before treatment, suggesting that the start of treatment tends to coincide with a period of increased risk of drug poisoning." This conclusion is confusing and lacks clinical justification. Clinically, gabapentinoids are recommended (at least in the US) as an alternative pain treatment to opioids or as a co-use treatment with opioids to avert or reduce the risk of opioid use disorder or overdose. Is it possible that gabapentinoids were initiated as alternative treatment to further minimize drug poisoning after being diagnosed (that's why the risk was highest in the 90 days before the treatment initiation)?

Response: We thank the reviewer for this thoughtful comment regarding the first part of the Discussion section and agree that the interpretation of the temporal pattern around treatment initiation should be explained more clearly.

First of all, we would like to emphasise that this study was conducted using the UK healthcare database, reflecting the real-world clinical practice in the UK. In the UK, gabapentin and pregabalin are primarily indicated for neuropathic pain and as adjunctive therapy for focal seizures, and pregabalin is also licensed for generalised anxiety disorder. While NICE guidance for neuropathic pain in adults recommended gabapentinoids as pharmacological options [16], more recent NICE guidance on chronic primary pain explicitly advises against initiating gabapentinoids for chronic primary pain because of limited benefit and concerns about harms, including misuse and dependence [17]. Thus, the main guideline-based role of gabapentinoids is not as a specific intervention to prevent opioid overdose, although we cannot completely rule out that some clinicians may occasionally use them off-label as part of an opioid-sparing strategy.

The peak in drug-poisoning risk observed immediately before gabapentinoid initiation likely reflects a period of worsening underlying conditions, which was precisely why we defined a 90-day pre-treatment period, to prevent such risk changes from affecting the SCCS assumption. The suggested clinical practice from the reviewer might be one of the reasons for the observed results and the clinical practice suggested by the reviewer may indeed contribute to the observed pattern. However, the current aimed to address risks associated with gabapentinoid treatment. Based on our results, we cannot confirm the specific reasons behind the observed increased risk during this pre-treatment window. We therefore highlight the need for further research on this issue in the revised manuscript.

“*The prescribing of gabapentinoids is likely to reflect a clinical response to worsening underlying conditions or an attempt to minimise the risk of drug poisoning by switching from alternative treatments. However, our data do not allow us to determine the precise clinical reasons for the heightened risk observed in the 90 days preceding gabapentinoid initiation, and further research is warranted*.” (P.18, Line 383 to 388)

*       Conclusion (page 15, lines 304-305): "These findings indicate that gabapentinoid use is associated with a higher risk of all-cause drug poisoning, especially at the initial phase of treatment". In some clinical cases, gabapentinoids are prescribed alone or in combination with opioids when doctors suspect patients of having symptoms or behaviors of opioid misuse, before the diagnosis of opioid poisoning is formally made. In other words, doctors may selectively prescribe gabapentinoid to individuals for whom (or individual periods during which) symptoms of opioid-related or drug poisoning due to other causes have been present but not yet diagnosed. The big question is to what extent the observed elevated risks during the gabapentinoid treatment period are due to doctors' selective prescribing behaviors (i.e., channeling bias) or due to the treatment itself, which could not be teased out by the study design and data.

Response: We thank the reviewer for this insightful comment related to the first paragraph of the Discussion section.

Our study uses a self-controlled design, in which each individual serves as their own control. The “baseline” rate is specific to each patient’s own underlying risk profile, which eliminates classic between-person channelling bias. In addition, we recognise that time-varying clinical factors may still introduce within-person channelling or protopathic bias. To address this, we adjusted for use of opioids and other neuropsychiatric medications. By adjusting for these time-varying covariates, we accounted for treatment patterns where gabapentinoids are added to, or on some occasions substituted for other medications in patients with different underlying comorbidities. Thus, the situation described by the reviewer was also captured via adjusting for these time-varying covariates.

We note that the risk of poisoning remained elevated throughout the treatment period and did not return to reference level even during longer-term exposure. If gabapentinoid treatment was not associated with increased risk of drug-poisoning, and if the heightened clinical vulnerability were confined to the immediate peri-initiation period, we would expect the risk to fall back towards individuals’ baseline level during longer-term treatment periods. The sustained elevation, also supported by the spline-based analysis, suggests that gabapentinoid-treated periods mark ongoing high-risk of drug poisoning.

Finally, to explore the possibility that drug-poisoning events might themselves influence subsequent gabapentinoid prescribing (event-dependent exposure) as raised by the reviewer, we conducted an SCCS extension analysis allowing for event-dependent exposure [20]. The estimates from this analysis were consistent with those from the primary analysis, suggesting that event-driven changes in prescribing are unlikely to fully explain the observed associations.

Taken together, the design of our study and these additional analyses support the interpretation that periods of gabapentinoid treatment is associated with an elevated risk of drug poisoning, not solely attributable to channelling of gabapentinoids to patients already on a trajectory towards poisoning.

*       Study participants: It is unclear what disease condition was treated by gabapentinoid treatment among the study sample. Could an individual be prescribed gabapentin for only one condition or different conditions over the study period? The potential disease condition triggering gabapentinoid prescribing was not accounted for in the analysis, leading to potential residual confounding.

Response: We thank the reviewer for raising this important point.

This is precisely one of the reasons we adopted the SCCS design. By comparing different time periods within the same individual, the SCCS design inherently controls for all fixed patient characteristics that are often strongly related to indication, such as chronic pain susceptibility, long-standing psychiatric comorbidities, genetic risk and stable lifestyle factors [12, 13]. These factors, which would be difficult to fully capture with measured covariates, are held constant within-person and therefore cannot confound the within-individual comparisons.

We recognise that time-varying aspects of disease indication and severity may still introduce residual confounding. To mitigate this, we (i) modelled a 90-day pre-treatment period to capture the clinical deterioration and changing treatment needs that precede gabapentinoid initiation, and (ii) adjusted for a range of time-varying concomitant medications (including opioids and other neuropsychiatric medications) that serve as proxies for underlying conditions and their severity over time. Furthermore, our negative control analyses did not reveal patterns suggestive of strong residual confounding by unmeasured time-varying factors.

However, while that will not affect our results, we agree that our data do not allow us to directly identify the specific indication for each gabapentinoid prescription, or to distinguish whether an individual received gabapentinoids for a single condition or for different conditions over time. This limitation is common to many observational studies based on routine healthcare records, as prescriptions are frequently not linked to a diagnostic code at the level of individual prescriptions. To ensure this limitation is communicated well to the readers, we have added the following in the limitation section:

“*Fifth, as prescribing records in CPRD are not linked to diagnostic codes, we were unable to identify the specific clinical indications for which gabapentinoids were prescribed at each treatment episode.”* (P.20 to 21, Line 433 to 435)

*       Exposure (page 8, lines 161-165): The rationale for dividing person-time into 6 mutually risk windows is not clinically justified.

Response: We thank the reviewer for this helpful comment and agree that the rationale for the risk-period definition should be explained.

In our analysis, we divided person-time into discrete windows to reflect the real-world clinical utilisation of gabapentinoids within the UK healthcare system, while also capturing the clinically most relevant phases surrounding treatment initiation.

1. Pre-treatment window (90 days before first prescription)

We specified a 90-day pre-treatment period to reflect the clinical build-up of symptoms and treatment escalation that typically precede initiation of gabapentinoids for neuropathic or chronic pain and related conditions. This choice was also supported by our spline-based exploratory analysis, which showed a gradual rise in the incidence of drug poisoning in the months immediately before the first gabapentinoid prescription, rather than an abrupt change only on the day of initiation.

2. Early exposure windows in 28-day blocks (0–28, 29–56, 57–84 days)

Within the first 84 days after initiation, we used 28-day categories for two reasons. First, in our cohort the median prescription duration for gabapentinoids was 28 days, so 28-day intervals align closely with the dominant prescribing cycle and the clinical practice of reassessing analgesic response and tolerability on a monthly basis. Second, both clinical input and our spline analyses suggested that any treatment-related change in risk were manifested during the early titration and stabilisation phase (first 2 to 3 months). Using equal-length 28-day blocks within this window allowed us to distinguish very early risk (0 to 28 days) from subsequent early exposure (29 to 56 and 57 to 84 days) in a transparent way.

3. Remaining treatment time

Beyond 84 days, the spline curves suggested a plateaued profile. We therefore grouped the remaining exposed time into a single category to avoid unstable estimates while still contrasting early versus later treatment.

In addition, the untreated period (reference period) consists of all person-time outside the pre-treatment and on-treatment windows, while individuals remain under observation and eligible for treatment. This untreated period represents the background risk of drug poisoning when the patient was not treated with gabapentinoids. Using this untreated time as the reference is consistent with standard SCCS practice and allows us to compare periods around treatment initiation with a patient’s own baseline risk [14, 15, 21], thereby helping to distinguish clustering of events around treatment changes from their longer-term underlying risk.

*       Exposure (page 8, line 155): "Gabapentinoid prescriptions that were less than or equal to 90 days apart were treated as a continuous treatment period." There is no justification for the use of 90 days as the cut-off point for the grace period. A 90-day gap for some conditions (neuropathic pain) could be clinically considered as treatment discontinuation.

Response: We thank the reviewer for highlighting this important point regarding our choice of a 90-day interval when concatenating prescriptions into continuous treatment periods.

Our primary rationale for allowing prescriptions ≤90 days apart to be treated as part of the same treatment episode was to reduce exposure misclassification arising from real-world adherence patterns. In UK primary care, repeat gabapentinoid prescriptions may be issued for up to 2 to 3 months of supply, and patients can also have irregular refill behaviour, for instance, delayed collection, stockpiling, or small administrative gaps. Treating any gap beyond the nominal prescription duration as immediate and definitive discontinuation risks artificially fragmenting a clinically continuous course of therapy into multiple short episodes and assigning substantial treated time as non-treated.

Several studies have also used gaps as permissible intervals when defining persistence or continuous treatment episodes [14, 22]. Importantly, prescriptions separated by intervals of ≥91 days were classified as non-treatment time in our main analysis. Thus, our approach only linked prescriptions that were relatively close together in time, where clinical continuity of treatment is most plausible.

To address the reviewer’s concern, we conducted an additional sensitivity analysis in which we did not combine any gabapentinoid prescriptions. In this analysis, each prescription was treated as a distinct exposure interval without concatenation, and any time beyond the recorded prescription duration was classified as non-treated, even if the next prescription started shortly afterwards. The results of this no combination sensitivity analysis resemble to those of the main analysis and did not alter our conclusions.

In the Method section, we now added one additional sensitivity analysis

*“h) not combining gabapentinoid prescriptions if they were less than or equal to 90 days apart (an unplanned sensitivity analysis)” (P.13, Line 270 to 271)*

We also added the results into S19 Table.

| **Risk window** | **Number of events** | **Patient-years** | **Crude incidence (per 100 patient-years)** | **aIRR (95% CI)** | **P value** |
| --- | --- | --- | --- | --- | --- |
| **Not combining GABA prescriptions that were less than or equal to 90 days apart (n=16,791)** |  |  |  |  |  |
| 90 days before treatment | 2,189 | 10,511.39 | 20.83 (19.95 - 21.70) | 1.83 (1.73 - 1.92) | <0.001 |
| First 28 days of treatment period | 1,659 | 10,550.84 | 15.72 (14.97 - 16.48) | 1.32 (1.24 - 1.40) | <0.001 |
| 29-56 days of treatment period | 700 | 4,728.04 | 14.81 (13.71 - 15.90) | 1.26 (1.16 - 1.37) | <0.001 |
| 57-84 days of treatment period | 370 | 2,671.98 | 13.85 (12.44 - 15.26) | 1.18 (1.06 - 1.32) | 0.003 |
| Remaining time of treatment period | 1,093 | 8,045.21 | 13.59 (12.78 - 14.39) | 1.20 (1.11 - 1.30) | <0.001 |
| Reference period | 10,780 | 100,931.10 | 10.68 (10.48 - 10.88) | 1.00 (1.00 - 1.00) | NA |

Other concerns

*       Of the identified 16,827 patients with all-cause drug poisonings, what is the distribution by types of drug poisoning?
Response: We would like to thank the reviewer for raising this question.

Most of the patients have multiple poisoning diagnosis on the same day. This aligns to the response from the first concern as patients are often coded with multiple drug poisoning code due to uncertainty of the intent and complexity of the drug poisoning event. Hence, we used all-cause drug poisoning to define the outcome of event to ensure that we capture all poisoning related event.

Nevertheless, we have counted the types of poisoning related to the incident event captured in the analysis. Please kindly note that some patients may have more than one poisoning related diagnosis on the same day.

| **Poisoning type** | **Count** | **Percentage** |
| --- | --- | --- |
| Accidental poisoning  (ICD-10, X40-X44) | 3513 | 20.9 |
| Intentional self poisoning  (ICD-10, X60-X64) | 7940 | 47.2 |
| Mental and behavioural disorders due to psychoactive substances  (ICD-10, F11-F16, F18-F19) | 5220 | 31.0 |
| Poisoning by drugs, medicaments and biological substances  (ICD-10, T36-T50) | 11888 | 70.6 |
| Poisoning by drugs, medicaments and biological substances – undetermined intent  (ICD-10, Y10-Y14) | 87 | 0.5 |

Reviewer #3: Thank you for the opportunity to review this important paper addressing a priority challenge: increasing rates of use of gabapentin. This paper is well written and has robust sensitivity analyses etc.

Response: We would like to thank the reviewer for the encouraging comments on our study.

I had a few comments that I hope you can address: 1) While the authors mention the potential impact of time varying confounders, they do not address clinical time varying confounders that may precipitate a prescription for gaba and potentially put a patient at higher risk for poisoning. Minimally I hope the authors can talk more about this potential in their limitations or ideally provide some more of a breakdown of clinical characteristics in the pre vs post-GABA Rx period (not pre or post event period).

Response: We would like to thank the reviewer for the insightful comment. We understand the potential impact of time varying clinical factors in the study, hence, in the analysis model, we have also adjusted for concomitant medications that will be potential confounders affecting both the exposure and outcome of interest in this study question. They also act as a proxy for the changing of clinical condition of the patient. In addition, the findings from our negative control analysis did not indicate that unmeasured time-varying confounding was likely to have affected our results. Nevertheless, in the final sentence of the limitation section, we mentioned that we cannot rule out the effect of unmeasured time-varying confounders.

To further explore the reviewer’s concern, we conducted an additional descriptive analysis comparing clinical characteristics in the periods before and after gabapentinoid initiation among included individuals. Although our self-controlled case series design does not define a conventional baseline index date, we examined use of concomitant medications within the 6 months before and the 6 months after the first gabapentinoid prescription. We found that the use of concomitant medications increased in the 6 months following treatment initiation, as shown below, which is consistent with clinical deterioration and greater complexity of care around the time gabapentinoids are prescribed.

| **Variables** | **Six months before first GABA prescription (n, %)** | **Six months after first GABA prescription (n, %)** |
| --- | --- | --- |
| **Use of other medications** |  |  |
| Antiseizure medications | 756 (4.5) | 990 (5.9) |
| Opioids | 9690 (57.6) | 10583 (62.9) |
| Hypnotics and anxiolytics, except benzodiazepines | 2538 (15.1) | 2918 (17.3) |
| Benzodiazepines | 3641 (21.6) | 3884 (23.1) |
| Antidepressants | 10736 (63.8) | 11756 (69.9) |
| Antipsychotics | 2478 (14.7) | 2865 (17.0) |
| Lithium | 156 (0.9) | 181 (1.1) |
| NSAIDs | 5618 (33.4) | 5738 (34.1) |
| Any of the above | 14513 (86.2) | 15612 (92.8) |
| **Comorbidities diagnosis** |  |  |
| Neuropathic pain or chronic pain | 5658 (33.6) | 6644 (39.5) |
| Substance misuse | 3449 (20.5) | 3398 (20.2) |
| Bipolar and mania | 324 (1.9) | 334 (2.0) |
| Depression | 3548 (21.1) | 3661 (21.8) |
| Anxiety disorders | 2855 (17.0) | 3163 (18.8) |
| Schizophrenia | 157 (0.9) | 168 (1.0) |
| Other psychosis | 182 (1.1) | 197 (1.2) |
| Insomnia | 375 (2.2) | 400 (2.4) |
| Any of the above mental health conditions | 5571 (33.1) | 5936 (35.3) |

2) If the authors can comment specifically about any potential issues with generalizability of study findings beyond the context of the NHS system that would also be instructive for readers. Overall, a well written paper.

Response: We would like to thank the reviewer for highlighting the potential issues with generalisability of the study findings. Our study used routinely collected data from English NHS primary and secondary care, and we agree that patterns of gabapentinoid prescribing, concomitant opioid/benzodiazepine use, healthcare access and coding practices may differ in other healthcare systems. We have therefore added this to the limitation section to clarify that the findings in this study should be generalised cautiously beyond the NHS setting.

*“Sixth, our findings are based on routinely collected data from NHS primary and secondary care in England, and patterns of gabapentinoid prescribing, concomitant opioid or benzodiazepine use and diagnosis practice may differ in other healthcare systems. The observed risks may not be directly generalisable to countries with different model of care.” (P.21, Line 435 to 438)*

General editorial requests:

*       We ask every co-author listed on the manuscript to fill in a contributing author statement, making sure to declare all competing interests. If any of the co-authors have not filled in the statement, we will remind them to do so when the paper is revised. If all statements are not completed in a timely fashion this could hold up the re-review process. If new competing interests are declared later in the revision process, this may also hold up the submission. Should there be a problem getting one of your co-authors to fill in a statement we will be in contact. Please do not add or remove authors without first discussing this with the handling editor. You can see our competing interests policy here: [https://eur01.safelinks.protection.outlook.com/?url=http%3A%2F%2Fjournals.plos.org%2Fplosmedicine%2Fs%2Fcompeting-interests&data=05%7C02%7Ckenneth.man%40ucl.ac.uk%7C66941bbbc0294d332fe708de28eee504%7C1faf88fea9984c5b93c9210a11d9a5c2%7C0%7C0%7C638993203683328988%7CUnknown%7CTWFpbGZsb3d8eyJFbXB0eU1hcGkiOnRydWUsIlYiOiIwLjAuMDAwMCIsIlAiOiJXaW4zMiIsIkFOIjoiTWFpbCIsIldUIjoyfQ%3D%3D%7C0%7C%7C%7C&sdata=nX4IKevac%2BHiZ5aPm%2Bf1uptFdQEK9JY1o8sA3ZmPZDk%3D&reserved=0](https://eur01.safelinks.protection.outlook.com/?url=http%3A%2F%2Fjournals.plos.org%2Fplosmedicine%2Fs%2Fcompeting-interests&data=05%7C02%7Csiu.yuen.13%40ucl.ac.uk%7C760c7d08b48f485334d308de28fc9e6d%7C1faf88fea9984c5b93c9210a11d9a5c2%7C0%7C0%7C638993262602449353%7CUnknown%7CTWFpbGZsb3d8eyJFbXB0eU1hcGkiOnRydWUsIlYiOiIwLjAuMDAwMCIsIlAiOiJXaW4zMiIsIkFOIjoiTWFpbCIsIldUIjoyfQ%3D%3D%7C0%7C%7C%7C&sdata=OkJkYM98LJaz5JzLxXeN1pFG%2FLHcywFYwkrOS9I22f4%3D&reserved=0).

Response: We would like to thank the editors for the reminder. The competing interests have been verified with the co-authors and included on P.22 and included with the submission. We have also included a contributing author statement on P.23 to 24.

**Competing interests:**

“ASCY reports grant from the University College London Hospitals NIHR Biomedical Research Centre, College of Mental Health Pharmacy, and UK Turing Scheme. AYLC reports grant from the AIR@innoHK programme of the Hong Kong Innovation and Technology Commission. JFH reports grants from UK Research and Innovation grant and the University College London Hospitals NIHR Biomedical Research Centre and the NIHR North Thames Applied Research Collaboration. He received consulting fees from the Wellcome Trust, Juli Health and and Swiss Re. DPJO reports grants from the University College London Hospitals NIHR Biomedical Research Centre and the NIHR North Thames Applied Research Collaboration. WCYL reports grants from Diabetes UK, AIR@InnoHK of the Hong Kong Innovation and Technology Commission. ICKW reports grant from WHO, IQVIA, Amgen, Janssen, GSK, Novartis, Pfizer, Bayer and Bristol-Myers Squibb and Takeda, Institute for Health Research in England, European Commission, National Health and Medical Research Council in Australia, The European Union’s Seventh Framework Programme for research, technological development, Research Grants Council Hong Kong and Health and Medical Research Fund Hong Kong. He received payment for expert testimony for Appeal Court in Hong Kong. He serves on advisory committees for Member of Hong Kong Pharmacy and Poisons Board, as a member of the Expert Committee on Clinical Events Assessment Following COVID-19 Immunization in Hong Kong, as a member of the Advisory Panel on COVID-19 Vaccines of the Hong Kong Government, as the non-executive director of Jacobson Pharma Corp. Ltd. in Hong Kong, as the founder and director of Therakind Limited (UK), Advance Data Analytics for Medical Science (ADAMS) Limited (HK) and OCUS Innovation Limited (HK, Ireland and UK). LW reports grants from the UK Cure Parkinson’s Trust and UK NIHR. KKCM reports grants from CW Maplethorpe Fellowship, the European Union Horizon 2020, the UK National Institute of Health Research, the South Korea Ministry of Food and Drug Safety, the Hong Kong Research Grant Council and Hong Kong Innovation and Technology Commission, consultancy from IQVIA, AstraZeneca, outside of the submitted work. BC and FMCB declare that no competing interests exist.”

**Contributing author statement:**

“Andrew S.C. Yuen

Roles: Conceptualization, Data curation, Formal analysis, Investigation, Methodology, Funding acquisition, Resources, Validation, Visualization, Writing – original draft, Writing – review & editing

Boqing Chen

Roles: Formal analysis, Investigation, Validation, Writing – review & editing

Adrienne Y.L. Chan

Roles: Methodology, Investigation, Writing – review & editing

Joseph F. Hayes

Roles: Conceptualization, Investigation, Writing – review & editing

David P.J. Osborn
Roles: Investigation, Writing – review & editing

Frank M.C. Besag

Roles: Investigation, Writing – review & editing

Wallis C.Y. Lau

Roles: Investigation, Writing – review & editing

Ian C.K. Wong

Roles: Investigation, Writing – review & editing

Li Wei

Roles: Supervision, Investigation, Writing – review & editing

Kenneth K.C. Man

Roles: Conceptualization, Data curation, Supervision, Investigation, Methodology, Funding acquisition, Resources, Project administration, Validation, Writing – review & editing”

*       Please upload any figures associated with your paper as individual TIF or EPS files with 300dpi resolution at resubmission; please read our figure guidelines for more information on our requirements: [https://eur01.safelinks.protection.outlook.com/?url=http%3A%2F%2Fjournals.plos.org%2Fplosmedicine%2Fs%2Ffigures&data=05%7C02%7Ckenneth.man%40ucl.ac.uk%7C66941bbbc0294d332fe708de28eee504%7C1faf88fea9984c5b93c9210a11d9a5c2%7C0%7C0%7C638993203683348647%7CUnknown%7CTWFpbGZsb3d8eyJFbXB0eU1hcGkiOnRydWUsIlYiOiIwLjAuMDAwMCIsIlAiOiJXaW4zMiIsIkFOIjoiTWFpbCIsIldUIjoyfQ%3D%3D%7C0%7C%7C%7C&sdata=kvfZvcLaXubSZ8B9xsUP8D4wSWugD%2FwQ9QyO%2BfIfZro%3D&reserved=0](https://eur01.safelinks.protection.outlook.com/?url=http%3A%2F%2Fjournals.plos.org%2Fplosmedicine%2Fs%2Ffigures&data=05%7C02%7Csiu.yuen.13%40ucl.ac.uk%7C760c7d08b48f485334d308de28fc9e6d%7C1faf88fea9984c5b93c9210a11d9a5c2%7C0%7C0%7C638993262602461838%7CUnknown%7CTWFpbGZsb3d8eyJFbXB0eU1hcGkiOnRydWUsIlYiOiIwLjAuMDAwMCIsIlAiOiJXaW4zMiIsIkFOIjoiTWFpbCIsIldUIjoyfQ%3D%3D%7C0%7C%7C%7C&sdata=Jq6L6LjWaxt0FF%2FYfwnWELtkWJA91VSz7Uh408RCguE%3D&reserved=0). While revising your submission, we strongly recommend that you use PLOS's NAAS tool ([https://eur01.safelinks.protection.outlook.com/?url=https%3A%2F%2Fngplosjournals.pagemajik.ai%2Fartanalysis&data=05%7C02%7Ckenneth.man%40ucl.ac.uk%7C66941bbbc0294d332fe708de28eee504%7C1faf88fea9984c5b93c9210a11d9a5c2%7C0%7C0%7C638993203683364907%7CUnknown%7CTWFpbGZsb3d8eyJFbXB0eU1hcGkiOnRydWUsIlYiOiIwLjAuMDAwMCIsIlAiOiJXaW4zMiIsIkFOIjoiTWFpbCIsIldUIjoyfQ%3D%3D%7C0%7C%7C%7C&sdata=68VQFkrqzzt172MnZCr1xM824MhsIqvV%2FXkHCMdivaw%3D&reserved=0](https://eur01.safelinks.protection.outlook.com/?url=https%3A%2F%2Fngplosjournals.pagemajik.ai%2Fartanalysis&data=05%7C02%7Csiu.yuen.13%40ucl.ac.uk%7C760c7d08b48f485334d308de28fc9e6d%7C1faf88fea9984c5b93c9210a11d9a5c2%7C0%7C0%7C638993262602474995%7CUnknown%7CTWFpbGZsb3d8eyJFbXB0eU1hcGkiOnRydWUsIlYiOiIwLjAuMDAwMCIsIlAiOiJXaW4zMiIsIkFOIjoiTWFpbCIsIldUIjoyfQ%3D%3D%7C0%7C%7C%7C&sdata=jmK7igH6XrpNb7lmthxKh9NNo0yI7gbRvR6JAOVwnzA%3D&reserved=0)) to test your figure files. NAAS can convert your figure files to the TIFF file type and meet basic requirements (such as print size, resolution), or provide you with a report on issues that do not meet our requirements and that NAAS cannot fix.

After uploading your figures to PLOS's NAAS tool - [https://eur01.safelinks.protection.outlook.com/?url=https%3A%2F%2Fngplosjournals.pagemajik.ai%2Fartanalysis&data=05%7C02%7Ckenneth.man%40ucl.ac.uk%7C66941bbbc0294d332fe708de28eee504%7C1faf88fea9984c5b93c9210a11d9a5c2%7C0%7C0%7C638993203683380499%7CUnknown%7CTWFpbGZsb3d8eyJFbXB0eU1hcGkiOnRydWUsIlYiOiIwLjAuMDAwMCIsIlAiOiJXaW4zMiIsIkFOIjoiTWFpbCIsIldUIjoyfQ%3D%3D%7C0%7C%7C%7C&sdata=cjkBygsEGduiRp1g2kOafErKcuEUEOVsX2GljnlX7uY%3D&reserved=0](https://eur01.safelinks.protection.outlook.com/?url=https%3A%2F%2Fngplosjournals.pagemajik.ai%2Fartanalysis&data=05%7C02%7Csiu.yuen.13%40ucl.ac.uk%7C760c7d08b48f485334d308de28fc9e6d%7C1faf88fea9984c5b93c9210a11d9a5c2%7C0%7C0%7C638993262602487735%7CUnknown%7CTWFpbGZsb3d8eyJFbXB0eU1hcGkiOnRydWUsIlYiOiIwLjAuMDAwMCIsIlAiOiJXaW4zMiIsIkFOIjoiTWFpbCIsIldUIjoyfQ%3D%3D%7C0%7C%7C%7C&sdata=I9vGtOlMpVKxB0EQJphmvX1wn2tiJfXp5%2F0e6wqyAZ8%3D&reserved=0), NAAS will process the files provided and display the results in the "Uploaded Files" section of the page as the processing is complete.
If the uploaded figures meet our requirements (or NAAS is able to fix the files to meet our requirements), the figure will be marked as "fixed" above. If NAAS is unable to fix the files, a red "failed" label will appear above.
When NAAS has confirmed that the figure files meet our requirements, please download the file via the download option, and include these NAAS processed figure files when submitting your revised manuscript.

Response: Thank you for the reminder regarding the figure format. We have now converted all three main figures to files that meet the required specifications and uploaded each as an individual file in the NAAS system. We have also moved the figure captions in the manuscript to immediately follow the paragraphs in which the figures are first cited.

* We note that you state in the manuscript that you obtained ethical approval. Please amend your ethics statement in the metadata in Editorial Manager, to include the same statement there.

Response: Thank you for the reminder. We have now added the same ethics statement to the metadata in Editorial Manager to ensure consistency with the manuscript.


FORMATTING - GENERAL
*       Abstract: Please structure your abstract using the PLOS Medicine headings (Background, Methods and Findings, Conclusions). Please combine the Methods and Findings sections into one section.

Response: We thank the editor for the reminder. We have now changed the headings of the abstract to align with the structure of PLOS Medicine.

*       At this stage, we ask that you include a short, non-technical Author Summary of your research to make findings accessible to a wide audience that includes both scientists and non-scientists. The Author Summary should immediately follow the Abstract in your revised manuscript. This text is subject to editorial change and should be distinct from the scientific abstract. Ideally each sub-heading should contain 2-3 single sentence, concise bullet points containing the most salient points from your study. In the final bullet point of 'What Do These Findings Mean?', please include the main limitations of the study in non-technical language. Please see our author guidelines for more information: [https://eur01.safelinks.protection.outlook.com/?url=https%3A%2F%2Fjournals.plos.org%2Fplosmedicine%2Fs%2Frevising-your-manuscript%23loc-author-summary&data=05%7C02%7Ckenneth.man%40ucl.ac.uk%7C66941bbbc0294d332fe708de28eee504%7C1faf88fea9984c5b93c9210a11d9a5c2%7C0%7C0%7C638993203683401620%7CUnknown%7CTWFpbGZsb3d8eyJFbXB0eU1hcGkiOnRydWUsIlYiOiIwLjAuMDAwMCIsIlAiOiJXaW4zMiIsIkFOIjoiTWFpbCIsIldUIjoyfQ%3D%3D%7C0%7C%7C%7C&sdata=VBex6zEGEX%2FnWJFP2qSVDfxue5EiXGdsQ7lP6q2snpU%3D&reserved=0](https://eur01.safelinks.protection.outlook.com/?url=https%3A%2F%2Fjournals.plos.org%2Fplosmedicine%2Fs%2Frevising-your-manuscript%23loc-author-summary&data=05%7C02%7Csiu.yuen.13%40ucl.ac.uk%7C760c7d08b48f485334d308de28fc9e6d%7C1faf88fea9984c5b93c9210a11d9a5c2%7C0%7C0%7C638993262602500354%7CUnknown%7CTWFpbGZsb3d8eyJFbXB0eU1hcGkiOnRydWUsIlYiOiIwLjAuMDAwMCIsIlAiOiJXaW4zMiIsIkFOIjoiTWFpbCIsIldUIjoyfQ%3D%3D%7C0%7C%7C%7C&sdata=qZSAEGAaNdqDJ53p3nRXVsGAofzfbd1HtvvOK2CqNyo%3D&reserved=0).

Response: We thank the editor for the reminder. We have now placed the Author Summary immediately after the Abstract.

**Author Summary**

“**Why was this study done?**

Use of gabapentinoids has increased substantially over the past decade, and there are growing concerns that they may contribute to drug poisoning and overdose, particularly when combined with opioids or benzodiazepines.

Previous studies have mainly focused on high-risk populations or opioid users, and only one has examined unintentional overdose, with important limitations such as inadequate adjustment for concomitant medications.

There is a need to characterise how the risk of drug poisoning changes before and after starting gabapentinoids, and during concomitant opioid/ benzodiazepine use, to provide clinicians with robust evidence to guide safer prescribing and monitoring.

**What did the researchers do and find?**

The authors used UK electronic health records from CPRD Aurum, linked to HES and ONS data from England, to conduct an SCCS study of 16,827 adults who had a first recorded drug poisoning event and were prescribed gabapentinoids.

The risk of drug poisoning was more than doubled in the 90 days before starting gabapentinoids (aIRR = 2.09, 95% CI [1.97, 2.23]; p < 0.001), remained elevated in the first 28 days of treatment (aIRR = 1.81, 95% CI [1.66, 1.99]; p < 0.001) and was still raised during the remaining treatment period (aIRR = 1.11, 95% CI [1.05, 1.17]; p < 0.001) compared with non-treatment periods.

Concurrent opioid/ benzodiazepine use further increased the risk, and the additional case-case time-control analysis showed higher odds of gabapentinoid exposure before drug poisoning (aOR = 1.36, 95% CI [1.12, 1.65]; p = 0.002), supporting the main findings.

**What do these findings mean?**

The findings suggest that gabapentinoids are often started during periods of already heightened vulnerability to drug poisoning, and that risk remains modestly elevated during treatment, and further elevated when opioids or benzodiazepines are co-prescribed.

Clinicians should be aware of this increased vulnerability, monitor patients closely around treatment initiation, and avoid or minimise concurrent opioid or benzodiazepine use where possible to support safer prescribing.

The SCCS approach cannot fully rule out the influence of unmeasured time-varying confounders, such as transient changes in socioeconomic status, major life events, or illicit drug use, although the negative control analysis did not indicate substantial residual confounding.” (P.5 to 6, Line 75 to 115)

*       Please express the main results with 95% CIs as well as p values. When reporting p values please report as p<0.001 and where higher as the exact p value p=0.002, for example. Throughout, suggest reporting statistical information as follows to improve clarity for the reader "22% (95% CI [13%,28%]; p</=)". Please be sure to define all numerical values at first use.

Response: We thank the editor for the reminder. We have now included all main results with 95% confidence intervals and corresponding p values.

*       Please cite the reference numbers in square brackets. Citations should precede punctuation.

Response: We thank the reviewer for the advice. We have now ensured that all citations appear before the punctuation.


SUPPLEMENTARY MATERIAL
*       Please note that supplementary material will be posted as supplied by the authors. Therefore, please amend it according to the relevant comments outlined here.

*       Please cite your Supporting Information as outlined here: [https://eur01.safelinks.protection.outlook.com/?url=https%3A%2F%2Fjournals.plos.org%2Fplosmedicine%2Fs%2Fsupporting-information&data=05%7C02%7Ckenneth.man%40ucl.ac.uk%7C66941bbbc0294d332fe708de28eee504%7C1faf88fea9984c5b93c9210a11d9a5c2%7C0%7C0%7C638993203683428099%7CUnknown%7CTWFpbGZsb3d8eyJFbXB0eU1hcGkiOnRydWUsIlYiOiIwLjAuMDAwMCIsIlAiOiJXaW4zMiIsIkFOIjoiTWFpbCIsIldUIjoyfQ%3D%3D%7C0%7C%7C%7C&sdata=ZAtg%2Fb1IuMNS7EtMCYITrJgASCtJItpq6RqseDch2Vk%3D&reserved=0](https://eur01.safelinks.protection.outlook.com/?url=https%3A%2F%2Fjournals.plos.org%2Fplosmedicine%2Fs%2Fsupporting-information&data=05%7C02%7Csiu.yuen.13%40ucl.ac.uk%7C760c7d08b48f485334d308de28fc9e6d%7C1faf88fea9984c5b93c9210a11d9a5c2%7C0%7C0%7C638993262602514020%7CUnknown%7CTWFpbGZsb3d8eyJFbXB0eU1hcGkiOnRydWUsIlYiOiIwLjAuMDAwMCIsIlAiOiJXaW4zMiIsIkFOIjoiTWFpbCIsIldUIjoyfQ%3D%3D%7C0%7C%7C%7C&sdata=Sw3YVdmof491Z4h%2F90MjncDE7O%2Fpn1COXXLrbfJGKrg%3D&reserved=0)

Response: We thank the editor for the reminders. We have now reformatted the supplementary material and cited it in the manuscript in accordance with the guidelines.

REFERENCES
*       PLOS uses the numbered citation (citation-sequence) method and first six authors, et al.

Response: We thank the editor for the comment. We confirm that the referencing style used in the manuscript adheres to the PLOS style.

*       Please ensure that journal name abbreviations match those found in the National Center for Biotechnology Information (NCBI) databases ([https://eur01.safelinks.protection.outlook.com/?url=http%3A%2F%2Fwww.ncbi.nlm.nih.gov%2Fnlmcatalog%2Fjournals&data=05%7C02%7Ckenneth.man%40ucl.ac.uk%7C66941bbbc0294d332fe708de28eee504%7C1faf88fea9984c5b93c9210a11d9a5c2%7C0%7C0%7C638993203683452043%7CUnknown%7CTWFpbGZsb3d8eyJFbXB0eU1hcGkiOnRydWUsIlYiOiIwLjAuMDAwMCIsIlAiOiJXaW4zMiIsIkFOIjoiTWFpbCIsIldUIjoyfQ%3D%3D%7C0%7C%7C%7C&sdata=hZV5jlm83T0tb47Ya6GZaVtfj9LLag6FBz8r3iFpHhw%3D&reserved=0](https://eur01.safelinks.protection.outlook.com/?url=http%3A%2F%2Fwww.ncbi.nlm.nih.gov%2Fnlmcatalog%2Fjournals&data=05%7C02%7Csiu.yuen.13%40ucl.ac.uk%7C760c7d08b48f485334d308de28fc9e6d%7C1faf88fea9984c5b93c9210a11d9a5c2%7C0%7C0%7C638993262602532340%7CUnknown%7CTWFpbGZsb3d8eyJFbXB0eU1hcGkiOnRydWUsIlYiOiIwLjAuMDAwMCIsIlAiOiJXaW4zMiIsIkFOIjoiTWFpbCIsIldUIjoyfQ%3D%3D%7C0%7C%7C%7C&sdata=Ik7NvkIrnkDm9yVL2UUZR33lZqNxndlAJuTBmoMmkB0%3D&reserved=0)), and are appropriately formatted and capitalised.

Response: We thank the editor for the reminder. We confirm that the journal name abbreviations used in the manuscript match those listed in the National Center for Biotechnology Information (NCBI) databases.

*       Where website addresses are cited, please include the complete URL and specify the date of access (e.g. [accessed: 12/06/2023]).

Response: We would like to thank the editor for the reminder. We have now added access dates for all cited website addresses.

*       Please also see [https://eur01.safelinks.protection.outlook.com/?url=https%3A%2F%2Fjournals.plos.org%2Fplosmedicine%2Fs%2Fsubmission-guidelines%23loc-references&data=05%7C02%7Ckenneth.man%40ucl.ac.uk%7C66941bbbc0294d332fe708de28eee504%7C1faf88fea9984c5b93c9210a11d9a5c2%7C0%7C0%7C638993203683477228%7CUnknown%7CTWFpbGZsb3d8eyJFbXB0eU1hcGkiOnRydWUsIlYiOiIwLjAuMDAwMCIsIlAiOiJXaW4zMiIsIkFOIjoiTWFpbCIsIldUIjoyfQ%3D%3D%7C0%7C%7C%7C&sdata=DTYjYihJBBYFlpLz8PJS8tg0appvFP07PHYTOpoZNpA%3D&reserved=0](https://eur01.safelinks.protection.outlook.com/?url=https%3A%2F%2Fjournals.plos.org%2Fplosmedicine%2Fs%2Fsubmission-guidelines%23loc-references&data=05%7C02%7Csiu.yuen.13%40ucl.ac.uk%7C760c7d08b48f485334d308de28fc9e6d%7C1faf88fea9984c5b93c9210a11d9a5c2%7C0%7C0%7C638993262602550116%7CUnknown%7CTWFpbGZsb3d8eyJFbXB0eU1hcGkiOnRydWUsIlYiOiIwLjAuMDAwMCIsIlAiOiJXaW4zMiIsIkFOIjoiTWFpbCIsIldUIjoyfQ%3D%3D%7C0%7C%7C%7C&sdata=uznvpiaNG903oeuKt27E3m56cbyz%2FxNefae%2BqklsUNQ%3D&reserved=0) for further details on reference formatting.

Response: We can confirm that the references are formatted according to the PLOS style.


OBSERVATIONAL STUDIES

*       Abstract: Please include the study design, population and setting, number of participants, years during which the study took place (enrollment and follow up), length of follow up, and main outcome measures.

Response: We thank the editor for the reminder regarding the elements that should be included in the abstract. We have now revised the abstract as follows:

“**Background**

Consumption of gabapentinoids has increased worldwide in recent years, and the association between its use and drug poisoning is of public health concern. This study aimed to investigate the association between gabapentinoid treatment and the risk of drug poisoning.

**Methods and Findings**

In this within-individual study, we utilised data from the UK Clinical Practice Research Datalink (CPRD) Aurum database linked to the Hospital Episode Statistics (HES) and Office for National Statistics (ONS). The analysis included individuals aged 18 or above who were prescribed gabapentinoids, and had an incident all-cause drug poisoning event between 1^st^ January 2010 and 31^st^ December 2020. Using the self-controlled case series (SCCS) design, we assessed the risk of drug poisoning incidence in predefined risk periods: 90-day before treatment initiation, first 28, 29–56, 57–84 days, and the remaining treatment time. Concomitant use with opioids/ benzodiazepines was also evaluated. Adjusted incidence rate ratios (aIRR) were calculated using conditional Poisson regression. A case-case time-control (CCTC) analysis was also conducted, with adjusted odds ratio (aOR) calculated to validate the findings from the main SCCS analysis.

16,827 individuals met the inclusion criteria and were included in the SCCS analysis. The risk of drug poisoning, compared with the reference periods, increased during the first 28 days of gabapentinoid treatment (aIRR = 1.81, 95% confidence interval [CI] [1.66, 1.99]; p < 0.001), eventually dropped to 1.11 (95% CI [1.05, 1.17]; p < 0.001) in the remainder of the treatment period. Notably, the risk was doubled during the 90-day preceding treatment initiation (aIRR = 2.09, 95% CI [1.98, 2.21]; p < 0.001). Co-administration with opioids elevated the risk by 30%, while benzodiazepines increased it twofold. The CCTC analysis also detected an increased aOR of 1.36 (95% CI [1.12, 1.65]; p = 0.002) of receiving gabapentinoid treatment within 30 days prior to a drug poisoning event.

**Conclusions**

The results suggest that gabapentinod is associated with an increased risk of drug poisoning. Close monitoring throughout gabapentinoid treatment journey for drug poisoning is needed, especially at the initial phase. Concomitant use with opioid or benzodiazepines should be avoided.” (P.3 to 4, Line 42 to 74)

*       Thank you for reporting your study according to the STROBE guidelines, and including the checklist. Please also add the following statement, or similar, to the Methods: "This study is reported as per the Strengthening the Reporting of Observational Studies in Epidemiology (STROBE) guideline (S1 Checklist)." When completing the checklist, please use section and paragraph numbers, rather than page numbers.

Response: We thank the editor for the comment. We have revised the sentence in the Methods section and updated the STROBE checklist (as S1 Checklist) to include the relevant section and paragraph numbers.

*“This study is reported as per the Strengthening the Reporting of Observational Studies in Epidemiology (STROBE) guideline (S1 Checklist) [23].”* (P. 14, Line 287 to 289)


*       For all observational studies, in the manuscript text, please indicate: (1) the specific hypotheses you intended to test, (2) the analytical methods by which you planned to test them, (3) the analyses you actually performed, and (4) when reported analyses differ from those that were planned, transparent explanations for differences that affect the reliability of the study's results. If a reported analysis was performed based on an interesting but unanticipated pattern in the data, please be clear that the analysis was data driven.

Response: We thank the editor for the reminder regarding the elements that should be included in observational studies. We have revised the final paragraph of the Introduction to clearly state the hypotheses we intended to test, the analytical methods we planned to use, and the analyses we ultimately performed. The reported analyses are consistent with those originally planned.

*“In this study, we hypothesised that initiation of gabapentinoid treatment is associated with an increased incidence of all-cause drug poisoning, and that this risk differs across predefined treatment windows. We also hypothesised that concurrent opioid or benzodiazepine use would modify this association, increasing the risk during overlapping treatment periods. To test these hypotheses, we plannd and performed self-controlled case series (SCCS) analysis, comparing incidence rates of drug poisoning within individuals across non-treatment, pre-treatment, and post-initiation risk windows. This study design accounts for the diverse range of indications for gabapentinoids, which addresses the different underlying risks of drug poisoning associated with these indications, and accounting for all time-invariant confounders.” (P.8, Line 148 to 156)*

*       Please state in the Methods section whether the study had a prospective protocol or analysis plan. If a prospective analysis plan (from your funding proposal, IRB or other ethics committee submission, study protocol, or other planning document written before analyzing the data) was used in designing the study, please include the relevant document(s) with your revised manuscript as a Supporting Information file to be published alongside your study and cite it in the Methods section. A legend for this file should be included at the end of your manuscript. If no such document exists, please make sure that the Methods section transparently describes when analyses were planned, and when/why any data-driven changes to analyses took place. Changes in the analysis, including those made in response to peer review comments, should be identified as such in the Methods section of the paper, with rationale.

Response: We would like to thank the editor for the reminder. We confirm that all analyses conducted in this study follow the approved CPRD protocol as stated in the manuscript. One additional analysis was undertaken in response to the peer review comments, and this has been clearly indicated in the revised manuscript.

Under the Method Section,

*“Ethical approval was obtained from the Independent Scientific Advisory Committee of CPRD (protocol number: 23_002896), and all analyses were conducted in accordance with this study protocol approved in 2023.”* (P.14, Line 292 to 294)

*“h) not combining gabapentinoid prescriptions if they were less than or equal to 90 days apart (an unplanned sensitivity analysis);” (P.13, Line 270 to 271)*

**References**

1.	Arnold BF, Ercumen A. Negative Control Outcomes: A Tool to Detect Bias in Randomized Trials. JAMA. 2016;316(24):2597-8. doi: 10.1001/jama.2016.17700. PubMed PMID: 28027378; PubMed Central PMCID: PMCPMC5428075.

2.	Molero Y, Larsson H, D'Onofrio BM, Sharp DJ, Fazel S. Associations between gabapentinoids and suicidal behaviour, unintentional overdoses, injuries, road traffic incidents, and violent crime: population based cohort study in Sweden. BMJ. 2019;365:l2147. Epub 20190612. doi: 10.1136/bmj.l2147. PubMed PMID: 31189556; PubMed Central PMCID: PMCPMC6559335.

3.	Isoardi KZ, Polkinghorne G, Harris K, Isbister GK. Pregabalin poisoning and rising recreational use: a retrospective observational series. Br J Clin Pharmacol. 2020;86(12):2435-40. Epub 20200605. doi: 10.1111/bcp.14348. PubMed PMID: 32374500; PubMed Central PMCID: PMCPMC7688538.

4.	Rietjens SJ, Sikma MA, Hunault CC, de Lange DW, Hondebrink L. Pregabalin poisoning: Evaluation of dose-toxicity relationship. Br J Clin Pharmacol. 2022;88(3):1288-97. Epub 20211008. doi: 10.1111/bcp.15073. PubMed PMID: 34505299; PubMed Central PMCID: PMCPMC9293434.

5.	Statistics OfN. Deaths related to drug poisoning in England and Wales QMI. Office for National Statistics, 2020 13/10/2020. Report No.

6.	Daly C, Griffin E, Ashcroft DM, Webb RT, Perry IJ, Arensman E. Intentional Drug Overdose Involving Pregabalin and Gabapentin: Findings from the National Self-Harm Registry Ireland, 2007-2015. Clin Drug Investig. 2018;38(4):373-80. doi: 10.1007/s40261-017-0616-y. PubMed PMID: 29264838.

7.	Tollefsen IM, Helweg-Larsen K, Thiblin I, Hem E, Kastrup MC, Nyberg U, et al. Are suicide deaths under-reported? Nationwide re-evaluations of 1800 deaths in Scandinavia. BMJ Open. 2015;5(11):e009120. Epub 20151125. doi: 10.1136/bmjopen-2015-009120. PubMed PMID: 26608638; PubMed Central PMCID: PMCPMC4663440.

8.	Belanger F UA, Falzon A. Accidental poisoning-related deaths in an enlarged European Union Brussels: Institut de Veille Sanitaire, Unité Traumatisme – DMCT, France, 2008 June 2008. Report No.

9.	Gao L, Man KKC, Chan EW, Chui CSL, Li X, Coghill D, et al. Treatment with Methylphenidate for Attention Deficit Hyperactivity Disorder (ADHD) and the Risk of All-Cause Poisoning in Children and Adolescents: A Self-Controlled Case Series Study. CNS Drugs. 2021;35(7):769-79. Epub 20210720. doi: 10.1007/s40263-021-00824-x. PubMed PMID: 34283391; PubMed Central PMCID: PMCPMC8310501.

10.	Nechuta S, Mukhopadhyay S, Krishnaswami S, Golladay M, McPheeters M. Record Linkage Approaches Using Prescription Drug Monitoring Program and Mortality Data for Public Health Analyses and Epidemiologic Studies. Epidemiology. 2020;31(1):22-31. doi: 10.1097/EDE.0000000000001110. PubMed PMID: 31592867; PubMed Central PMCID: PMCPMC6889900.

11.	Lewer D, Brothers TD, Van Hest N, Hickman M, Holland A, Padmanathan P, et al. Causes of death among people who used illicit opioids in England, 2001-18: a matched cohort study. Lancet Public Health. 2022;7(2):e126-e35. Epub 20211211. doi: 10.1016/S2468-2667(21)00254-1. PubMed PMID: 34906332; PubMed Central PMCID: PMCPMC8810398.

12.	Whitaker HJ, Farrington CP, Spiessens B, Musonda P. Tutorial in biostatistics: the self-controlled case series method. Stat Med. 2006;25(10):1768-97. doi: 10.1002/sim.2302. PubMed PMID: 16220518.

13.	Petersen I, Douglas I, Whitaker H. Self controlled case series methods: an alternative to standard epidemiological study designs. BMJ. 2016;354:i4515. Epub 20160912. doi: 10.1136/bmj.i4515. PubMed PMID: 27618829.

14.	Yuen ASC, Chen B, Chan AYL, Hayes JF, Osborn DPJ, Besag FMC, et al. Use of gabapentinoid treatment and the risk of self-harm: population based self-controlled case series study. BMJ. 2025;389:e081627. Epub 20250430. doi: 10.1136/bmj-2024-081627. PubMed PMID: 40306941.

15.	Man KKC, Coghill D, Chan EW, Lau WCY, Hollis C, Liddle E, et al. Association of Risk of Suicide Attempts With Methylphenidate Treatment. JAMA Psychiatry Journal Translated Name JAMA Psychiatry. 2017;74(10):1048-55. doi: 10.1001/jamapsychiatry.2017.2183. PubMed PMID: 28746699; PubMed Central PMCID: PMCPMC5710471.

16.	National Institute for Health and Care Exellence. Neuropathic pain in adults: pharmacological management in non-specialist settings. 2013 22/09/2020. Report No.

17.	Excellence NIfHaC. Chronic pain (primary and secondary) in over 16s: assessment of all chronic pain and management of chronic primary pain United Kingdom: NICE; 2021 [cited 2025 27/11/2025]. Available from: <https://www.nice.org.uk/guidance/ng193/chapter/Recommendations>.

18.	U.S. Food & Drug Administration. FDA warns about serious breathing problems with seizure and nerve pain medicines gabapentin (Neurontin, Gralise, Horizant) and pregabalin (Lyrica, Lyrica CR), 2019 [updated 19 December 2019]. Available from: <https://www.fda.gov/drugs/drug-safety-and-availability/fda-warns-about-serious-breathing-problems-seizure-and-nerve-pain-medicines-gabapentin-neurontin>.

19.	Babu KM, Brent J, Juurlink DN. Prevention of Opioid Overdose. N Engl J Med. 2019;380(23):2246-55. doi: 10.1056/NEJMra1807054. PubMed PMID: 31167053.

20.	Farrington CP, Whitaker HJ, Hocine MN. Case series analysis for censored, perturbed, or curtailed post-event exposures. Biostatistics. 2009;10(1):3-16. Epub 20080521. doi: 10.1093/biostatistics/kxn013. PubMed PMID: 18499654.

21.	Man KKC, Lau WCY, Coghill D, Besag FMC, Cross JH, Ip P, et al. Association between methylphenidate treatment and risk of seizure: a population-based, self-controlled case-series study. Lancet Child Adolesc Health. 2020;4(6):435-43. doi: 10.1016/S2352-4642(20)30100-0. PubMed PMID: 32450123.

22.	Wang GH, Man KKC, Chang WH, Liao TC, Lai EC. Use of antipsychotic drugs and cholinesterase inhibitors and risk of falls and fractures: self-controlled case series. BMJ. 2021;374:n1925. Epub 20210909. doi: 10.1136/bmj.n1925. PubMed PMID: 34503972; PubMed Central PMCID: PMCPMC8427404.

23.	von Elm E, Altman DG, Egger M, Pocock SJ, Gotzsche PC, Vandenbroucke JP, et al. Strengthening the Reporting of Observational Studies in Epidemiology (STROBE) statement: guidelines for reporting observational studies. BMJ. 2007;335(7624):806-8. doi: 10.1136/bmj.39335.541782.AD. PubMed PMID: 17947786; PubMed Central PMCID: PMCPMC2034723.
